# Dissecting the Mechanism of Action of Spiperone—A Candidate for Drug Repurposing for Colorectal Cancer

**DOI:** 10.3390/cancers14030776

**Published:** 2022-02-02

**Authors:** Annamaria Antona, Marco Varalda, Konkonika Roy, Francesco Favero, Eleonora Mazzucco, Miriam Zuccalà, Giovanni Leo, Giulia Soggia, Valentina Bettio, Martina Tosi, Miriam Gaggianesi, Beatrice Riva, Simone Reano, Armando Genazzani, Marcello Manfredi, Giorgio Stassi, Davide Corà, Sandra D’Alfonso, Daniela Capello

**Affiliations:** 1 Centre of Excellence in Aging Sciences, Department of Translational Medicine, University of Piemonte Orientale, 28100 Novara, Italy; annamaria.antona@uniupo.it (A.A.); marco.varalda@uniupo.it (M.V.); francesco.favero@uniupo.it (F.F.); eleonora.mazzucco@uniupo.it (E.M.); 20016274@studenti.uniupo.it (G.L.); giulia.soggia01@universitadipavia.it (G.S.); simone.reano@uniupo.it (S.R.); marcello.manfredi@uniupo.it (M.M.); davide.cora@uniupo.it (D.C.); 2 Department of Immunology, Faculty of Biological and Veterinary Sciences, Nicolaus Copernicus University, 87-100 Torun, Poland; konkonika.roy@doktorant.umk.pl; 3 Center for Translational Research on Autoimmune and Allergic Disease (CAAD), University of Piemonte Orientale, 28100 Novara, Italy; miriam.zuccala@uniupo.it (M.Z.); martina.tosi@uniupo.it (M.T.); sandra.dalfonso@uniupo.it (S.D.); 4 Department of Health Sciences, University of Piemonte Orientale, 28100 Novara, Italy; 5 UPO Biobank, University of Piemonte Orientale, 28100 Novara, Italy; valentina.bettio@uniupo.it; 6 Department of Surgical, Oncological and Stomatological Sciences, University of Palermo, 90127 Palermo, Italy; miriam.gaggianesi@unipa.it (M.G.); giorgio.stassi@unipa.it (G.S.); 7 Department of Pharmaceutical Sciences, University of Piemonte Orientale, 28100 Novara, Italy; beatrice.riva@uniupo.it (B.R.); armando.genazzani@uniupo.it (A.G.)

**Keywords:** repurposing, phospholipase C, colorectal cancer, endoplasmic reticulum stress, intracellular calcium, lipid metabolism, psychotropic drugs, cancer stem cells, mitochondria, Golgi

## Abstract

**Simple Summary:**

Despite advances in primary and adjuvant treatments, approximately 50% of colorectal cancer (CRC) patients still die from recurrence and metastatic disease. Thus, alternative and more effective therapeutic approaches are expected to be developed. Drug repurposing is increasing interest in cancer therapy, as it represents a cheaper and faster alternative strategy to de novo drug synthesis. Psychiatric medications are promising as a new generation of antitumor drugs. Here, we demonstrate that spiperone—a licensed drug for the treatment of schizophrenia—induces apoptosis in CRC cells. Our data reveal that spiperone’s cytotoxicity in CRC cells is mediated by phospholipase C activation, intracellular calcium homeostasis dysregulation, and irreversible endoplasmic reticulum stress induction, resulting in lipid metabolism alteration and Golgi apparatus damage. By identifying new targetable pathways in CRC cells, our findings represent a promising starting point for the design of novel therapeutic strategies for CRC.

**Abstract:**

Approximately 50% of colorectal cancer (CRC) patients still die from recurrence and metastatic disease, highlighting the need for novel therapeutic strategies. Drug repurposing is attracting increasing attention because, compared to traditional de novo drug discovery processes, it may reduce drug development periods and costs. Epidemiological and preclinical evidence support the antitumor activity of antipsychotic drugs. Herein, we dissect the mechanism of action of the typical antipsychotic spiperone in CRC. Spiperone can reduce the clonogenic potential of stem-like CRC cells (CRC-SCs) and induce cell cycle arrest and apoptosis, in both differentiated and CRC-SCs, at clinically relevant concentrations whose toxicity is negligible for non-neoplastic cells. Analysis of intracellular Ca^2+^ kinetics upon spiperone treatment revealed a massive phospholipase C (PLC)-dependent endoplasmic reticulum (ER) Ca^2+^ release, resulting in ER Ca^2+^ homeostasis disruption. RNA sequencing revealed unfolded protein response (UPR) activation, ER stress, and induction of apoptosis, along with IRE1-dependent decay of mRNA (RIDD) activation. Lipidomic analysis showed a significant alteration of lipid profile and, in particular, of sphingolipids. Damage to the Golgi apparatus was also observed. Our data suggest that spiperone can represent an effective drug in the treatment of CRC, and that ER stress induction, along with lipid metabolism alteration, represents effective druggable pathways in CRC.

## 1. Introduction

According to the recent GLOBOCAN statistics, colorectal cancer (CRC) represents nearly 1 out of 10 cancer cases and deaths worldwide. With more than 1.9 million new cases and 935,000 deaths in 2020, CRC ranks third in terms of incidence and second in terms of mortality [1]. Despite the emergence of several screening programs to reduce the incidence of CRC, nearly a quarter of CRC cases are diagnosed at an advanced stage with metastases, and as many as 50% of patients who present with non-metastatic CRC develop metachronous metastases, resulting in difficulties in curative surgical control and subsequent tumor-related deaths—particularly in elderly patients [2,3].

Although with the advances in primary and adjuvant treatments, the cure and the survival time in CRC have been improving [4,5], approximately 50% of patients still die from recurrence and metastatic disease [6,7,8]. Indeed, the 5-year relative survival rate for CRC is 90% for patients diagnosed with localized disease, but drops to 14% for those diagnosed with stage IV disease [9]. These figures indicate that further investigation is still required in order to develop alternative and effective approaches for medical intervention, especially in advanced stages and in the most vulnerable patients.

Currently, radiotherapy and cytostatic drugs still represent the backbone of CRC treatment, interfering with cell proliferation [10]. In more recent years, novel types of cancer treatment have been developed and have joined standard chemotherapy. These include targeted therapies via small-molecule inhibitors or monoclonal antibodies that target signal transduction and, lately, novel types of immunotherapies targeting cancer cells, the tumor microenvironment, or stimulating antitumor immune response [11,12]. Although several of these approaches are promising [13,14], like standard chemotherapy, they can generate severe adverse events, as well as the emergence of resistance.

The failure of CRC treatment, as with the majority of solid tumors, can be attributed to cancer stem cells (CSCs)—a subpopulation of tumor cells with self-renewal and multi-lineage differentiation capabilities, which are predisposed to drive tumor progression and the emergence of therapy-resistant clones, ultimately leading to recurrence and metastasis, resulting in patient mortality [15,16,17]. Thus, alternative cell death pathways capable of killing therapy-resistant CRC-SCs have gained considerable interest.

Despite the tremendous progress made in the molecular deciphering of signaling pathways and the identification of novel molecular targets for cancer treatment, successful cancer drug development has proven difficult due to the enormous time and financial investment required for the introduction of novel compounds into the clinic and the high attrition rate [18,19,20]. For oncology drugs that receive marketing approval, prices have risen steeply in recent years, with an extremely negative impact on both the economy and society—particularly for oncological patients [21]—and there is an increasing recognition that the budgets of most national healthcare services will soon be unable to further sustain the increasing trend in costs of new oncological drugs [22]. Drug repurposing is a strategy for identifying new therapeutic indications for approved or investigational drugs that are outside the scope of the original development [23,24]. Drug repurposing has attracted increasing attention compared with the traditional de novo drug discovery pipeline, which is more time-consuming and expensive [23,25,26]. Repurposing can contribute to the identification of new therapies for diseases at a lower cost and in a shorter time, particularly in those cases where preclinical safety studies have already been completed, reducing the need for additional research to investigate pharmacokinetic properties and toxicity [23,27]. Moreover, repurposed drugs may reveal new targets and pathways that can be further exploited. Thus, drug repurposing is a promising approach in oncology for the development of new treatments [28,29,30,31,32].

We have recently reported the cytotoxic effect of spiperone—a neuroleptic drug licensed for the treatment of schizophrenia [33]—at clinically relevant concentrations on breast cancer and CRC cell lines [34]. In this study, through in vitro and omics analysis, we demonstrate that spiperone proves effective in killing CRC cells and CRC-SCs by disrupting intracellular Ca^2+^ kinetics and inducing ER stress, lipid metabolism dysregulation, and damage to the Golgi apparatus (GA). Altogether, our results provide evidence that spiperone displays anticancer effects at clinically relevant concentrations, and that induction of ER stress along with lipid metabolism disruption could be considered as a potential druggable pathway in CRC.

## 2. Materials and Methods

### 2.1. Cell Culture

The human differentiated HCT116, SW620, HCT8, and MDA-MB-231 cell lines were purchased from the American Type Culture Collection (ATCC), while the human dermal fibroblast cell line (hDF1) was a kind gift from Dr. Barbara Azzimonti (Department of Health Sciences, University of Piemonte Orientale, Novara, Italy) and the SW480 cell line was kindly provided by Dr. Marco De Andrea (Department of Health Sciences, University of Piemonte Orientale). vAT-MSCs were isolated from primary samples of visceral adipose tissue obtained in collaboration with Prof. Sergio Gentilli (AOU Maggiore della Carità, Novara, approved by Local Ethical Committee protocol 74/CE study number CE 10/21). HCT116, HDF, vAT-MSC, and SW480 cells were cultured in Dulbecco’s Modified Eagle Medium (DMEM, Gibco, Waltham, MA, USA), whereas HCT8, SW620, and MDA-MB-231 cells were maintained in RPMI-1620 (Gibco, Waltham, MA, USA). All culture media were supplemented with 10% fetal bovine serum (FBS, Euroclone, Milan, Italy) and 1% antibiotics–antimycotics (penicillin, streptomycin, and amphotericin; Sigma, Saint Louis, MO, USA).

Peripheral blood mononuclear cells (PBMCs) were isolated from human blood by Ficoll density gradient centrifugation and were stimulated for 24 h with 10 μmol/L phytohemagglutinin (PHA, Saint Louis, MO, USA) in RPMI-1620 (Gibco, Waltham, MA, USA) supplemented with 10% heat-inactivated FBS (Euroclone, Milan, Italy). Subsequently, cells were cultured for 48 h in fresh RPMI containing interleukin 2 (IL-2, PeproTech, Cranbury, NJ, USA). Lastly, cells were harvested and plated for viability assay.

Human CRC stem cell lines (CRC-SCs) CRC-SC#1, CRC-SC#2, CRC-SC#3, and CRC-SC#4 were kindly provided by Prof. Giorgio Stassi (Department of Surgical, Oncological and Stomatological Sciences, University of Palermo). These cell lines were cultured in suspension as colonospheres in stem cell medium (DMEM/F12, Gibco, Waltham, MA, USA) supplemented with EGF (10 μg/mL, PeproTech, Cranbury, NJ, USA) and FGF (20 μg/mL, PeproTech, Cranbury, NJ, USA), B27 and N2 (Gibco, Waltham, MA, USA), 1 mmol/L nicotinamide (Sigma-Aldrich, Saint Louis, MO, USA) and 1% antibiotics–antimycotics (penicillin, streptomycin, and amphotericin; Sigma-Aldrich, Saint Louis, MO, USA). Dedifferentiated HCT116 (dd-HCT116) cells were obtained from HCT116 cells cultured in suspension in a stem cell medium for two consecutive weeks.

All of the cell lines were cultured under a controlled temperature and atmosphere in a humidified incubator (37 °C, 5% CO_2_).

### 2.2. siRNA Transfections

HCT116 cells were transfected with 30 pmol of Silencer Select Negative Control siRNA (Thermo Fisher Scientific, Waltham, MA, USA) and Silencer Select Pre-Designed siRNA (Thermo Fisher Scientific, Waltham, MA, USA) targeting the following genes: DDIT3, PLCB1, PLCB3, PLCG1, PLCD3, and PLCE1. The related sequences (5′–3′) are listed in Table 1. Transfection was performed using Lipofectamine 3000 (Thermo Fisher Scientific, Waltham, MA, USA). Cells were incubated with the desired siRNA for 24 h to allow transfection, and were plated 72 h post-transfection in order to allow gene silencing. Silencing was monitored by RT-qPCR and/or Western blot.

### 2.3. MTT (Thiazolyl Blue Tetrazolium Bromide) Viability Assay and Viability Rescue Experiments

For each cell line, 1000 cells/well were plated (PBMCs 10,000 cells/well were plated) in a final volume of 100 μL/well in a 96-well plate. Cells were treated with different concentrations of drug and incubated for 72 h, and the same concentration of vehicle (DMSO) was used as a control. MTT (thiazolyl blue tetrazolium bromide; Sigma-Aldrich, Saint Louis, MO, USA) at 0.5 mg/mL was then added to each well and incubated for 4 h at 37 °C and 5% CO_2_. Crystals were dissolved using 100 μL of acidic isopropanol (2 N HCl), and the absorbance (570 nm and 650 nm) was read using a spectrophotometer (Victor, PerkinElmer, Waltham, MA, USA). Considering the IC_50_ determined at 72 h (~7 μmol/L), a drug concentration of 10 μmol/L was chosen to perform 24 h experiments according to the biological effects investigated and the sensitivity of the analysis methods used.

To perform viability rescue experiments, 2500 cells were plated in each well of a 96-well plate and pretreated with inhibitors for 30 min. Subsequently, 5, 10, or 20 μmol/L spiperone or vehicle alone (DMSO) was added, and MTT viability assay was performed after 24 h of co-treatment, as previously described.

### 2.4. Extreme Limiting Dilution Assay (ELDA)

CRC-SC#1 cells were plated at 6, 3, or 1 cell/well in an ultralow-attachment 96-well plate in 50 μL of complete stem cell medium, and then treated with 50 μL of medium containing 1 μmol/L spiperone or the same concentration of vehicle. Tumor spheres’ growth was monitored using a phase-contrast microscope, and colony numbers and dimensions were assessed 15 days after plating. Data were analyzed using the free online ELDA software (http://bioinf.wehi.edu.au/software/elda/, accessed on 11 May 2021).

### 2.5. Apoptosis Assay

Cells were plated in a 24-well plate (70,000/well), treated with different doses of spiperone, and analyzed at different time points. For apoptosis rescue experiments, cells were treated with different doses of spiperone alone or in combination with BAPTA-AM at 10 μmol/L or U73122 at 1 μmol/L for 24 h. For analysis, cells were stained with Annexin/propidium iodide (Ax/PI) following the manufacturer’s instructions (AdipoGen, San Diego, CA, USA). Briefly, cells were incubated at room temperature for 10 min with Ax binding buffer (10 mmol/L HEPES/NaOH, pH 7.4, 140 mmol/L NaCl, 2.5 mmol/L CaCl_2_) containing Ax V-FITC. Next, cells were washed and resuspended in Ax binding buffer. PI was added to all of the samples 5 min before FACS analysis (Attune NxT, Flow Cytometer, Thermo Fisher Scientific, Waltham, MA, USA). Data were analyzed with FlowJo, LLC software v 10 (Ashland, OR, USA).

### 2.6. Cell Cycle Assay

Cell cycle analysis was performed by quantification of cellular DNA content via flow cytometry. HCT116 cells were plated in a 12-well plate (60,000 cells/well) and starved for 16 h (DMEM without FBS). After starvation, cells were treated with spiperone for 24 and 48 h in DMEM supplemented with 10% FBS. Subsequently, cells were harvested and fixed with 70% ethanol for 30 min. Then, cells were treated with 20 μg/mL RNAse A (Sigma-Aldrich, Saint Louis, MO, USA) for 45 min at 37 °C. Finally, cells were stained with PI (50 μg/mL, Sigma), and their fluorescence was acquired through a cytofluorimeter (Attune NxT, Flow Cytometer, Thermo Fisher Scientific, Waltham, MA, USA). Data analysis was performed using FlowJo, LLC software v 10 (Ashland, OR, USA).

### 2.7. Phospholipidosis Assay

HCT116, SW620, and HCT8 cells were plated at a concentration of 30,000 cells/well in a 48-well plate, treated for 16 h with 5 and 10 μmol/L spiperone—or 5 μmol/L fluoxetine, as a positive control—and stained with 1× LipidTOX Green (Thermo Fisher Scientific). Then, nuclei were stained using Hoechst 33342 (5 μg/mL) and plates were incubated for 30 min in the dark at 37 °C. Subsequently, cells were washed with PBS and fixed with 4% paraformaldehyde for 15 min in the dark. Signals were acquired with a fluorescence microscope (FLoid Cell Imaging Station, Life Technology, Carlsbad, CA, USA), and images were analyzed using ImageJ software v 1.52a.

### 2.8. Intracellular Calcium Measurements

To investigate Ca^2+^ concentration, 300,000 cells were harvested for each condition, washed with PBS 1×, and resuspended in 100 μL of Krebs–Ringer buffer (KRB) containing 135 mmol/L NaCl, 5 mmol/L KCl, 0.4 mmol/L KH_2_PO_4_, 1 mmol/L MgSO_4_, 20 mmol/L HEPES, and 2 mmol/L CaCl2 with 2.5 μmol/L Fluo-4 AM (Molecular Probes, Invitrogen, Waltham, MA, USA) or with 2.5 μmol/L of Indo-1 AM (eBioscience, Invitrogen, Waltham, MA, USA), along with 2.5 μmol/L of Mag-Fluo-4 AM (Invitrogen, Waltham, MA, USA), at room temperature in the dark for 30 min. Each condition was then washed with KRB and re-incubated in 2 mmol/L CaCl_2_ KRB at room temperature for 20 min. Subsequently, all of the samples were resuspended for 30 min in 2 mmol/L CaCl_2_ KRB, 2 mmol/L EGTA KRB, or 2 mmol/L EGTA KRB with 10 μmol/L U-73122 (Sigma-Aldrich, Saint Louis, MO, USA), 10 μmol/L U-73443, 50 μmol/L 2APB, 50 µmol/L genistein, 10, 20, and 50 μmol/L regorafenib, 10 µmol/L CID-1067700, 10 µmol/L gallein, 10 µmol/L YM-254890 (Cayman Chemicals), or 50 µmol/L suramin (Cayman Chemicals, Ann Arbor, MI, USA). Finally, fluorescence emission was acquired for each sample, and an average of 300 cells each second was recorded by flow cytometry (FACS Symphony, BD Biosciences, Franklin Lakes, NJ, USA). After basal fluorescence acquisition, cells were stimulated with different doses of spiperone, histamine (Cayman Chemicals), or thapsigargin 5 µmol/L (Cayman Chemicals, Ann Arbor, MI, USA) in 2 mmol/L CaCl_2_ KRB or 2 mmol/L EGTA KRB. Data analysis was performed using FlowJo, LLC software v 10 (Ashland, OR, USA).

### 2.9. Immunofluorescence Microscopy Analysis

HCT116 and CRC-SC#1 cells at a concentration of 50,000 cells/well were seeded onto glass coverslips and treated with 5 or 10 μmol/L spiperone—alone, or in co-treatment with BAPTA-AM at 10 μmol/L, 4-PBA at 10 μmol/L, or U73122 at 1 μmol/L, for 16 h. After the treatment, cells were washed with PBS and fixed with 4% PFA for 10 min at room temperature, and then washed again with PBS. Then, cells were permeabilized by incubation with cold HEPES-Triton X-100 (20 mmol/L HEPES pH 7.4, 300 mmol/L sucrose, 50 mmol/L NaCl, 3 mmol/L MgCl_2_, 0.5% Triton X-100) for 5 min at 4 °C. Cells were washed with 0.4% PBS-BSA and saturated using 4% PBS-BSA for 30 min before placing primary antibodies. The antibodies used in these experiments were anti-cathepsin B (Cell Signaling Technology, Danvers, MA, USA), anti-CHOP (Cell Signaling Technology, Danvers, MA, USA), anti-LAMP1 (Santa Cruz Biotechnology, Dallas, TX, USA), and anti-GOLGIN97 (Invitrogen, Waltham, MA, USA). Cells were incubated with primary antibodies for 30 min, then washed with 4% PBS-BSA and incubated with secondary antibodies conjugated with Alexa Fluor-488, −536 (Invitrogen, Waltham, MA, USA), and DAPI for 30 min. After the incubation, glasses were mounted on glass slides using Mowiol (20% Mowiol 4–88, 2.5% DABCO in PBS, pH 7.4).

For [Ca^2+^]_ER_ fluorescence microscopy experiments, after treatment, cells were stained with Hoechst 33342 (5 μg/mL) and incubated for 30 min in the dark at 37 °C. Then, cells were incubated for 30 min with Mag-Fluo-4 AM 2.5 µmol/L at room temperature in the dark. After the staining, cells were fixed with 1% PFA and mounted on glass slides using Mowiol (20% Mowiol 4–88, 2.5% DABCO in PBS, pH 7.4)

Images were acquired using a Leica TCS SP8 confocal microscope or DM5500B fluorescence microscope (Leica, Wetzlar, Germany), and analyzed using ImageJ software v 1.52a (Ashland, OR, USA).

### 2.10. Western Blotting

HCT116, CRC-SC#1, HCT8, and SW480 cells were plated at a concentration of 300,000 cells/well in 6-well plates and treated with 5 or 10 μmol/L spiperone for the indicated time. For the autophagic flux experiment, two conditions were carried out for each treatment: drug alone, and co-treatment with spiperone 5 or 10 μmol/L and chloroquine 50 μmol/L. After treatments, whole-cell lysates were prepared using RIPA lysis buffer (25 mmol/L HEPES pH 8, 135 mmol/L NaCl, 5 mmol/L EDTA, 1 mmol/L EGTA, 1 mmol/L ZnCl_2_, 50 mmol/L NaF, 1% Nonidet P40, 10% glycerol) with protease inhibitors (AEBSF, aprotinin, bestatin, E-64, EDTA, leupeptin; Sigma-Aldrich, Saint Louis, MO, USA) and orthovanadate. Lysates were then kept on a wheel for 20 min at 4 °C and after centrifuged at 12,500× *g* for 15 min. Proteins contained in the samples were collected and quantified using the Pierce BCA Protein Assay Kit (Thermo Fisher Scientific, Waltham, MA, USA). Successively, proteins were denatured at 95 °C for 5 min in the presence of 2% sodium dodecyl sulfate (SDS), 150 mmol/L dithiothreitol (DTT), and 0.01% bromophenol blue. Electrophoresis of the samples was performed using 6, 8, 10, or 15% polyacrylamide gels, and proteins were transferred from the gel to a polyvinylidene difluoride membrane (PVDF; Amersham, Buckinghamshire, UK). Lastly, the membrane was saturated using 3% bovine serum albumin (BSA; Sigma, Saint Louis, MO, USA) in TBS/Tween-20 0.1% (Tris-buffered saline 1 × containing 50 mmol/L Trizma base, 120 mmol/L NaCl, 0.1% polyethylene glycol sorbitan monolaurate (Tween-20)) for 1 h and incubated with primary antibody dissolved in the same buffer with sodium azide 0.01%. Primary antibodies were anti-LC3B (Thermo Scientific, Waltham, MA, USA), anti-P-P70S6K T389, anti-P70S6K, anti-P-S6 S235/236, anti-S6, anti-P-AMPKa T172, anti-AMPK (Cell Signaling Technology, Danvers, MA, USA), anti-P-eIF2α (S51), anti-eIF2α (Santa Cruz Biotechnology, Dallas, TX, USA), anti-ATF4 (Cell Signaling Technology, Danvers, MA, USA), anti-ATF6 (Cell Signaling Technology, Danvers, MA, USA), anti-P-IRE1α (S724) (Thermo Fisher Scientific, Waltham, MA, USA), anti-IRE1α (Cell Signaling Technology, Danvers, MA, USA), anti-CHOP (Cell Signaling Technology, Danvers, MA, USA), anti-Phospho-(Ser) PKC substrate (Cell Signaling Technology, Danvers, MA, USA), and anti-GAPDH (Cell Signaling Technology, Danvers, MA, USA). The next day, the primary antibody was removed, and the membrane was washed with 0.1% TBS-Tween-20 for 15 min three times, and then incubated with horseradish-peroxidase-conjugated secondary anti-mouse or anti-rabbit antibody (PerkinElmer Life Science, Waltham, MA, USA) diluted 1:3000 in TBS-Tween-20 0.1% for 45 min. After washing, reading of the membrane was performed using ECL Western Lightning Chemiluminescence Reagent Plus (PerkinElmer Life Science, Waltham, MA, USA) and images acquired with the ChemiDoc Touch (Bio-Rad, Hercules, CA, USA).

### 2.11. Mitochondrial Membrane Potential Analysis

HCT116 cells were plated at a concentration of 30,000 cells/well in a 48-well plate and treated with 5 or 10 μmol/L spiperone for the desired time; 0.10% DMSO was used as a negative control. After treatment, cells were stained with 10 μg/mL JC-1 dye (AdipoGen, San Diego, CA, USA) in PBS for 30 min in the dark at 37 °C. FCCP (Cayman chemicals, Ann Arbor, MI, USA) was added for 15 min after the staining as a positive control. Signals were acquired with a fluorescence microscope (FLoid Cell Imaging Station, Life Technology, Carlsbad, CA, USA) and images were analyzed using ImageJ software v 1.52a, calculating the red/green fluorescence ratio. For mitochondrial membrane depolarization rescue experiments, HCT116 cells were pretreated with BAPTA-AM at 10 μmol/L and the MCU inhibitor Ru360 at 10 μmol/L for 30 min, and then treated with spiperone 10 μmol/L for 3 h.

### 2.12. Intact Cell Respiration Using High-Resolution Respirometry

We determined cellular respiration via high-resolution respirometry using the substrate, uncoupler, inhibitor, titration (SUIT) protocols. A total of 2,000,000 HCT116 cells were treated for 1 and 6 h with 10 μmol/L spiperone or the same concentration of vehicle (DMSO). At the end of the treatments, 2,000,000 HCT116 cells were trypsinized, centrifuged at 300× *g* for 5 min, resuspended in MiR05 mitochondrial respiration medium (0.5 mmol/L EGTA, 3.0 mmol/L MgCl_2_ 6H_2_O, 60 mmol/L potassium lactobionate, 20 mmol/L taurine, 10 mmol/L KH_2_PO_4_, 20 mmol/L HEPES, 110 mmol/L sucrose, 1 g/L bovine serum albumin, pH 7.1), and transferred to an Oxygraph-2K high-resolution respirometer (Oroboros Instruments, Innsbruck, Austria). Control and treated samples were assessed simultaneously. After initial stabilization of O_2_ flux, pyruvate (5 mmol/L) was used to sustain TCA-linked respiration. An ATP synthetase inhibitor, oligomycin (O), was added at a final concentration of 5 nmol/L, and oxygen consumption was quantified to determine the oligomycin-sensitive and -insensitive respiration. A protonophore (H^+^ ionophore) and uncoupler of oxidative phosphorylation, FCCP (U), was then added in 0.5 μmol/L increments to achieve maximum respiration, in order to quantify maximum respiratory capacity. This was followed by rotenone (Rot) at a final concentration of 500 nmol/L, in order to inhibit complex I of the electron transport chain (ETC), and then 2.5 μmol/L antimycin A (Aa), which inhibits complex III, was added to determine the non-mitochondrial respiration (ROX). Oxygen consumption rates were calculated using accompanying software (DatLab7, Oroboros).

### 2.13. RNA Extraction and Real-Time PCR

To perform RNA extraction, HCT116 and CRC-SC#1 cells were plated (300,000 in each well) and treated with different concentrations of spiperone for different periods of time. After the treatment, RNA was extracted using the phenol/chloroform method (RNAzol, Sigma-Aldrich, Saint Louis, MO, USA) and isopropanol precipitation, following the manufacturer’s instructions. Then, precipitated RNA was washed with 75% ice-cold ethanol and resuspended in water. RNA samples were quantified using a NanoDrop 2000 and then reverse-transcribed into cDNA using recombinant Moloney murine leukemia virus reverse transcriptase (MultiScribe Reverse Transcriptase, Bio-Rad, Hercules, CA, USA) and the iScript cDNA Synthesis Kit (Bio-Rad, Hercules, CA, USA). The genes analyzed by real-time PCR using the SsoAdvanced Universal SYBR Green Supermix Kit (Bio-Rad) are reported in Table 2. *HPRT* and *GUSB* were used as control genes. Relative quantification was determined using the ΔΔCt algorithm [35].

### 2.14. XBP1 Splicing Variant Polymerase Chain Reaction (PCR)

To evaluate XBP1 alternative splicing cDNA was used as a template for PCR amplification using XBP1-specific primers (forward: 5′TTACGAGAGAAAACTCATGGCC3′; reverse: 5′GGGTCCAAGTTGTCCAGAATGC3′). PCR was performed using Taq polymerase (Bio-Rad, Hercules, CA, USA), and the products were separated by agarose gel electrophoresis and visualized with GelGreenTM (Invitrogen, Waltham, MA, USA). Results were acquired at ChemiDoc touch (Bio-Rad, Hercules, CA, USA).

### 2.15. Transcriptomic Analysis (RNA Extraction, Sequencing, and Bioinformatic Analysis)

Extraction of total RNA from CRC-SC#1 and HCT116 cells was performed using the miRNeasy^®^ Mini Kit (Cat No./ID: 217004 QIAGEN, Venlo, The Netherlands), according to the manufacturer’s instructions. Libraries were prepared using the Illumina TruSeq Stranded mRNA Kit (Cat. # RS-122-2101) according to the manufacturer’s protocol, starting with 0.5 μg total RNA derived from cell lines. Quantification of total RNA was performed using the Qubit™ RNA HS Assay Kit (Thermo Fisher Scientific, Waltham, MA, USA—Cat No./ID: Q32852), and integrity of total RNA was assessed by High-Sensitivity RNA ScreenTape Assay for the 4200 TapeStation System (Agilent Technologies, Santa Clara, CA, USA), with a quality score (RIN—RNA Integrity Number) >8. Poly-A RNA molecules were captured by poly-T oligo magnetic beads. mRNAs were fragmented by cleavage with divalent cations and converted to cDNA with reverse transcriptase and random primers. The resulting cDNAs were converted to double-stranded cDNAs and ligated with the adapter. A high-quality library showed a single symmetric peak with a range between 250 and 350 bp for cluster generation and sequencing. A total of 1.8 pM of pooled libraries (with 1% of PhiX) were processed using an Illumina NextSeq 550 sequencer (Illumina, San Diego, CA, USA) on the NextSeq 500/550 High-Output Kit v2.5 (150 Cycles—2 × 75 read length, paired-ends), obtaining a mean of 50 million 75 bp paired-end reads per sample.

Two biological replicates from each cell line and for each condition were analyzed for a total of 8 samples. The STAR program [36] was used to align reads to the hg38 human reference genome. Annotations provided by the Ensembl v100 database were set as a reference for the RSEM computational pipeline [37] used for quantification of gene and isoform expression levels. Only genes with a TPM > 2 in at least one sample were considered to be expressed, and underwent further analysis. Statistical and graphical computations were performed in the R environment (www-r-project.org, accessed on 7 April 2021). PCA plotting was performed using the “prcomp” package of R on the matrix of expression data. Differentially expressed genes (DEGs) were calculated using DESeq2 [38] with |log2FC| > 1.5 and p.adj < 0.05 as parameters to define the statistical significance of differential gene expression. Heatmaps showing unsupervised hierarchical clustering of genes were produced using the “pheatmap” package of R; expression values are expressed as TPM. Venn diagrams were obtained using the web-based tool InteractiVenn [39]. Characterization of activated pathways in modulated genes was computed using IPA Ingenuity (QIAGEN Inc., https://www.qiagenbioinformatics.com/products/ingenuitypathway-analysis, accessed on 7 April 2021). GO terms associated with DEGs were analyzed using ToppGene Suite [40] software, filtering only the GO terms with Bonferroni *p*-value < 0.05. Semantic plots were produced using Revigo software [41], setting the analysis to *Homo sapiens* and using default parameters.

In order to map computationally predicted CHOP/DDIT3-binding sites on the promoter regions of our identified DEGs, human GRCh38/hg38 coordinates of the transcription start site (TSS) for the 158 final candidate genes were downloaded from the Ensembl database. Full protein-coding transcripts associated with a known RefSeq ID were retained and used in the UCSC Table Browser tool to fetch a genomic region of (−1000, +1000) nts of DNA around their TSS and, thus, define a promoter region for the corresponding genes. The presence of the CHOP/DDIT3-binding sites was then investigated by means of two different strategies: (1) searching for the presence of the consensus CHOP/DDIT3 sequence TGCAAT [42], and (2) annotation of DDit3::Cebpa-binding sites according to the genome-wide mapping of transcription-factor-binding sites performed using the Jaspar 2022 database [43] (MA0019.1 PWM with top 10% score).

### 2.16. Lipidomic Analysis (Extraction, Analysis, and Processing)

HCT116 and CRC-SC#1 cells were plated at the concentration of 500,000 cells/well and subjected to lipid extraction using a biphasic method. To the pellets of cells, we added 100 µL of cold MeOH containing a mixture of deuterated standard (SPLASH LIPIDOMIX^®^) of 5%. The solution was vortexed for 10 s, followed by the addition of 1000 µL of a cold solution of isopropyl alcohol IPA/H2O 75/25 (*v*/*v*), and vortexed for 30 s. The samples were then sonicated for 2 min to promote cell lysis and vortexed for 30 s. The tube was then placed in a rotator mixer at 4 °C for 30 min at 40 rpm, and then at 4 °C for 30 min. The tubes were then centrifuged at 3500× *g* at 4 °C for 10 min, and then 1000 μL of supernatant was transferred to an Eppendorf tube and dried in a SpeedVac at 35 °C. Samples were resuspended in 100 µL of MeOH with 12.5 ng/mL internal standard (CUDA standard), and then analyzed using a UHPLC Vanquish system (Thermo Scientific, Rodano, Italy) coupled with an Orbitrap Q-Exactive Plus (Thermo Scientific, Rodano, Italy). The separation of lipids was achieved using a reverse-phase column (Hypersil Gold™ 150 × 2.1 mm, particle size 1.9 µm). The column was maintained at 45 °C at a flow rate of 0.260 mL/min. For the positive ESI mode, mobile phase A consisted of acetonitrile/water 60/40 (*v*/*v*) with ammonium formate (10 mmol/L) and 0.1% formic acid, while mobile phase B consisted of isopropanol/acetonitrile 90/10 (*v*/*v*) with ammonium formate (10 mmol/L) and 0.1% formic acid; meanwhile, in the negative ESI mode, the organic solvents for both mobile phases were the same as in the positive mode, except for using ammonium acetate (10 mmol/L) as a mobile-phase modifier. The gradient used was as follows: 0–2 min from 30% to 43% B, 2–2.1 min from 43% to 55% B, 2.1–12 min from 55% to 65% B, 12–18 min from 65% to 85% B, and 18–20 min from 85% to 100% B; 100% B was maintained for 5 min, and then the column was allowed to re-equilibrate at 30% B for another 5 min. The total run time was 30 min.

Mass spectrometry analysis was performed in both positive and negative ion modes. The source voltage was maintained at 3.5 kV in [44] the positive ion mode and 2.8 kV in the negative ion mode. All other interface settings were identical for the two types of analysis. The capillary temperature, sheath gas flow, and aux-iliary gas flow were set at 320 °C, 40 arb, and 3 arb, respectively; the S-lens was settled at 50 rf. Data were collected in a data-dependent (ddMS2) top 10 scan mode. Survey full-scan MS spectra (mass range m/z 80 to 1200) were acquired at a resolution of R = 70,000 and AGC target 1 × 10^6^. MS/MS fragmentation was performed using high-energy C-trap dissociation (HCD) at a resolution of R = 17,500 and AGC target 1 × 10^5^. The stepped normalized collision energy (NCE) was set to 15, 30, and 45. The injection volume was 3 µL. Lock mass and regular inter-run calibrations were used for accurate mass-based analysis. An exclusion list for background ions was generated by analyzing the same procedural blank sample, both for the positive and negative ESI modes.

The acquired raw data from the untargeted analysis were processed using MSDIAL software (Yokohama City, Kanagawa, Japan), version 4.24 [44]. This included the detection of peaks, MS2 data deconvolution, compound identification, and the alignment of peaks through all of the samples. For identification, a cutoff value of 85% was selected; this value was based on 6 different similarity scores: 1 for retention time, 1 for *m*/*z*, 1 for isotopic pattern, and 3 for MS/MS (dot product, dot product reversed, and presence). Peaks corresponding to internal standards were removed from MS-DIAL-detected features and were analyzed using the Skyline program to evaluate their reproducibility. The dataset containing *m*/*z* values, retention time, peak area, and annotation from the aligned files was exported as an Excel file and manually checked to eliminate signals from blanks or wrong records. For quantification, the peak area for different detected molecular species for each particular lipid was combined (e.g., [M + NH4]+ and [M + Na]+ for TG).

Finally, an in-house library of standards was also used for identification of lipids. Statistical analysis was performed using the MetaboAnalyst 5.0 software (www.metaboanalyst.ca, accessed on 6 October 2021). For comparative analysis, peak intensities were expressed as relative percentages. Lipids were classified according to the principal classes and subclasses, and the mean normalized values in treated cells and controls (derived from the three biological replicates) were used to determine the fold change (FC). Student’s *t*-test was used to evaluate significant differences between treated samples and controls.

Fisher’s exact test was used to compare the observed numbers of species for each class, with significant variation from that expected by chance. A lipid species was considered to be significantly modulated when characterized by an FC > 1.5, and *p*-values < 0.05 in at least one treatment time).

### 2.17. Depmap Omics Data Analysis

Datasets of gene expression (Expression 21Q4 public) and of drug sensitivity (PRISM repurposing primary screen 19Q4) were downloaded from the Broad Institute’s DepMap web portal (https://depmap.org/portal/download/ accessed on 20 January 2022). The sensitivity of 34 colorectal cancer cell lines was displayed using GraphPad Prism 8.

## 3. Results

### 3.1. Spiperone Is Cytotoxic to CRC Cells and Impairs the Clonogenic Potential of CRC-SCs

We have recently reported the cytotoxic effects of the antipsychotic spiperone (also known as spiroperidol [45]) at clinically relevant concentrations (IC_50_ < 10 μmol/L) on MCF7 breast cancer and HCT116 and SW620 CRC cell lines [34]. Here, we further confirm the antineoplastic activity of this compound on CRC cell lines, and validate its specific activity against neoplastic cells. CRC cells exposed to spiperone for 72 h showed a dose-dependent reduction in viability, with an IC_50_ < 10 μmol/L. On the other hand, spiperone toxicity was negligible for non-neoplastic PBMCs (IC_50_ > 30 μmol/L), primary human dermal fibroblasts (hDF1; IC_50_ > 90 μmol/L), and visceral adipose-tissue-derived mesenchymal stem cells (vAD-MSCs; IC_50_ > 80 μmol/L (Table 3, Supplementary Figure S1a,c).

CRC cells with stem-like features (CRC-SCs) are a subset of cells within the tumor with the potential for self-renewal and resistance to therapy, and as a major cause of cancer recurrence and metastasis, represent a primary target for novel therapeutic approaches. Therefore, we investigated the efficacy of spiperone on dedifferentiated HCT116 cells (dd-HCT116) grown as colonospheres in CSC medium, and on four different CRC-SC lines derived from human primary tumors. A significant reduction in cell viability was observed in both CRC-SCs and in dd-HCT116 cells, with an overall efficacy higher than that observed in differentiated cancer cell lines, and with an IC_50_ < 5 μmol/L in 3 out of 4 CRC-SC samples (Table 3, Supplementary Figure S1b). Moreover, the in silico analysis of the antitumor activity of spiperone with the PRISM repurposing primary screen 19Q4 (www.metaboanalyst.ca, accessed on 20 January 2022) revealed that 50% of investigated CRC cell lines were sensitive to overnight treatment with 2.5 μmol/L spiperone, with a mean Log2 fold change relative to controls of −0.2937 (Supplementary Figure S2).

**Figure 1:**
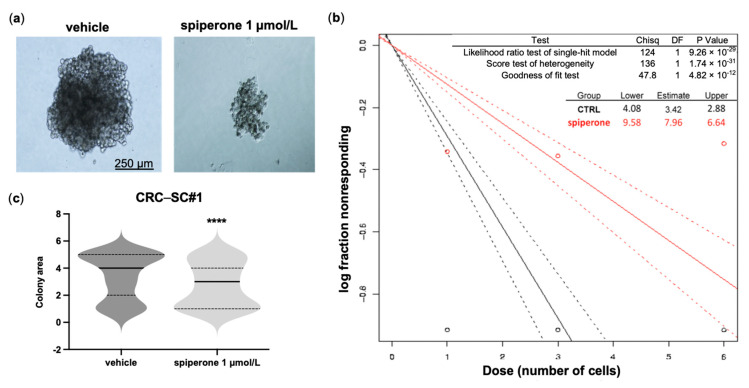
In vitro analysis of CRC-SCs’ self-renewal using the limiting dilution assay. CRC-SCs were dissociated to single cells and plated into 96-well plates. The number of wells containing spheres was then evaluated along with the size of each colonosphere. A sphere formation assay was performed on CRC-SC populations to evaluate their stemness and clonogenic potential. Representative image of CRC-SC#1 colonospheres (vehicle vs. treated) (**a**). Output of the ELDA software: the amount of initially seeded cells (*x*-axis) is plotted against the log fraction of wells without any detected spheres (*y*-axis). The slope of the line represents the log-active cell fraction (**b**). Distribution of colonospheres’ size (vehicle vs. treatment) (**c**). Data represent the mean ± SD from three independent experiments; ****: Student’s *t*-test *p* < 0.0001.

The efficacy of spiperone against colonospheres prompted us to assess the effects of the drug on the clonogenic potential of CRC-SCs. Extreme limiting dilution assay (ELDA) demonstrated a significant reduction in the dimensions and formation of colonospheres in CRC-SC#1 cells treated with 1 μmol/L spiperone, with an estimated stem cell frequency reduction from 1/3.42 in controls to 1/7.96 in treated cells (Figure 1).

### 3.2. Spiperone Induces Cell Cycle Arrest and Apoptotic Cell Death

**Figure 2:**
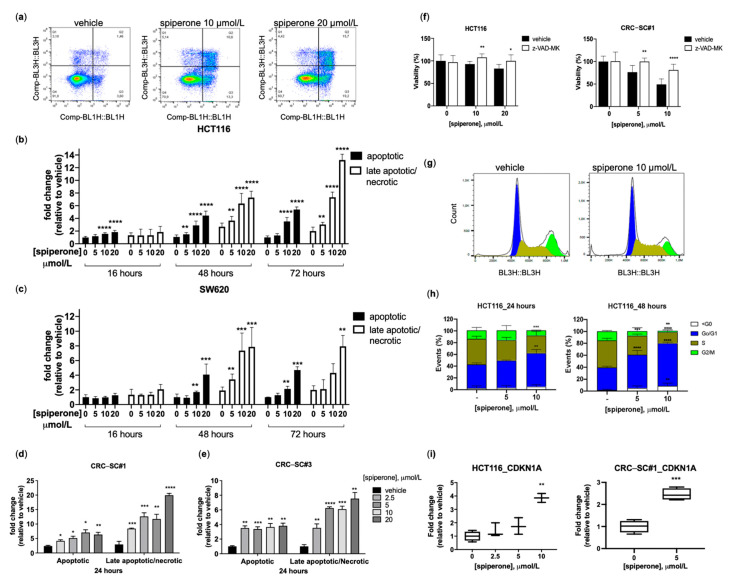
Spiperone induces apoptosis in CRC cells and induces cell cycle arrest in the G1 phase. Representative dot plots showing cell distribution of HCT116 cells treated for 48 h with different concentrations of spiperone after Annexin V/PI (Ax/PI) staining (**a**). Graph showing the analysis of HCT116 (**b**) and SW620 (**c**) cells treated with different concentrations of spiperone at different time points. Graphs showing the analysis of CRC-SC#1 (**d**) and CRC-SC#2 (**e**) cells treated with different concentrations of spiperone for 24 h. Cell populations are indicated as apoptotic (Ax+/PI−) and late apoptotic/necrotic (Ax+/PI+). Data represent the mean ± SD of at least three independent experiments performed in duplicate. *: Student’s *t*-test *p* < 0.05; **: Student’s *t*-test *p* < 0.01; ***: Student’s *t*-test *p* < 0.001; ****: Student’s *t*-test *p* < 0.0001. Cell viability was performed on HCTT16 and CRC-SC#1 cells co-treated for 24 h with different doses of spiperone along with vehicle or 10 μmol/L zVAD-FMK. Graphs displaying cell viability as the percentage of viable cells; data represent the mean ± SD of at least three independent experiments performed in triplicate. *: Student’s *t*-test *p* < 0.05; **: Student’s *t*-test *p* < 0.01; ****: Student’s *t*-test *p* < 0.0001 (**f**). Representative frequencies of distribution of PI staining were analyzed by flow cytometry of HCT116 cells treated with spiperone for 24 h (**g**). Numbers of cells in the G0, G1, S, and G2 phases of the cell cycle after 24 h and 48 h of treatment with scalar doses of spiperone (**h**); data represent the mean ± SD of at least three independent experiments performed in triplicate. **: Student’s *t*-test *p* < 0.01; ***: Student’s *t*-test *p* < 0.001; ****: Student’s *t*-test *p* < 0.0001. Gene expression analysis of CDKN1A by RT-qPCR. Relative expressions were determined by the ΔΔCt method and normalized with the control gene *GUSB* (**i**); data represent the mean ± SD of at least three independent experiments performed in duplicate. **: Student’s *t*-test *p* < 0.01; ***: Student’s *t*-test *p* < 0.001.

To assess the mechanism of cell death induced by spiperone, we investigated apoptosis by propidium iodide/Annexin V (PI/Ax) staining. A significant time- and dose-dependent increase in apoptosis was observed in both HCT116 and SW620 cells (Figure 2a–c). Similarly, a dose-dependent increase in the number of apoptotic cells was observed in CRC-SCs treated for 24 with the drug (Figure 2d,e). Cell death was significantly reverted by incubation of HCT116 cells and CRC-CSs with the caspase inhibitor Z-VAD-FMK (Figure 2f), suggesting that a caspase cascade is involved in apoptosis induced by spiperone.

Cell cycle analysis of HCT116 cells treated with spiperone showed a significant dose-dependent increase in the number of cells in the G1 phase, along with a decrease in the S/G2 phase, after both 24 and 48 h treatments (Figure 2g,h). G1 phase arrest was associated with increased expression of CDKN1A in both HCT116 and CRC-SC#1 cells treated for 24 h with spiperone (Figure 2i). Altogether, these data indicate that spiperone suppresses proliferation and induces apoptosis in CRC cells.

### 3.3. Spiperone-Treated Cells Maintain Lysosomal Membrane Integrity

**Figure 3:**
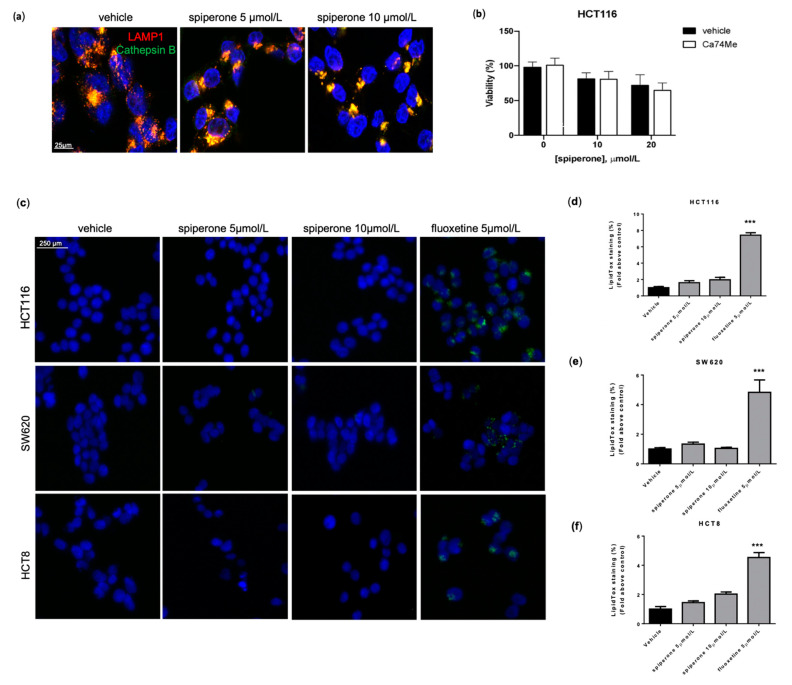
Treatment with spiperone does not induce lysosomal damage in CRC cells. Colocalization of cathepsin B and lysosomes was evaluated in HCT116 cells by using cathepsin B (green) and LAMP1 (red) antibodies after 16 h of treatment with spiperone. Nuclei were stained using DAPI. Pictures were acquired with a Leica SP8 confocal microscope (magnification: 63×) (**a**). Effect of the co-treatment with spiperone and the cathepsin B inhibitor Ca74Me at 10 μmol/L. After 30 min of pretreatment, HCT116 cells were treated with vehicle or 10 and 20 μmol/L spiperone for 24 h. Graph displaying cell viability as the percentage of viable cells. Data represent the mean ± SD of three independent experiments performed in triplicate (**b**). Accumulation of phospholipids was evaluated after 16 h of treatment with drugs using LipidTOX Green staining. Nuclei were stained using Hoechst 33342. Pictures were acquired by fluorescence microscopy (magnification: 20×). Representative images of cells treated with vehicle or 5 and 10 μmol/L spiperone (**c**). Histograms showing quantification of LipidTOX Green staining/blue nuclei staining ratio as the fold change relative to control in HCT116, SW620, and HCT8 cells. Data are presented as the mean ± SD from three independent experiments, each performed in triplicate. ***: Student’s *t*-test *p* < 0.001 (**d**–**f**).

We previously reported that the antineoplastic activity of several psychotropic drugs can be ascribed to their lysosomotropic properties [34]. In particular, although spiperone cannot be strictly classified as a cationic amphiphilic drug (CAD), we found that this drug induces lysosomal damage and cathepsin-mediated cell death in MCF7 cells [34]. To evaluate the role of lysosomes in CRC cell death induced by spiperone, we investigated lysosomal membrane integrity and lysosomal function via confocal microscopy. Although a significant enlargement of lysosomes was observed in treated HCT116 cells compared with controls, cathepsin B strictly colocalized with LAMP-1 (Figure 3a); moreover, treatment with the cathepsin inhibitor Ca74Me did not affect cell viability, excluding damage to lysosomal membranes and cathepsin-induced cell death following drug treatment (Figure 3b). LipidTOX Green staining was performed on three different CRC cell lines. Whereas treatment with 5 μmol/L fluoxetine—a well-recognized CAD used as a positive control—caused a significant accumulation of lysosomal phospholipids, treatment with 5 and 10 μmol/L spiperone induced only mild and non-significant increases in LipidTOX staining in CRC cell lines (Figure 3c–f). These data suggest that spiperone’s toxicity in CRC does not directly depend on the disruption of lysosomal function.

### 3.4. Spiperone Triggers Acute PLC-Dependent Ca^2+^ Modulation

**Figure 4:**
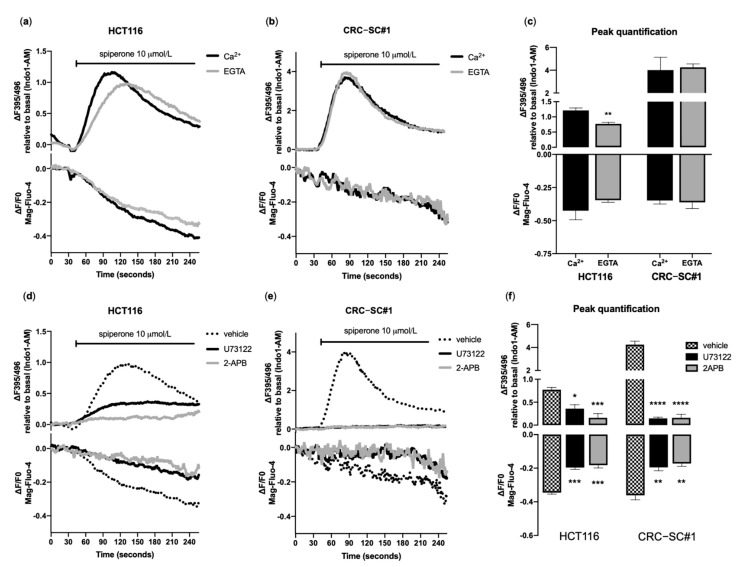
Spiperone increases intracellular Ca^2+^ concentration by inducing a PLC-dependent Ca^2+^ release from the ER. [Ca^2+^]_cyt_ (upper panel) and [Ca^2+^]_ER_ (lower panel) were simultaneously evaluated before and after spiperone exposure. Graphs representing the mean of fluorescence kinetics over time with (black line) or without extracellular Ca^2+^ (grey line) in HCT116 (**a**) and CRC-SC#1 (**b**) cells. Histogram displaying quantification of fluorescence peaks relative to basal signal for Indo-1 AM and Mag-Fluo-4 AM in HCT116 and CRC-SC#1 (**c**) cells. Evaluation of the effects of U73122 and 2-APB on spiperone-induced intracellular Ca^2+^ modulation, [Ca^2+^]_cyt_ (upper panel), and [Ca^2+^]_ER_ (lower panel) were simultaneously evaluated in the absence of extracellular Ca^2+^ before and after spiperone exposure. Graph representing the mean of fluorescence kinetics over time in cells pretreated with vehicle (dotted black line), U73122 10 μmol/L (solid black line), and 2APB 50 μmol/L (solid grey line) in HCT116 cells (**d**) and CRC-SC#1 cells (**e**). Histogram displaying quantification of fluorescence peaks relative to basal signal for Indo-1 AM and Mag-Fluo-4 AM in HCT116 cells and CRC-SC line #1 (**f**). Data represent the mean ± SD of at least three independent experiments. *: Student’s *t*-test *p* < 0.05; **: Student’s *t*-test *p* < 0.01; ***: Student’s *t*-test *p* < 0.001; ****: Student’s *t*-test *p* < 0.0001.

Spiperone was identified, through compound library screening, as being a potent enhancer of cytoplasmic Ca^2+^ ([Ca^2+^]_cyt_) in normal and cystic fibrosis airway epithelia [46]. To explore the possible role of Ca^2+^ in CRC cell death induced by spiperone, we measured free Ca^2+^ concentrations simultaneously in the ER ([Ca^2+^]_ER_) and in the cytosol by loading cells with the ratiometric fluorescent Ca^2+^ probe Indo-1 AM and the ER probe Mag-Fluo-4 AM, and analyzed Ca^2+^ kinetics via flow cytometry. Notably, in all tested cell lines, spiperone treatment evoked a large, single, slowly decaying spike of Ca^2+^, along with a significant decrease in [Ca^2+^]_ER_ (Figure 4a–c)—a kinetic of Ca^2+^ release that is quite different from that observed upon stimulation with physiological ligands such as histamine, which is characterized by a sharp, rapidly decaying peak associated with only a mild depletion of [Ca^2+^]_ER_ (Supplementary Figure S3a,b). The increase in [Ca^2+^]_cyt_ caused by the drug was observed in both the presence (KRB 2 mmol/L Ca^2+^) and absence (KRB 2 mmol/L EGTA) of extracellular Ca^2+^ (Figure 4a–c; Supplementary Figure S3c,d), suggesting that the acute increase in [Ca^2+^]_cyt_ induced by spiperone could in principle be the consequence of the release of Ca^2+^ from intracellular Ca^2+^ stores. Moreover, in nearly all of the tested cell lines, a higher response during spiperone exposure in the presence of extracellular Ca^2+^ was observed, suggesting a compensatory acute activation of the store-operated Ca^2+^ entry (SOCE) response (Figure 4a–c; Supplementary Figure S3c,d). In most non-excitable cells, the ER is the major source of intracellular Ca^2+^, which is released in response to extracellular stimuli through IP3 receptors (IP3Rs), which are activated by the second messenger inositol 1,4,5-trisphosphate (IP3) produced by phospholipase C (PLC) downstream membrane receptors [47,48,49]. In order to validate the hypothesis of ER origin of spiperone-evoked Ca^2+^, cells were pretreated for 30 min with 2-APB [50] or U73122 [51], and then stimulated with spiperone. Kinetic analysis showed a significant reduction in intracellular Ca^2+^ concentration ([Ca^2+^]_i_) mobilization in HCT116 and CRC-SC#1 cells pretreated with both 2-APB and U73122 (but not with U73433), compared with control cells (Figure 4d–f, Supplementary Figure S4a,b). Afterwards, we unmasked Ca^2+^ release from the ER by blocking SERCA pumps with thapsigargin (TG), and measured Ca^2+^ release from the ER. Spiperone was largely ineffective after treatment with TG and, similarly, the increase in [Ca^2+^]_cyt_ caused by TG was significantly abolished in cells pretreated with spiperone, compared to control cells (Supplementary Figure S5). Overall, these observations underpin the hypothesis that acute spiperone treatment primarily mobilizes Ca^2+^ from ER Ca^2+^ stores via activation of PLCs.

### 3.5. Spiperone Induces Ca^2+^/PLC-Dependent Cell Death

**Figure 5:**
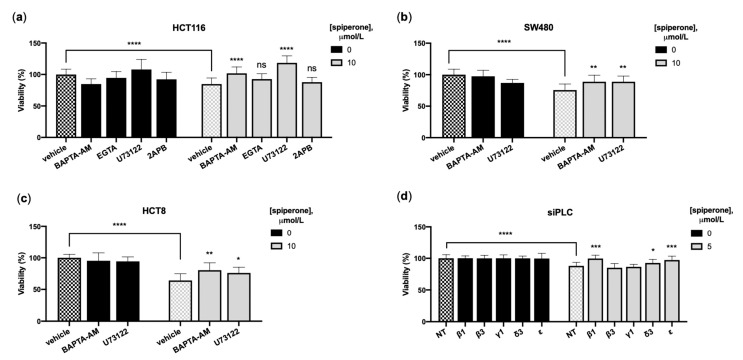
Spiperone induces Ca^2+^ and PLC-dependent cell death. Effect of co-treatment with spiperone and 1 μmol/L BAPTA-AM, 1 μmol/L EGTA, 2 μmol/L U73122, and 10 μmol/L 2APB in HCT116 cells. After 30 min of pretreatment, cells were co-treated with 10 μmol/L spiperone or vehicle for 24 h (**a**). Effect of co-treatment with spiperone and 1 μmol/L BAPTA-AM or 2 μmol/L U73122 in SW480 (**b**) and HCT8 cells (**c**). After 30 min of pretreatment, 10 μmol/L spiperone or vehicle were added, and co-treatment was maintained for 24 h. Effect of PLC silencing on spiperone-induced cell death. HCT116-silenced cells were treated for 48 with 5 μmol/L spiperone (**d**). Graphs displaying cell viability as the percentage of viable cells. Data show the mean ± SD of at least three independent experiments performed in triplicate. *: Student’s *t*-test *p* < 0.05; **: Student’s *t*-test *p* < 0.01; ***: Student’s *t*-test *p* < 0.001; ****: Student’s *t*-test *p* < 0.0001.

Spiperone Ca^2+^ signaling and the modulation of [Ca^2+^]_i_ levels play critical roles in several key processes that regulate cell survival or death in both normal and neoplastic cells [52,53,54]. Activation of Ca^2+^ channels resulting in transient and short but robust cytosolic Ca^2+^ peaks mediate a broad repertoire of cell physiological other hand, including proliferation, differentiation, migration, and secretion. On the contrary, long-lasting [Ca^2+^]_cyt_ increase and loss of [Ca^2+^]_cyt_ homeostasis could instead lead to cell death [55,56]. To define the role of [Ca^2+^]_i_ mobilization in spiperone-induced apoptosis, we performed viability rescue experiments with EGTA and BAPTA-AM. We observed a significant reduction in cell mortality in cells treated with BAPTA-AM (Supplementary Figure S6a,b), but not with the extracellular Ca^2+^ chelator EGTA (Figure 5a). These results, further confirmed in SW480 and HCT8 cells, indicate that intracellular Ca^2+^ dynamics are involved in spiperone-induced cell death (Figure 5a–c).

Since the ER is the primary source of intracellular Ca^2+^ mobilization, we examined the involvement of the PLC/IP3R pathway in spiperone-induced cell death by employing small molecules and gene silencing. Co-treatment with U73122 significantly improved cell viability of spiperone-treated cells but, unexpectedly, co-treatment with 2-APB was ineffective in reducing spiperone-induced cell death (Figure 5a–c). Since U73122 significantly rescued cell viability (Supplementary Figure S6c,d), we investigated which PLC isoform was involved in spiperone cytotoxicity by using small interfering RNA-mediated knockdown (siPLC) (Supplementary Figure S7a). Silencing of PLCꞵ1, 𝜀1, and ẟ3, but not of PLCꞵ3 or γ1, resulted in significant rescue of cell viability after spiperone treatment (Figure 5d). Silencing of PLCδ3, but not of the other investigated PLCs, was also associated with a significant reduction in acute [Ca^2+^]_cyt_ spike upon spiperone treatment (Supplementary Figure S7b,c).

**Figure 6:**
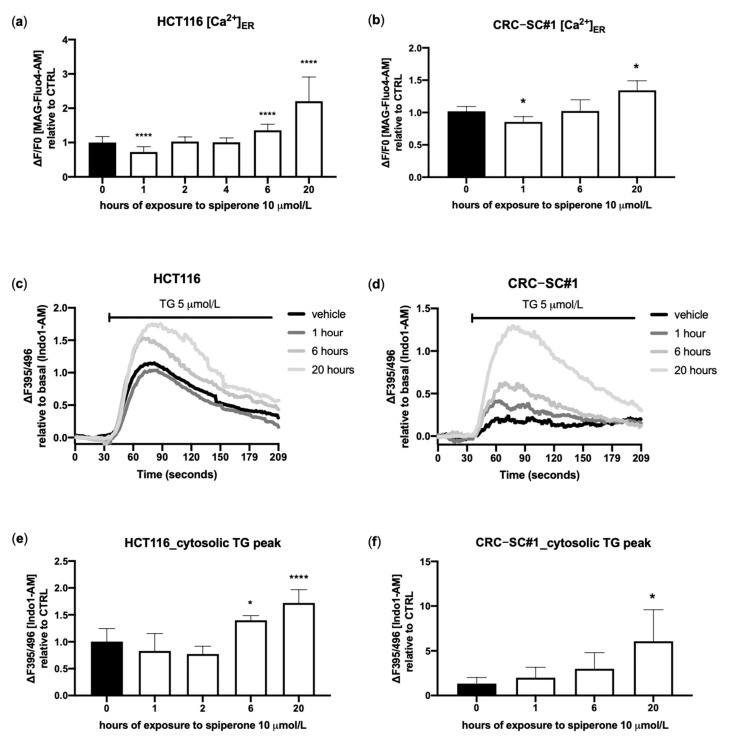
Spiperone induces a long-term increase in [Ca^2+^]_ER_ and enhances intracellular storage release. [Ca^2+^]_ER_ was evaluated in HCT116 and CRC-SC#1 cells treated with 10 µmol/L spiperone at different time points. Histogram displaying fluorescence quantification relative to control signal for Mag-Fluo-4 AM in HCT116 (**a**) and CRC-SC#1 (**b**) cells. [Ca^2+^]_cyt_ was monitored before and after 5 µmol/L TG exposure in cells treated with spiperone at different time points. Graph representing the mean of fluorescence kinetics over time in HCT116 cells (**c**) and CRC-SC#1 cells (**d**). Histogram displaying quantification of fluorescence peaks relative to the control signal for Indo-1 after 5 µmol/L TG exposure in HCT116 (**e**) and CRC-SC#1 (**f**) cells. Data show the mean ± SD of at least three independent experiments. *: Student’s *t*-test *p* < 0.05; ****: Student’s *t*-test *p* < 0.0001.

Since acute treatment with spiperone induced a slowly decaying spike of [Ca^2+^]_cyt_, associated with a strong depletion of [Ca^2+^]_ER_, we performed a deeper analysis of [Ca^2+^]_ER_ dynamics during long-term treatment in both HCT116 and CRC-SC#1 cells. A significant reduction in [Ca^2+^]_ER_ was still visible after 1 h of treatment, suggesting a potential prolonged [Ca^2+^]_ER_ depletion after subacute stimulation with spiperone. However, with longer treatment times, [Ca^2+^]_ER_ returned to baseline levels (2 h), and then rose significantly (6–8 h) to remain stably increased with respect to control after 20 h of treatment (Figure 6a,b). Spiperone-induced [Ca^2+^]_ER_ overload was significantly inhibited by BAPTA-AM (Supplementary Figure S8), whereas inhibition of SERCA pumps with TG revealed enhanced Ca^2+^ release from intracellular Ca^2+^ stores over time, as evidenced by a massive increase in [Ca^2+^]_cyt_ in spiperone-treated cells compared with controls after TG treatment (Figure 6c–f).

In light of the involvement of [Ca^2+^]_i_ disruption in spiperone cytotoxicity, we analyzed the role of the main calcium-regulated pathways that have been reported, under various physiopathological conditions, to transduce Ca^2+^-induced cell death and, in particular, the classical protein kinase C (cPKC) and Ca^2+^/calmodulin-dependent protein kinase II (CAMKII)/activated protein kinase (AMPK) pathways [57]. Activation of PKC, revealed by the increased phosphorylation of PKC substrates and phosphorylation of AMPK, was observed already after 5 min of stimulation with spiperone, and remained stable 60 min after treatment (Supplementary Figure S9a–c). Although both PKC and AMPK were activated upon spiperone treatment, neither the CAMKII inhibitor KN93, nor the calmodulin inhibitor W-13, nor the PKC inhibitor Go6850 improved cell viability (Supplementary Figure S10a–c).

Notably, treatment with both dorsomorphin and Go6850 enhanced spiperone-induced cell death, suggesting a possible protective role of AMPK and PKC against spiperone cytotoxicity (Supplementary Figure S10b,d). We also investigated calpains—a class of cysteine proteases activated by elevated [Ca^2+^]_cyt_ and capable of inducing proteolytic activation of caspases and execution of the apoptotic program [58,59]. Treatment with calpain inhibitor III showed no effect on cell viability (Supplementary Figure S12a).

In an attempt to delineate upstream signaling of intracellular Ca^2+^ dysregulation caused by spiperone, we pretreated HCT116 cells with a number of inhibitors of GPCR, small G proteins, and protein tyrosine kinases. Among the tested compounds, regorafenib caused a virtually complete ablation of acute Ca^2+^ release upon spiperone treatment, whereas gallein reduced the [Ca^2+^]_cyt_ enhancement by ~50%, but neither regorafenib nor gallein reversed spiperone’s cytotoxic effect. No changes in cell viability or in calcium-release kinetics were observed after pretreatment with CID-1067700, YM-254890, or genistein. Treatment with suramin resulted in a mild increase in cell viability in spiperone-treated cells, but also caused a significant boost in cell proliferation at the basal level (Supplementary Figure S11a–c). Analysis of signaling-activated downstream spiperone treatment showed the activation of ERK1/2 and P38 MAPK pathways in both HCT116 and CRC-SC#1 cells (Supplementary Figure S12b–e); therefore, we investigated their role in cell death by using specific inhibitors. The ineffectiveness of SB203580, SP600125, and U0126 in reducing cell death induced by spiperone allowed us to exclude a possible role of these MAPKs in the antitumor activity of the drug (Supplementary Figure S12f–h).

### 3.6. Spiperone Causes ER-Stress-Induced Cell Death

To deeply investigate the mechanism of action of spiperone in inducing CRC cell death, we analyzed the transcriptomic profile of HCT116 and CRC-SC#1 cells treated with 10 μmol/L spiperone for 20 h. The multivariate principal component analysis (PCA) plot confirmed that the biological replicates were intrinsically similar, while it displayed a significant separation between treated and control samples (Supplementary Figure S13a).

**Figure 7:**
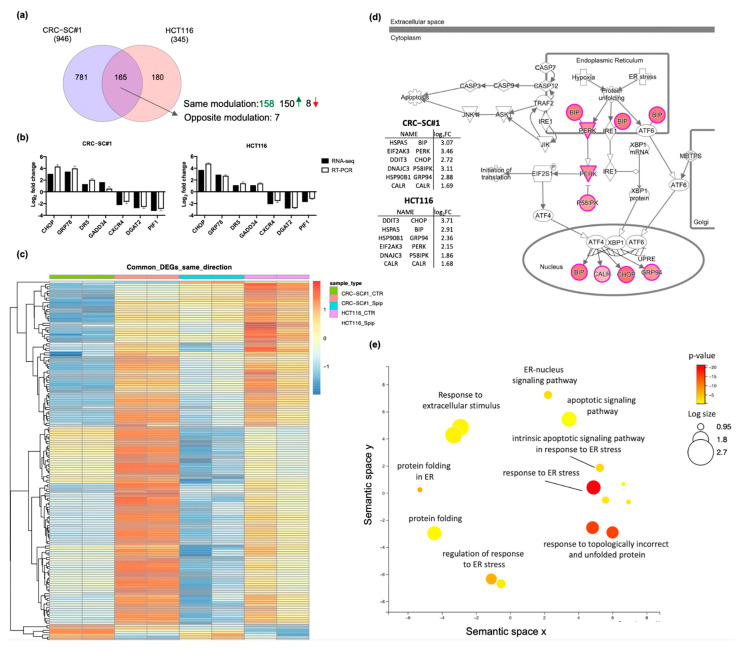
Spiperone induces ER stress in CRC cells. Venn diagram showing the number of DEGs between HCT116 and CRC-SC#1 cells (**a**). Log2FC validation of RNA-Seq analysis through RT-PCR in CRC-SC#1 and HCT116 cells (**b**). Heatmap showing unsupervised hierarchical clustering of the 158 common DEGs between the two comparison groups (HCT116-Spip vs. HCT116-CTR and CRC-SC#1-Spip vs. CRC-SC#1-CTR) that have the same trend (**c**). ER Stress molecular pathways from IPA Ingenuity software, and Log2FC of the upregulated genes. Red-colored molecules are upregulated in both of the two comparison groups and, therefore, upregulated after the treatment with spiperone in HCT116 and CRC-SC#1 cells (**d**). Semantic plot of the common enriched biological processes (**e**).

By using DESeq2 to infer the presence of differentially expressed genes (DEGs; Log_2_ FC > 1.5 and adjusted *p*-value < 0.05), a total of 1126 genes were found to be modulated by spiperone; of these genes, 781 were preferentially deregulated in CRC-SC#1 cells, 180 characterized HCT116 cells (Figure 7a), and 165 were common to both cell lines (Supplementary Table S1). Validation of RNA-Seq was performed, investigating seven common DEGs by RT-PCR, and data analysis revealed a good correlation between relative expression obtained by RT-PCR and RNA-Seq—Spearman’s rho (ρ) = 0.9437, *p* = 0.0003 in CRC-SC#1 cells, and ρ = 0.9781, *p* ≤ 0.0001 in HCT116 cells (Figure 7b).

For the identification of relevant biological pathways modulated by spiperone treatment, we performed Gene Ontology analysis of common DEGs with the same up- or downregulation pattern in both cell lines using ToppGene Suite and IPA Ingenuity software, as described in Section 2. A heatmap showing unsupervised hierarchical clustering of these 158 common genes with the same modulation is shown in Figure 7c. IPA Ingenuity analysis revealed a significant enrichment of genes belonging to the UPR (14.4%, *p*-value 4.13 × 10^−15^) and ER stress response (28.6%, 1.54 × 10^−9^). In particular, when we considered the individual genes, ER chaperone BiP (HSPA5/BIP), eukaryotic translation initiation factor 2 alpha kinase 3 (EIF2AK3/PERK), and DNA damage-inducible transcript 3 (DDIT3/CHOP) were the most upregulated genes observed in CRC-SC#1, with a Log2FC of 3.46, 3.11, and 3.07, respectively. Similarly, in HCT116 cells, DDIT3 and HSPA5 were the most upregulated, with a Log2FC of 3.71 and 2.91, respectively, while heat shock protein 90 beta family member 1 (HSP90B1/GRP94) ranked third, with a Log2FC of 2.36 (Figure 7d). The semantic plot obtained with ToppGene-derived GO terms associated with the 158 commonly modulated genes confirmed the upregulation of biological processes associated with ER stress and the UPR, along with apoptotic pathway activation (Figure 7e).

Notably, among the significantly downregulated genes in both HCT116 and CRC-SC#1 cells, we can mention the apoptosis inhibitor PIF1 5′-To-3′ DNA helicase (PIF1) [60] and the oncoprotein regulated by p53, PSRC1 (proline- and serine-rich coiled-coil 1/DDA3), required for normal progression through mitosis [61]. Moreover, both of the cell lines were characterized by lower expression of claudin-2 (CLDN2), C-X-C chemokine receptor type 4 (CXCR4), and cytochrome P450 family 24 subfamily A member 1 (CYP24A1), which codify for proteins commonly reported to be associated with cancer progression and invasiveness [62,63,64].

**Figure 8:**
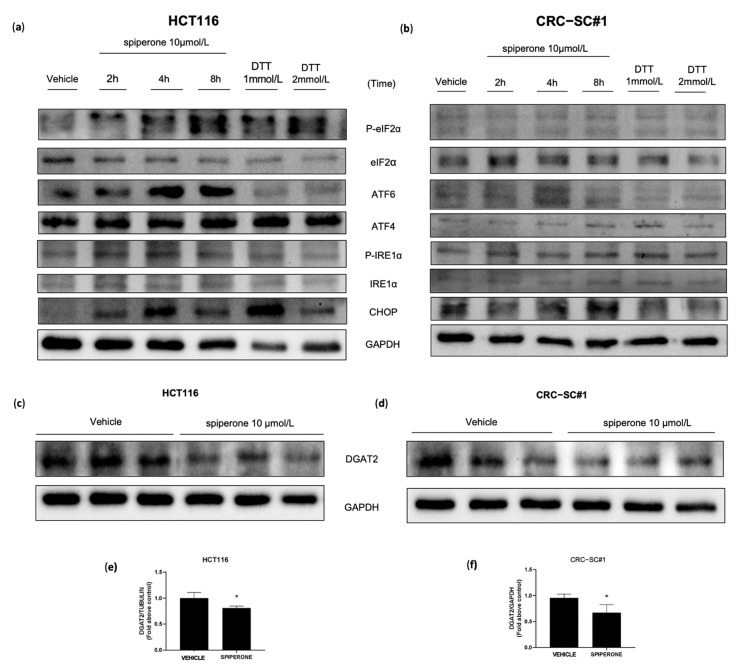
Spiperone induces ER stress in CRC cells. Western blot analysis of HCT116 cells (**a**) and CRC-SC#1 (**b**) after 2, 4 and 8 h of treatment with spiperone. Lysates were analyzed for P-eIF2α, eIF2α, ATF4 ATF6, P-IRE1α, IRE1α, CHOP, and GAPDH. Data are representative images of three independent experiments. Western blot analysis of DGAT2 protein in HCT116 (**c**) and CRC-SC#1 cells (**d**). After 20 h treatment with 10 μmol/L spiperone, lysates were analyzed for DGAT2 and GAPDH. Histograms displaying DGAT2 quantification in HCT116 cells (**e**) and CRC-SC#1 cells (**f**). Data are presented as the mean ± SD from three independent experiments. *: *p* < 0.05.

ER stress engages the UPR—a signaling network that enforces adaptive programs aiming to alleviate ER stress and promote cell survival. However, if ER homeostasis cannot be reestablished, the UPR results in cell death [65,66,67]. In order to validate RNA-Seq results, we investigated the activation of the three arms of the UPR—namely, PRKR-like endoplasmic reticulum kinase (PERK), activating transcription factor 6 (ATF6), and inositol-requiring enzyme 1 (IRE1)—by Western blot and RT-PCR analysis. UPR activation upon spiperone treatment was confirmed by Western blot analysis in both HCT116 and CRC-SC#1 cells, albeit with some slight differences. In HCT116 cells we observed strong phosphorylation of eIF2a already after 2 h of treatment, whereas the upregulation of ATF4 and ATF6 expression took place after 8 h and 4 h, respectively (Figure 8a; Supplementary Figure S14a). In CRC-SC#1 cells, the expression of ATF4 and ATF6 was observable after 8 and 4 h, respectively, while IRE1⍺ phosphorylation significantly increased after 2 h of treatment. At the same time, phosphorylation of eIF2a was negligible (Figure 8b; Supplementary Figure S14b). According to the increase in IRE phosphorylation, the conversion of unspliced XBP1 (uXBP1) to the spliced form (sXBP1) was already observed at the mRNA level by PCR analysis at 8 h after treatment, and was then verified by RNA-Seq, confirming the induction of the regulated IRE1-dependent decay (RIDD) (Supplementary Figure S13b–d). In agreement with XBP1 splicing, a marked downregulation of transmembrane protein family member 19 (TMEM19), basal cell adhesion molecule (BCAM), and diacylglycerol O-acyltransferase 2 (DGAT2) genes further confirmed RIDD activation in both cell lines (Supplementary Table S1) [68,69]. RT-PCR analysis further confirmed DGAT2 mRNA downregulation in HCT116 and CRC-SC#1 cell lines (Figure 7b).

**Figure 9:**
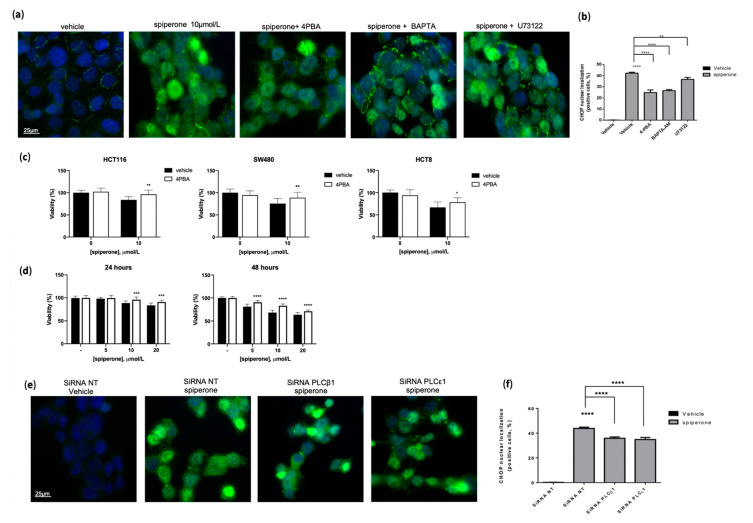
CHOP nuclear localization is mitigated by BAPTA-AM, U73122, 4-PBA, and PLC β1 and ε1 silencing. HCT116 cells were treated with 10 μmol/L vehicle or spiperone, alone or in combination with 10 μmol/L BAPTA-AM, 1 μmol/L U73122, and 10 μmol/L 4-PBA. CHOP nuclear localization was evaluated by fluorescence microscopy by using anti-CHOP primary antibody (green). Nuclei were stained with DAPI (blue) (**a**). Histogram showing the ratio of the number of cells presenting CHOP nuclear localization to the total number of cells (**b**). Data are presented as the mean ± SD of three independent experiments, each performed in triplicate. **: Student’s *t*-test *p* < 0.01; ****: Student’s *t*-test *p* < 0.0001. Effect of co-treatment with spiperone and the ER stress inhibitor 4PBA at 10 μmol/L. After 30 min of pretreatment, HCT116, SW480, and HCT8 cells were treated with 10 μmol/L vehicle or spiperone for 24 h (**c**). Graphs displaying cell viability as the percentage of viable cells. Data show the mean ± SD of at least three independent experiments performed in triplicate. *: Student’s *t*-test *p* < 0.05; **: Student’s *t*-test *p* < 0.01. Effect of CHOP silencing in HCT116 cells; HCT116-silenced cells were treated for 24 and 48 h with 5 μmol/L, 10 μmol/L, and 20 μmol/L spiperone (**d**). Graphs displaying cell viability as the percentage of viable cells; data show the mean ± SD of at least three independent experiments performed in triplicate. ***: Student’s *t*-test *p* < 0.001; ****: Student’s *t*-test *p* < 0.0001. Effect of PLC ꞵ1 and PLC 𝜀1 silencing on CHOP nuclear localization. HCT116-silenced cells were treated for 24 h with 10 μmol/L spiperone (**e**). Graph showing the ratio of the number of cells presenting CHOP nuclear localization to the total number of cells (**f**); data are presented as the mean ± standard deviation from three independent experiments, each performed in triplicate. ****: Student’s *t*-test *p* < 0.0001.

When the primary cause of ER stress is prolonged or excessive, the UPR switches its signaling toward the activation of cell death—typically by apoptosis [70,71,72,73]. To ascertain the role of ER stress and the UPR in spiperone-triggered cell death, we investigated protein levels and nuclear localization of DDIT3/CHOP—a major player in cell death induction downstream UPR [74] (Figure 9a,b). Immunofluorescence analysis displayed a significant increase in the nuclear localization of CHOP in cells treated with 10 μmol/L spiperone for 16 h, highlighting an increase not only in CHOP expression, but also in its transcriptional activity (Figure 9a,b). The co-treatment of CRC cell lines with the chemical chaperone 4-phenyl butyric acid (4-PBA)—a compound known to alleviate ER stress—reduced CHOP nuclear localization, and significantly rescued spiperone-induced cell death, suggesting that ER stress and CHOP activity are directly involved in spiperone’s cytotoxicity (Figure 9a–c). Interestingly, a bioinformatic analysis demonstrated that a subset of 48 genes out of the original 158 DEGs could potentially be a direct target of CHOP/DDIT3 (Supplementary Figure S15), supporting the fact that CHOP’s nuclear localization is associated with its increased transcriptional activity. The role of CHOP in spiperone-induced apoptosis was therefore validated by using siRNA-mediated knockdown of CHOP. Western blot analysis confirmed the abrogation of CHOP induction by spiperone in siRNA-transfected cells (Supplementary Figure S14d), whereas viability assays showed a significant increase in cell viability in CHOP-silenced cells after 24 h and 48 h of treatment with the drug (Figure 9d). These results were further confirmed in SW480 cells (Supplementary Figure S14c).

We previously demonstrated that Ca^2+^ and PLCs are involved in spiperone-induced cell death. To evaluate their role in UPR induction, we analyzed CHOP nuclear localization in HCT116 cells treated with spiperone, in the presence or absence of BAPTA-AM or U73122. Results showed a strong reduction in CHOP nuclear localization with BAPTA-AM, whereas a weaker but still significant reduction was observed in cells treated with U73122 (Figure 9e,f) and in PLCꞵ1- and PLC𝜀1-silenced cells.

Both ER and mitochondrial stress are strong inducers of autophagy [75,76,77]. Moreover, autophagy is a form of type II programmed cell death frequently induced by increased [Ca^2+^]_cyt_ and activation of the CAMMK2/AMPK pathway [78]. Hence, we checked whether autophagy played any role in spiperone-induced CRC cell death by investigating LC3 turnover (LC3B-I to LC3B-II conversion). Western blot analysis showed an increase in the autophagic flux in HCT116 cells treated with 5 and 10 μmol/L spiperone and 50 μmol/L chloroquine for 16 h (Supplementary Figure S16a,b). Accordingly, reduced phosphorylation of P70S6K T389 and S6 ribosomal protein S235/236 was suggestive of mTOR pathway downregulation (Supplementary Figure S16c–e). Although autophagy was activated upon spiperone treatment, its pharmacological inhibition with 3-methyladenine (3-MA) was ineffective in reducing spiperone-induced cell death (Supplementary Figure S16f).

### 3.7. Spiperone Induces Mitochondrial Damage

**Figure 10:**
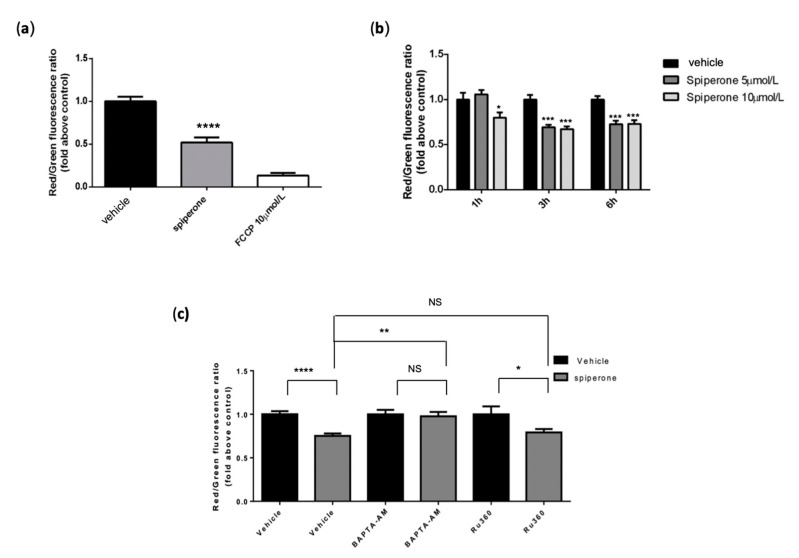
Spiperone induces mitochondrial damage. Mitochondrial membrane potential depolarization was evaluated by JC-1 staining after 1, 3, 6 (**b**), and 16 h (**a**) treatment with 5 and 10 μmol/L spiperone, alone or in combination with 10 μmol/L BAPTA-AM and 10 μmol/L of the MCU inhibitor Ru360 in HCT116 cells (**c**). Pictures were acquired via fluorescence microscopy. Histogram showing quantification of the red/green fluorescence ratio as fold change relative to controls. Data are presented as the mean ± SD from three independent experiments, each performed in triplicate. *: Student’s *t*-test *p* < 0.05; **: Student’s *t*-test *p* < 0.01; ***: Student’s *t*-test *p* < 0.001; ****: Student’s *t*-test *p* < 0.0001.

Several regulatory components link UPR with mitochondrial regulation and function [79,80]. Therefore, we investigated whether mitochondrial damage could contribute to spiperone cytotoxicity. For this purpose, we treated cells with spiperone for 1, 3, 6, and 16 h, and evaluated mitochondrial membrane depolarization by JC-1 staining. A reduction of nearly 30% in the red/green fluorescence ratio in spiperone-treated cells, compared to controls, was observed already after 3 h of spiperone treatment, whereas a reduction of ~50% was evident after 16 h (Figure 10a,b). Then, we performed oxygen consumption rate (OCR) experiments in HCT116 cells after 1 and 6 h of treatment with 10 μmol/L spiperone. OCR analysis showed an overall increase in mitochondrial respiratory chain activity, although not associated with an increase in ATP production, after 1 h of spiperone treatment (Supplementary Figure S17). On the other hand, a moderate but significant decrease in cellular respiration and a reduction in ATP production were assessed after 6 h of treatment.

To investigate the hypothesis that mitochondrial depolarization could be induced by mitochondrial Ca^2+^ uptake, we performed JC-1 staining in cells treated with spiperone alone, or in combination with BAPTA-AM or the mitochondrial calcium uniporter (MCU) inhibitor RU360. BAPTA-AM significantly rescued spiperone-induced mitochondrial depolarization, whereas treatment with RU360 was ineffective in reducing depolarization (Figure 10c), suggesting that mitochondrial dysfunction is caused by intracellular [Ca^2+^] dysregulation, but not by mitochondrial Ca^2+^ overload.

### 3.8. Spiperone Treatment Disrupts Lipid Metabolism in CRC Cells

We demonstrated by RNA-Seq and Western blot that DGAT2 mRNA—a preferential target of RIDD downstream ER stress—is significantly downregulated in cells treated with spiperone (Figure 7b and Figure 8c–f). DGAT1 and DGAT2 catalyze the final step of triacylglycerol (TAG) synthesis, thus playing a key role in controlling lipid biosynthesis [81], but additional acyltransferase activities have been probed for both DGAT1 and DGAT2 enzymes. Researchers have recently unveiled a crosstalk between glycerolipid and sphingolipid metabolism through DGAT2-dependent synthesis of 1-O-acylceramide from ceramide and fatty acyl-CoA in the ER–lipid droplet interface (LD) [82]. This non-canonical acyltransferase activity was reported to favor tumor cell survival and lead to chemoresistance in CRC cells due to the sequestration of pro-apoptotic ceramide via 1-O-acylceramide formation [82]. To explore the potential role of lipid dysregulation as a consequence of ER stress and DGAT2 downregulation, we investigated the lipidomic profile of CRC cells treated with spiperone (HPLC-MS/MS) (Figure 8c–f).

Lipidomic analysis was performed with an untargeted approach on HCT116 and CRC-SC#1 cells after 20 and 40 h of drug treatment. Unsupervised multivariate PCA and partial least squares discriminant analysis (PLS-DA) showed significant separation between treated and control samples, both for HCT116 and CRC-SC#1 cells (Supplementary Figure S18a–d). From the data processing of mass spectrometry results, 30 lipid subclasses (defined by head group) and 683 species (defined by the head group, fatty acid tail length, and saturation) were identified across all samples (Supplementary Table S2). By analyzing normalized areas, the average quantity of the major lipid classes was found to be similar in HCT116 and CRC-SC#1 cells (Supplementary Figure S19a–d). The class most represented was glycerophospholipids (GPLs, 86% and 72% in HCT116 and CRC-CS#1, respectively), followed by glycerolipids (GL, 7.8 and 25%), sphingolipids (SL, 5.6 and 2.8%), and fatty acids (FA; 0.56 and 0.21%). Although the total relative abundance of the main lipid classes—such as glycerophospholipids, glycerolipids, sphingolipids, and fatty acids—was similar in treated and untreated cells, as shown in Supplementary Figure S19, when the single lipid subclasses belonging to each class were evaluated, significant differences were observed between treated and untreated cells.

**Figure 11:**
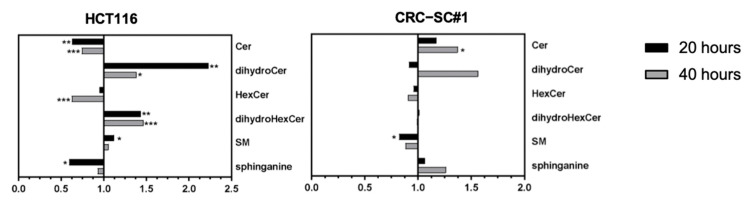
Analysis of sphingolipids for HCT116 and CRC-SC#1 cell lines. Graphs displaying fold change for HCT116 cells (**left panel**) and CRC-SC#1 cells (**right panel**) relative to ceramides (Cer), hexosylceramides (HexCer), sphingomyelins (SM), sphinganine, dihydroCer, and dihydroHexCer species merging 20 and 40 h treatment. *: Student’s *t*-test *p* < 0.1; **: Student’s *t*-test *p* < 0.01; ***: Student’s *t*-test *p* < 0.001.

In particular, among the sphingolipids (SL), all subclasses demonstrated remarkable alterations in both HCT116 and CRC-SC#1 cells upon spiperone treatment (Figure 11; Supplementary Figure S19e). In HCT116 cells, a significant overall reduction in ceramides (Cer) and hexosylceramides (HexCer) was observed in association with significantly increased levels of dihydroceramides (dihydroCer), dihydrohexosylceramides (dihydroHexCer), and sphingomyelins (SM). In CRC-CS#1 cells, on the other hand, spiperone treatment induced a significant enrichment in Cer and a reduction in SM, whereas an increase in dihydroCer was observed after 40 h of treatment. Alterations in SM and Cer expression were also observed in both HCT116 and CRC-SC#1 cells by using the VIP score plot, which ranks the variables according to their importance in the projection used by the PLS model (Supplementary Figure S18b,d).

**Figure 12:**
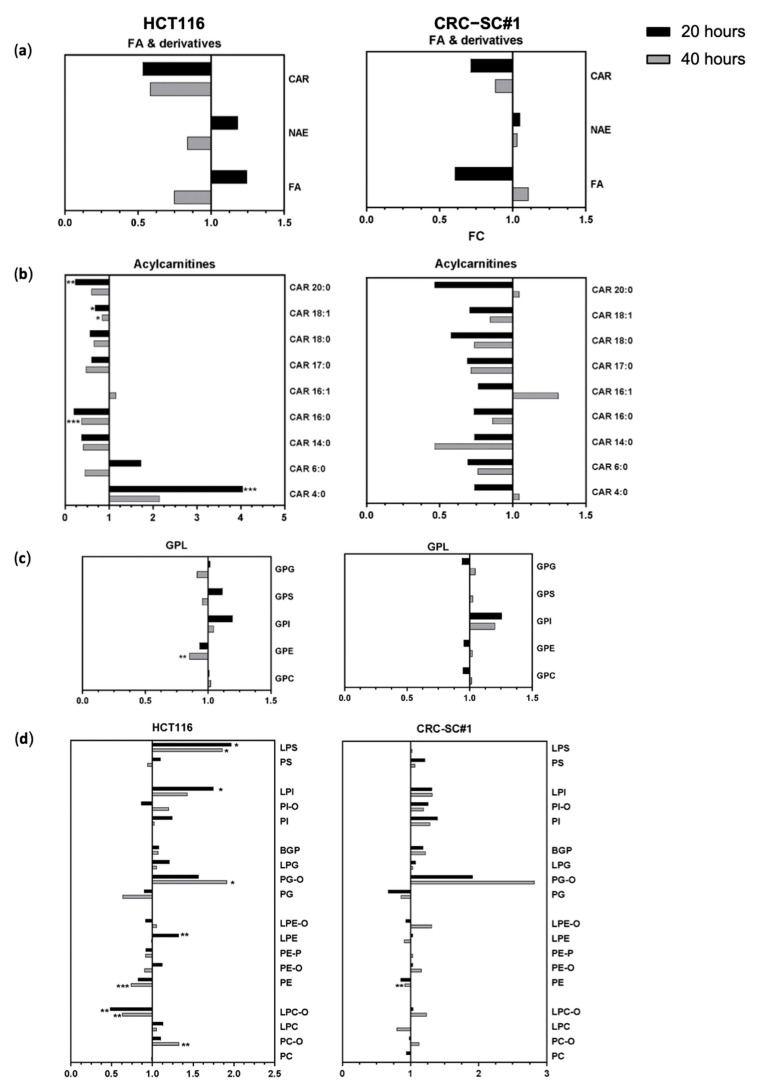
Lipidomic results for HCT116 and CRC-SC#1 cells. Graphs displaying the fold change for HCT116 cells (left panels) and CRC-SC#1 cells (right panels). The fatty acid derivatives are reported with the subclasses identified: acylcarnitine (CAR), N-acyl ethanolamines (NAE), and free fatty acids (FAs) (**a**). CAR characterization (**b**). Glycerophospholipids (GPLs) reported with the subclasses identified: glycerophosphoglycerol (GPG), glycerophosphoinositol (GPS), glycerophosphoinositols (GPIs), glycerophosphoethanolamine (GPE), and glycerophosphocholine (GPC) (**c**). For each class, the identified derivatives were reported considering both the lyso (LPS, LPI, LPG, LPE, LPC) and ether forms (PI-O, PG-O, PE-O, PE-P, PC-O) (**d**). *: Student’s *t*-test *p* < 0.1; **: Student’s *t*-test *p* < 0.01; ***: Student’s *t*-test *p* < 0.001.

Among FAs, an overall reduction in acylcarnitines (CAR) was observed in both HCT116 and CRC-SC#1 cells, with the exception of CAR4:0, which was significantly enriched in HCT116 cells (Figure 12a,b). Among GPLs, a significant reduction in phosphatidylethanolamines (PEs) and increased levels of glycerophosphoinositols (GPIs) and ether-linked phosphatidylglycerols (PG-Os) were observed in both HCT116 and CRC-CS#1 cells after both 20 and 40 h of spiperone treatment (Figure 12c). A change in the abundance of lysophospholipids (LPLs) was associated with spiperone treatment in HCT116 cells, and included a significantly higher proportion of lysophosphatidylinositols (LPIs), lysophosphatidylserines (LPSs), and lysophosphatidylethanolamines (LPEs), but a significant reduction in lysophosphatidylcholines (LPCs) (Figure 12d). No major differences were found in GL distribution (Supplementary Figure S19c,d).

Next, we analyzed the distribution and enrichment of the single lipid species according to cell type and treatment. To identify differentially expressed species, we screened and selected species with more than 1.5-fold change (FC) and *p*-values < 0.05 in at least one of the two treatment time points, and then we compared the observed number of species for each class with significant variation with that expected by chance (Supplementary Table S3). A total of 250 and 41 lipid species showed significant differences in the treated HCT116 and CRC-SC#1 cells, respectively, as compared with untreated cells (Supplementary Table S2). Among a total of 130 significantly increased lipid species characterizing spiperone-treated HCT116 cells, PE P-36:0|PE P-18:0_18:0, PI 32:2, and SM 38:0;2O|SM 17:0;2O/21:0 represented the species with the highest FC. A significant enrichment for SL (34/95; *p* < 0.001)—in particular dihydroCer (5/8; *p* = 0.009), dihydroHexCer (6/8; *p* = 0.001) and SM (20/43; *p* < 0.001) and for LPI (4/6; *p* = 0.0149)—was also observed. Among the 120 significantly reduced species, TG 60:1|TG 18:0_24:0_18:1, PC 41:1, and LPE 24:0 were the most downregulated lipid species, whereas phosphatidylcholines (PC, 26/88; *p* = 0.0023), phosphatidylethanolamines (PE, 23/47; *p* > 0.001), and ceramides (8/16; *p* = 0.0016) were the classes with the highest numbers of deregulated species. When we analyzed CRC-CS#1 cells, out of a total of 29 upregulated species upon spiperone treatment, LPI (3/6; *p* = 0.0017), phosphatidylinositols (PI, 5/26; *p* = 0.028), and ceramides (5/16; *p* < 0.001) were significantly enriched in CRC-SC#1 cells, with PI 38:4|PI 18:1_20:3, Cer 42:3;2O|Cer 18:2;2O/24:1, and LPI 16:1 showing the most remarkable changes. Among the 12 significantly reduced species, 5 were represented by dihydroHexCer (*p* < 0.001), with Hex3Cer 42:2;2O|Hex3Cer 18:1;2O/24:1 representing the SL species with the highest variation.

### 3.9. Spiperone Induces Golgi Apparatus Deregulation

**Figure 13:**
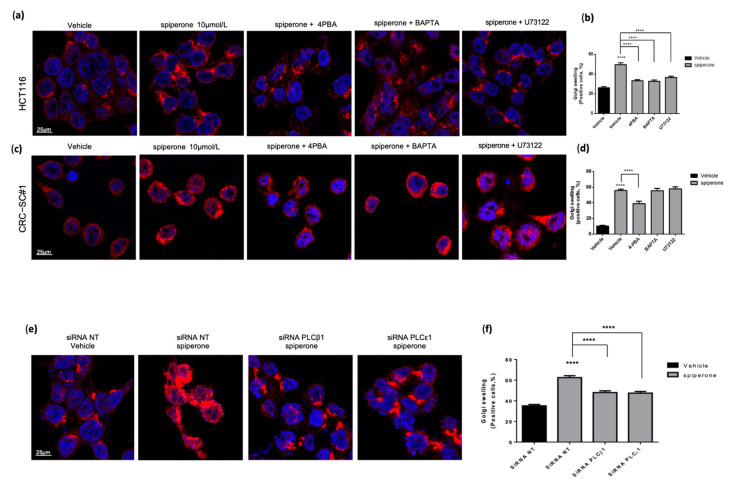
Spiperone induces swelling of the Golgi apparatus (GA). The effect of co-treatment with spiperone and 10 μmol/L BAPTA-AM, 1 μmol/L U73122, and 10 μmol/L 4-PBA on GA morphology was evaluated via confocal microscopy in HCT116 cells and CRC-SC#1 cells. The GA was stained using anti-GOLGIN 97 primary antibody and Alexa Fluor-546 secondary antibody; nuclei were stained using DAPI (**a**,**b**). Histogram showing the ratio of the number of cells presenting GA swelling to the total number of cells in HCT116 cells (**c**) and CRC-SC#1 cells (**d**). Representative images of the effects of spiperone treatment on GA morphology in HCT116 cells silenced for PLCβ1 and PLCε1 (**e**). Histogram showing the ratio of the number of cells presenting GA swelling to the total number of cells (**f**). Data are presented as the mean ± SD from three independent experiments performed in triplicate. ****: Student’s *t*-test *p* < 0.0001.

A prolonged dysregulation of lipid metabolism and turnover results in intracellular GPL, Cer, and SM imbalance in cell membranes and the Golgi apparatus (GA), progressively leading to cell death [83,84]. Therefore, we investigated the effects of spiperone treatment on GA morphology via immunofluorescence staining using GOLGIN97—a structural protein of trans-Golgi. Our results showed significant swelling of the GA in both HCT116 and CRC-SC#1 cells after spiperone treatment (Figure 13a–d). Co-treatment with the chemical chaperone 4-PBA was able to reverse GA swelling in both cell lines, highlighting the association between ER stress induced by spiperone and GA impairment (Figure 13a–d). Since we previously demonstrated that inhibition of PLC and Ca^2+^ signaling was able to reduce ER stress induced by spiperone, we treated our cells with spiperone in the presence or absence of BAPTA-AM, U73122, and PLCꞵ1 and PLC𝜀1 silencing (Figure 13a–f). Results showed a consistent reversal of GA swelling in HCT116 cells, but not in CRC-SC#1 cells, suggesting that spiperone induces GA damage through different mechanisms in the two cell lines.

## 4. Discussion

CRC represents the third most diagnosed malignancy and the second leading cause of cancer death in the world [1]. More than 50% of CRC patients develop chemoresistant metastasis and, despite improvements in cytotoxic and targeted therapy, metastatic disease is still incurable, with a survival rate of more than 5 years in only 20% of cases [85]. This has raised concerns over the progress of CRC therapy, and implies that alternative conceptual and practical approaches are required for the treatment of advanced-stage CRC [14].

Drug repurposing might represent a valid therapeutic option, especially in frail patients who are no longer candidates for aggressive therapeutic approaches [86,87]. The antineoplastic activity of psychiatric medications is supported by both epidemiological and preclinical evidence. Although individuals with schizophrenia are exposed to more environmental noxious agents that contribute to tumor development (e.g., tobacco and alcohol) than the general public, population-based studies show that patients with schizophrenia who are receiving psychotropic drugs have a lower cancer incidence than the general population, suggesting that psychiatric medications may display positive effects on certain human cancers [88,89,90,91]. In particular, a lower incidence than expected was observed for rectal cancer in male patients with schizophrenia [90], colon cancer in female neuroleptic users [92], and CRC in antidepressant users [93].

In this study, we identified spiperone—a typical antipsychotic drug belonging to the butyrophenone family, approved in Japan in 1969 for the treatment of schizophrenia [33]—as a promising compound for CRC therapy. Viability assays demonstrated a potent cytotoxic activity of spiperone against several CRC cell lines at lower micromolar concentrations, compatible with therapeutic concentrations in humans [94], whereas the viability of normal, non-cancerous cells was only slightly affected by very high doses of the drug. Moreover, spiperone caused a reduction in the clonogenic potential and induced cell death of CRC cells with stem-like features grown in colonospheres. These data suggest that spiperone can target specific drivers of cancer not only in differentiated CRC cells, but also in CRC-SCs, which represent the main cause of tumor growth, metastasis formation, and relapse [17,95].

Drug repurposing in cancer therapy takes advantage of the fact that some of the unintended off-targets of a compound might correspond to known anticancer targets, while others may reveal new cancer vulnerabilities [96,97]. With the aim of clarifying the mechanism of action of spiperone in CRC, we investigated already known and novel signaling pathways potentially affected by this drug. We first demonstrated that spiperone induces a significant [Ca^2+^]_i_ mobilization, confirming reported pieces of evidence in embryonic kidney 293 (HEK293) cells [98]. In agreement with a previous work [46], the characterization of spiperone-induced acute Ca^2+^ movements in CRC cells revealed a PLC/IP3R-dependent increase in [Ca^2+^]_cyt_ associated with a sustained depletion of [Ca^2+^]_ER._

In cancer cells, [Ca^2+^]_i_ signaling has substantial effects on a variety of cellular processes, ranging from cell survival and proliferation to cell death [52,53,54]. It is well recognized that cancer cells display disrupted Ca^2+^ signaling, where the activity of Ca^2+^-regulating proteins and Ca^2+^ pumps/channels is altered [99]. Hence, remodeling of these derailed Ca^2+^ features could represent a potential target for cancer therapies. Several drugs—such as cisplatin, As_2_O_3_, and topotecan—have been reported to induce apoptosis through the modulation of Ca^2+^-signaling-dependent mechanisms [100].

To elucidate the role of [Ca^2+^]_i_ mobilization in spiperone-induced cell death, we performed viability rescue experiments with Ca^2+^-chelating agents. The significant reduction in HCT116 cells’ mortality observed after treatment with the intracellular Ca^2+^ chelator BAPTA-AM, but not with the extracellular Ca^2+^-chelating agent EGTA, indicates that intracellular Ca^2+^ dynamics alone are involved in spiperone-induced cell death. Viability rescue experiments performed after treatment with U73122 and silencing of PLC genes substantiated the involvement of PLCs in spiperone-induced cell death but, unexpectedly, no change in cell viability was observed after inhibition of IP3R with 2APB, nor after inhibition of PKC, CAMKII, calmodulin, and calpain. These observations suggest that acute, IP3-dependent [Ca^2+^]_i_ mobilization observed upon spiperone treatment is a side effect not associated with cell death, and that the antineoplastic activity of the drug is possibly mediated by a PLC-dependent DAG pathway and/or phosphoinositide imbalance affecting intracellular Ca^2+^ mobilization. Hence, the protective effect of BAPTA against spiperone-induced cell death is likely due to its chronic impact on [Ca^2+^]_i_ and downregulation of Ca^2+^-dependent metabolism responsible for spiperone’s cytotoxicity.

Among the PLC isozymes expressed in our cell line models, we identified PLCβ1, -ε1, and -δ3 as being involved in spiperone-induced cell death, but neither Gαq nor Gβγ inhibition were effective in reducing spiperone’s cytotoxicity. PLCβ is a well-known PLC isozyme whose activation is typically downstream of G-protein-coupled receptors. While all PLCβ isoforms are effectors of Gαq, only β2 and β3—and to a lesser extent β1 [101]—can be activated by Gβγ subunits [49]. However, it is now well established that PLCβ1 is the most abundant isoform in the nucleus, where it sustains nuclear phosphoinositide signaling by hydrolyzing PtdIns(4)P and PtdIns(4,5)P2 [48,102]. Nuclear PLCβ1 has been reported to modulate cell cycle progression—particularly G1/S transition—and to interact with several proteins involved in cellular differentiation, nuclear import, mRNA processing, and apoptosis [103]. Today, the mechanism of PLCβ1 activation in the nucleus is still controversial, since evidence of nuclear localization of Gαq is still lacking [102,104]. The contribution of PLCβ1 in spiperone-induced cell death through a Gαβγ-independent mechanism supports a possible involvement of nuclear PLCβ1. Notably, recent studies in myelodysplastic syndromes reported PLCβ1 mRNA as being correlated with favorable clinical outcomes, whereas decreased PLCβ1 expression was associated with poor prognosis [105,106]. However, further studies are needed in order to characterize nuclear PLCβ1 activity in neoplastic cells, and its potential role in CRC cell death.

PLCε is the largest member of the PLC enzyme family, with unique features enabling it to integrate signals from both GPCRs and RTKs [107]. Activation occurs via the small GTPases of the RAS, RAP, and RHO families, as well as βγ subunits [108], and mediates sustained signaling compared to activation by PLCβ enzymes [109,110]. The contribution of PLCε in cancer remains controversial, and it can switch roles from tumor suppressor to oncogene depending on the type of cancer [111,112,113]. A number of studies have found evidence to support the tumor-suppressive role of PLCε in CRC development, where it can function as a Ras receptor and induce apoptosis [113,114]. Moreover, it was demonstrated that PLCε expression is usually downregulated in RAS-driven cancers, such as colon cancer, and that its overexpression was associated with reduction in the proliferation of skin cancer cells in vivo [115]. However, despite the evidence of a tumor-suppressive role in CRC, the molecular mechanisms behind PLCε activity require further investigation, as does its contribution to spiperone-induced cell death.

Our results showed that PLCδ3 is required for both acute spiperone-induced Ca^2+^ mobilization and spiperone cell toxicity. PLCδ is usually activated by physiological concentrations of Ca^2+^ (10 nmol/L to 10 μmol/L), which are achieved in cells by activation of other PLCs, or by extracellular Ca^2+^ entry through Ca^2+^ channels [48,116,117,118,119]. Therefore, PLCδ family members’ activity might be secondarily enhanced by intracellular Ca^2+^ mobilization to amplify PLC activity and potentiate Ca^2+^ signaling. The PLCδ family has been reported to function as a potential tumor suppressor in several cancers by inducing G1 cell cycle arrest and inhibiting the β-catenin pathway [105,120,121,122]. In particular, in CRC, PLCδ was shown to inhibit anchorage-independent growth through the induction of E-cadherin expression and through the attenuation of KRAS/MEK/ERK signaling [123]. However, despite evidence of the oncosuppressor function of PLCδ1, there is still little knowledge about the activity of PLCδ3, whose activation in cancer cells is still poorly defined and controversial [124].

PLCs can be activated downstream of RTKs, GPCRs, and small GTPases [49,125]. In an attempt to elucidate the signaling pathway upstream of Ca^2+^-induced cell death, we investigated inhibitors of heterotrimeric G proteins and protein tyrosine kinases for their capacity to reverse spiperone cytotoxicity. Regorafenib and gallein caused a significant reduction in acute intracellular Ca^2+^ release upon spiperone treatment, but were unable to reverse the cytotoxic effect of the drug, similar to bona fide inhibitors of Gaq, Gas, or RAS-superfamily GTPases. Currently, known pharmacological targets of spiperone include serotonin (5-HT1A, 5-HT2A) and dopamine (D2, D3, D4) receptors [126,127]; however, we previously demonstrated that spiperone toxicity was not associated with the conventional pharmacological properties and clinical use of this compound [34]. The concept of polypharmacology, which involves the interaction of drug molecules with multiple targets, is well known among neuroleptics. These drugs, indeed, have been demonstrated to recognize multiple aminergic GPCR [128] ion channels, such as N-methyl-D-aspartate receptor (NMDAR) sigma receptors [129]. Glycine transporter and glycogen synthase kinase 3 (GSK3) [130] were also reported as potential targets for antipsychotic drugs.

By hydrolyzing phosphatidylinositol 4,5-bisphosphate (PI(4,5)P2) into DAG and IP3, PLC activation not only triggers the release of Ca^2+^ from intracellular stores, but is also a modulator of phosphoinositide balance [49]. PI(4,5)P2 directly regulates a variety of cellular functions, including cytokinesis, cytoskeletal remodeling, membrane dynamics, phagocytosis, and channel activity. It is now well known that several membrane and intracellular ion channels are regulated by phosphoinositides [131]. In particular, PI(4,5)P2 depletion after PLC activation was reported to considerably alter the function of voltage-gated potassium and Ca^2+^ channels [132,133] and Ca^2+^-activated chloride channels [134] in the plasma membrane.

Transient receptor potential (TRP) channels are a class of cation-permeable channels reported to be finely regulated by both phosphoinositides and DAG [135]. In particular, TRPCs are activated downstream of PLC, whereas other TRP channels—such as TRPM7 [136], TRPM4 [137], and TRPV5 [138]—are activated by PI(4,5)P2 and, therefore, can be negatively modulated by PLC activation. Considering the tight link between TRP, PI(4,5)P2, and [Ca^2+^]_i_, it is conceivable that spiperone activity could be associated with TRP modulation. Since our data revealed that extracellular Ca^2+^ is likely not implicated in spiperone-induced cell death, we can speculate as to the involvement of intracellular TRP channels. For example, TRPVs were reported to localize on the ER and Golgi membrane and regulate intracellular Ca^2+^ homeostasis [139]. In particular, TRPV1 was associated with the release of Ca^2+^ from internal stores, followed by initiation of ER stress and induction of apoptosis [140,141], but not SOCE activation [142]. Furthermore, when activated, ER membrane TRPC6 was reported to regulate [Ca^2+^]_cyt_ and to induce the release of Ca^2+^ from the ER in platelets [143].

In addition to TRP Ca^2+^-permeable channels, other channels permeable to ions other than Ca^2+^, and localized to plasma membranes and/or intracellular membranes, could be involved in the mechanism of action of spiperone. In this context, several TRP channels (e.g., TRPM4, TRPM5, TRPM7, TRPM8) involved in the regulation of ion homeostasis and control of cell membrane potential were reported to be activated by PI(4,5)P2 [135]. Following PLC activation, PI(4,5)P2 reduction might determine desensitization of TRP channels, causing cationic homeostasis imbalance (e.g., cytosolic Ca^2+^, Na^+^, and/or Mg^2+^ imbalance) and membrane potential dysregulation [144,145]. We can speculate that spiperone, via an improper activation of PLC, might act through a dual mechanism of action: on the one hand, it induces sustained release of Ca^2+^ from intracellular stores, resulting in loss of intracellular Ca^2+^ homeostasis and, on the other hand, it causes the reduction in PI(4,5)P2 concentration leading to desensitization of TRP channels on the plasma membrane, resulting in dysregulation of ions and membrane potential. However, further investigation is necessary in order to address the role of TRP and membrane potential disruption in spiperone-induced cell death.

Physiological activation of Ca^2+^ channels normally results in transient and short but robust cytosolic Ca^2+^ peaks per minute, which mediate a broad repertoire of cellular functions, including proliferation, differentiation, migration, and secretion. On the other hand, long-lasting cytosolic Ca^2+^ increase, with similar strength to that of maximum peaks of calcium oscillation, could instead lead to apoptosis [146]. Regardless of the triggering circumstances, the onset of apoptosis always involves Ca^2+^ influx via cytoplasmic, mitochondrial, or ER-mediated mechanisms [56,147,148]. The frequency and amplitude of [Ca^2+^]_cyt_ are decoded by several intracellular effector proteins characterized by Ca^2+^-sensing motifs [149]. Therefore, we investigated several cytosolic Ca^2+^-dependent pathways activated in CRC cells upon spiperone treatment—including PKC, calmodulins, calpains, and CAMKII—but none of them appeared to be involved in spiperone-induced cell death.

The ER is a multifunctional organelle with strictly controlled homeostasis, where processes such as lipid biosynthesis and protein folding along with Ca^2+^ storage or release take place [65]. We outlined spiperone’s activity on the ER by monitoring [Ca^2+^]_ER_ during long-term treatment. Our data revealed not only a considerable long-lasting release of Ca^2+^ from the ER under acute spiperone treatment, but also a significant increase in [Ca^2+^]_ER_ over time [150,151]. Notably, the reduction in spiperone-induced cell mortality observed in cells co-treated with BAPTA was associated with significant inhibition of spiperone-induced [Ca^2+^]_ER_ overload. Moreover, inhibition of SERCA pumps with TG revealed enhanced Ca^2+^ release from intracellular Ca^2+^ stores, possibly as a consequence of [Ca^2+^]_ER_ overload, and evidenced by a massive increase in [Ca^2+^]_cyt_ in spiperone-treated cells after TG treatment compared with controls.

It is well known that cancer cells are usually characterized by low [Ca^2+^]_ER_, resulting in low cytosolic Ca^2+^ release [56] and, hence, decreased pro-apoptotic mitochondrial Ca^2+^ transfer and greater cell survival [152]. There is evidence of an association between increased [Ca^2+^]_ER_ and induction of apoptosis in cancer cells. In particular, the chemotherapeutic agent Adriamycin was reported to lead to ER Ca^2+^ overload by inducing a p53-dependent change in the oxidative state of SERCA at the ER, resulting in enhanced Ca^2+^ transfer to the mitochondria and apoptosis [153]; we cannot exclude the possibility that, in our experimental model, alteration of SERCA activity by spiperone could also be involved in [Ca^2+^]_ER_ overload. Altogether, this evidence suggests that the disruption of ER/cytosolic Ca^2+^ homeostasis maintenance is strongly associated with spiperone antitumor activity, and that modulation of [Ca^2+^]_ER_ is likely an effective therapeutic target in cancer cells.

The ER serves many specialized functions in the cell, including calcium storage and signaling, production of phospholipids and sterols, and biosynthesis of membrane and secretory proteins. Although several functions are at least partially performed in different areas of the ER, they are not entirely independent of one another. Importantly, disturbance of any of these functions—several of which are coupled with the ER Ca^2+^ level—can lead to so-called ER stress [154]. RNA sequencing analysis in both adherent and stem-like CRC cells treated with spiperone revealed a significant upregulation of biological processes associated with ER stress, UPR activation, and ER-stress-associated cell death.

[Ca^2+^]_ER_, similarly to [Ca^2+^]_cyt,_ is finely regulated by ER Ca^2+^-buffering proteins, pumps, exchangers, and channels [155], and whereas ER Ca^2+^ depletion is a well-known cause of ER stress [154], the relationship between [Ca^2+^]_ER_ overload and ER stress is yet to be clarified.

Evidence of Ca^2+^ overload associated with disruption of ER functions and accumulation of unfolded and misfolded proteins has been reported [156,157], and supports our data that link [Ca^2+^]_ER_ overload observed upon spiperone stimulation with ER stress, UPR and, eventually, cell death.

Interestingly, among the 158 DEGs identified in our cell lines treated with spiperone for 20 h, several upregulated genes encode ER proteins whose activity is strictly associated with Ca^2+^ binding and buffering. In particular, the gene *CRT*, encoding for calreticulin—an ER-resident Ca^2+^-buffering and -binding protein, and molecular chaperone [158]—was significantly upregulated, with log2FC of ~1.6 in both of the tested cell lines. Previous studies performed in amyotrophic lateral sclerosis reported that calreticulin-overexpressing cells display an increased total amount of [Ca^2+^]_ER_, as well as elevated ER Ca^2+^-pumping activity [159]. Moreover, the heat shock protein 90 beta family member 1 (*HSP90B1*) gene, encoding for the ER Ca^2+^-buffering protein grp94 [160], was found to be significantly upregulated in our cells (Log2FC > 2.3). Another significantly upregulated gene (Log2FC > 1.5 in both cell lines) was the protein disulfide isomerase family A member 3 (*PDIA3*) gene, encoding for the ER-resident protein ERp57 (also known as grp58), reported to regulate SERCA activity by modulating the redox state of its sulfhydryl groups [161].

The UPR is an essential adaptive mechanism that promotes cell survival; however, in case of severe or irreparable damage, prolonged UPR shifts from pro-survival to pro-death signaling, leading to activation of intrinsic apoptotic and autophagic pathways [74]. Starting from RNA sequencing results, analysis of UPR-induced signaling pathways confirmed early activation of three well-characterized signaling branches: PERK, IRE1, and ATF6 [162].

CHOP overexpression, along with its nuclear localization, further confirmed the link between spiperone-induced ER stress and cell death. CHOP is known to induce overexpression of proapoptotic proteins of the BCL2 family, resulting in mitochondrial impairment, release of cytochrome C, and activation of caspases [163]. Additionally, CHOP directly activates GADD34 (DNA damage protein), which combines with protein phosphatase 1 (PP1) to dephosphorylate eIF2α, resulting in protein translation recovery, increased ER stress, and cell apoptosis [164]. In our cells, CHOP gene silencing and chemical mitigation of ER stress significantly rescued spiperone-induced cell death, confirming the role of ER stress in the induction of apoptosis. Moreover, CHOP nuclear localization was antagonized by intracellular Ca^2+^ chelation, U73122 co-treatment, and PLCβ1/PLCε silencing, further confirming the relationship between PLC activation, ER stress, and cell death.

In addition to UPR activation, RNA-Seq analysis revealed that the most common significantly downregulated genes were associated with cancer cell proliferation and invasiveness. For example, CLDN2 expression was recently linked to increased incidence of CRC-associated liver metastasis [165], whereas CXCR4 [63] and CYP24A1 [64] are increasingly recognized as prognostic markers for CRC progression. PIF1 downregulation is usually associated with decreased survival in neoplastic cells, but not in nonmalignant cells [60], whereas PSRC1 has been confirmed to be involved in microsatellite instability (MSI)—a genetic condition resulting from incompetent DNA mismatch repair, and frequently associated with CRC incidence and progression [166].

Among the common significantly downregulated genes, we also identified genes known to be common targets of RIDD, including *TMEM19*, *BCAM*, and *DGAT2*. In particular, DGAT2 is a well-established target of RIDD, recently reported to be associated with both [Ca^2+^]_ER_ increase after induction of ER stress [167] and 1-O-acylceramide synthesis from ceramide and fatty acyl-CoA at the ER level [82,168].

Since the ER is the place where lipid synthesis occurs, and we confirmed a significant reduction in DGAT2 protein in both HCT116 and CRC-SC#1 cells, we performed lipidomic analysis after 20 and 40 h of treatment with spiperone. Notably, a significant alteration in SL representation was observed in both cell lines, in agreement with published data showing a relationship between SL alterations and apoptosis in cancer cells [169,170]. In fact, SL regulates several crucial cellular events, including cell proliferation, senescence, and migration, but also necrotic and apoptotic cell death [171,172].

In particular, HCT116 cells’ lipidomic profile was characterized by a significant reduction in Cer, along with a substantial increase in dihydroCer, dihydroHexCer, and SM. Recently, it was reported that ABTL0812—a small molecule with anticancer activity—leads to accumulation of dihydroCer and induction of ER stress, resulting in cancer cell death [173]. In addition, accumulation of dihydroCer has been associated with cell death in cancer cells treated with several antineoplastic drugs [174,175]. Since de novo ceramide biosynthesis depends on the ceramide synthase (CerS) and dihydroceramide desaturase (DEGS1) enzymes, anchored to the ER membrane [176], it is tempting to speculate as to the association of low levels of Cer observed in HCT116 cells with the impairment of CerS and DEGS1 during ER stress.

Contrary to what we observed in HCT116 cells, the SL profile identified in CRC-SC#1 cells was characterized by a significant increase not only in dihydroCer, but also in Cer. The role of Cer in cancer cells is now well established, with experimental evidence demonstrating its involvement in the mechanisms associated with differentiation, senescence, and blocking of cell growth [176,177]. Cer cytotoxicity is commonly associated with its transfer from the ER to the mitochondria, through mitochondria-associated membranes, resulting in mitochondrial permeabilization to apoptosis-inducing proteins [178]. Moreover, Cer accumulation was reported to lead to Bax-dependent apoptosis in numerous cancers (e.g., breast cancer, glioblastomas, prostate cancer, and colon cancer) [172]. In agreement with these observations, our results further confirmed the association between accumulation of Cer and induction of apoptosis in cancer cells. Similarly, del Solar et al., through comparative lipidomic analysis, demonstrated the accumulation of specific ceramides and dihydroceramides in CRC cells undergoing apoptosis, while no differences were observed in non-neoplastic cells [170].

Moreover, SM representation was affected by spiperone treatment. In particular, HCT116 cells were characterized by a wide overrepresentation of SM, whereas CRC-SC#1 cells displayed a significant overrepresentation of SM40:1;20A, SM42:1;20, and SM42:3,20/SM18:2;20/24. Notably, neoplastic cells are usually characterized by low basal levels of SM compared to non-neoplastic cells [179]; thus, drugs inducing increases in SM levels are of great interest in the context of anticancer treatments. In this context, the activity of the antineoplastic drug 2-hydroxyoleic acid was associated with activation of SM synthases, increased levels of SM, and cell death in glioma cells [180].

The alteration in SL metabolism associated with spiperone treatment could also contribute to mitochondrial inner membrane depolarization and the reduction in mitochondrial activity observed after long-term treatment with the drug, since it has been demonstrated that excessive sphingolipid accumulation in the mitochondria is associated with complex III irreversible inhibition, inner membrane depolarization, mitochondrial fragmentation, and mitophagy [181,182,183,184]. Moreover, MCU inhibition did not reduce spiperone-induced depolarization, excluding a direct role of ER Ca^2+^ influx into the mitochondria through MAMs in the impairment of the mitochondrial function [56,185]. These observations support the hypothesis that mitochondrial depolarization is likely to be a consequence of ER-stress-induced apoptosis and lipid metabolism dysfunction.

Along with the ER and mitochondria, the Golgi apparatus is tightly involved in the de novo synthesis of SLs, as well as in their metabolism [186,187]. In particular, Cer are synthesized in the ER and are transported to the GA, via the soluble transporter protein CERT, in order to be processed to SMs [188,189]; in addition, several enzymes involved in SL metabolism were reported to translocate from the ER to the GA under stress conditions [190]. Deregulation in lipid metabolism results in their accumulation in the GA, leading to vesicular trafficking dysfunction and cell death [83]. Consistent with the literature and our lipidomic data, spiperone treatment induced GA swelling in both HCT116 and CRC-SC#1 cells. We further confirmed, in HCT116 cells, that GA swelling was considerably reduced by compounds effective in attenuating spiperone-induced ER stress. These results underline the tight interaction between ER stress and GA swelling, suggesting that spiperone activity in CRC cells is linked not only to ER stress induction, resulting in alteration of lipid biosynthesis, but also in long-term irreversible mitochondria and GA impairment, resulting in cell death.

## 5. Conclusions

In conclusion, our data demonstrate that the antipsychotic drug spiperone is effective in inducing apoptosis in CRC cells. The toxic action of the drug is due to PLC-dependent deregulation of intracellular calcium homeostasis, ER stress induction, and UPR-induced cell death. Lipidomic analysis revealed a disruption of the metabolism of phospholipids and sphingolipids, with alteration of sphingomyelin, dihydroceramide, and ceramide species. Damage to the mitochondria and the GA was also observed. The implications of our results are multifold: first, they demonstrate the important contribution of drug repurposing in identifying new therapies; second, they help to identify novel molecular targets and druggable signaling pathways that can be targeted in CRC; third, they highlight the potential effectiveness of drugs interfering with ER stress and lipid metabolism in cancer therapy. However, once the efficacy of a compound is extensively demonstrated, preclinical studies are needed in order to determine administration timings and dosages to reduce side effects. In addition, chemical and structural modification of the compounds could lead to the development of more effective molecules.

## Figures and Tables

**Table 1:** Silencer Select Pre-Designed siRNA sequences (5′–3′).

Target Gene Symbol	Sense	Antisense
PLCB1	GGACUUACGUGGAAGUAGAtt	UCUACUUCCACGUAAGUCCca
PLCB3	CGUCCUUUGUGGAGACCAAtt	UUGGUCUCCACAAAGGACGac
PLCG1	GGGUGAAAAAGAUCCGUGAtt	UCACGGAUCUUUUUCACCCag
PLCD3	GGUUUGUGGUGGAAGAUUAtt	UAAUCUUCCACCACAAACCgg
PLCE1	GCAGGAAAAUUCAUCCUUAtt	UAAGGAUGAAUUUUCCUGCac
DDIT3	GUAGUGAAUUGAUCUAGAUtt	AUCUAGAUCAAUUCACUACca

**Table 2:** Oligo sequences (5′–3′) for the genes investigated.

Target Gene	Forward	Reverse
CDKN1A	CCTCATCCCGTGTTCTCCTTT	GTACCACCCAGCGGACAAGT
CXCR4	CAGCAGGTAGCAAAGTGACG	ATAGTCCCCTGAGCCCATTT
CHOP	CATCACCACACCTGAAAGCA	TCAGCTGCCATCTCTGCAG
DGAT2	AAAGAATGGGAGTGGCAATG	TCCTCGAAGATCACCTGCTT
DR5	GAGCTAAGTCCCTGCACCAC	AATCACCGACCTTGACCATC
PIF2	CCCTGGATTGTGTGGAGATT	ACTCCAGACTGAGGCTCCTG
GADD34	CTCAAGCGCCCAGAAACC	CTCCTGGGCCTGGGTGAT
GRP78	GTTCTTGCCGTTCAAGGTGG	TGGTACAGTAACAACTGCATG
GUSB	ATCGCCATCAACAACAC	CTTGGGATACTTGGAGGTG
HPRT	AAGGACCCCACGAAGTGTTG	GGCTTTGTATTTTGCTTTTCC
PLCB1	GAGGCTAGAAGAAGCGCAAA	ATTGCTGTCTTCACTGATCTTTCCT
PLCB3	GCCTCAGAAGTCTCTGGGTG	GGACATCTCCTCAGTGGCAT
PLCG1	TGTCCCACAGACCAACGC	ATTCCGCTTCCGCACCAG
PLCE1	ACGTCTGTCAGAAGCCCAGT	GCTTTAAGCATGGACCAACG
PLCD3	CCAGAACCACTCTCAGCATCCA	TTGAAGCCATTGTTGAGCAC

**Table 3:** Summary of spiperone IC_50_ values measured by viability assay in different cell lines.

Cell Line	IC_50_ 72 h (95% CI) (μmol/L)
HCT116	7.10 (6.61–7.64)
HCT8	5.26 (4.83–5.72)
SW620	9.23 (8.55–9.97)
SW480	9.87 (8.97–10.9)
CRC-SC#1	3.76 (3.38–4.18)
CRC-SC#2	8.14 (7.38–8.98)
CRC-SC#3	4.13 (3.72–4.58)
CRC-SC#4	3.51 (3.16–3.92)
dd-HCT116 ^1^	5.62 (5.17–6.12)
PBMC	31.6 (30.3–32.9)
vAT-MSC	61.7 (53.3–71.4)
hDF1	98.0 (85.2–113)

^1^ dd-HCT116: dedifferentiated HCT116.

## Data Availability

Raw reads and processed sequencing data were deposited in the NCBI Gene Expression Omnibus, and are publicly available under accession number GSE190093.

## Supplementary Materials

The following are available online at https://www.mdpi.com/article/10.3390/cancers14030776/s1: Figure S1: Spiperone reduces the cell viability of CRC cell lines; Figure S2: Colorectal cancer cells’ sensitivity to spiperone; Figure S3: Spiperone modulates intracellular Ca^2+^ kinetics; Figure S4: Characterization of spiperone-dependent PLC activation; Figure S5: Spiperone-induced ER Ca^2+^ release is not mediated by SERCA inhibition; Figure S6: BAPTA-AM and U73122 rescue spiperone-induced apoptosis; Figure S7: PLC δ3 is involved in spiperone-dependent acute modulation of intracellular Ca^2+^; Figure S8: [Ca^2+^]_ER_ accumulation is reduced by BAPTA-AM; Figure S9: Spiperone induces PKC and AMPK activation; Figure S10: Involvement of Ca^2+^ interacting proteins under spiperone treatment; Figure S11: Effect of GPCR and RTK signaling inhibitors on spiperone-induced acute Ca^2+^ modulation; Figure S12: Spiperone activates the MAPK pathway; Figure S13: RNA sequencing of spiperone-treated CRC cells revealed XBP1 splicing; Figure S14: Spiperone induces ER stress in CRC cells; Figure S15: Identification of CHOP/DDIT3 targets; Figure S16: Spiperone induces autophagy and mTOR pathway inhibition; Figure S17: Spiperone treatment alters mitochondrial respiration; Figure S18: Lipidomic results in CRC cells treated with spiperone for 20 and 40 h; Figure S19: Representation of the relative percentage (%) of the major lipid classes identified by HPLC–MS/MS; Figure S20: Western blot membranes; Table S1: RNA-Seq analysis *p*-value adjusted and log2FC values of the 158 common DEGs; Table S2: Lipidomic analysis of spiperone-treated CRC cells; Table S3: Fisher Exact Test.
