# Supplementary material for: Dissecting the Mechanism of Action of Spiperone—A Candidate for Drug Repurposing for Colorectal Cancer
Source: Cancers (Basel). 2022 Feb 2;14(3):776. doi: 10.3390/cancers14030776 (PMC8834219; doi:10.3390/cancers14030776)
Supplement: Supplementary file 1 [file cancers-14-00776-s001.zip › cancers-1526110-supplementary-done.pdf]

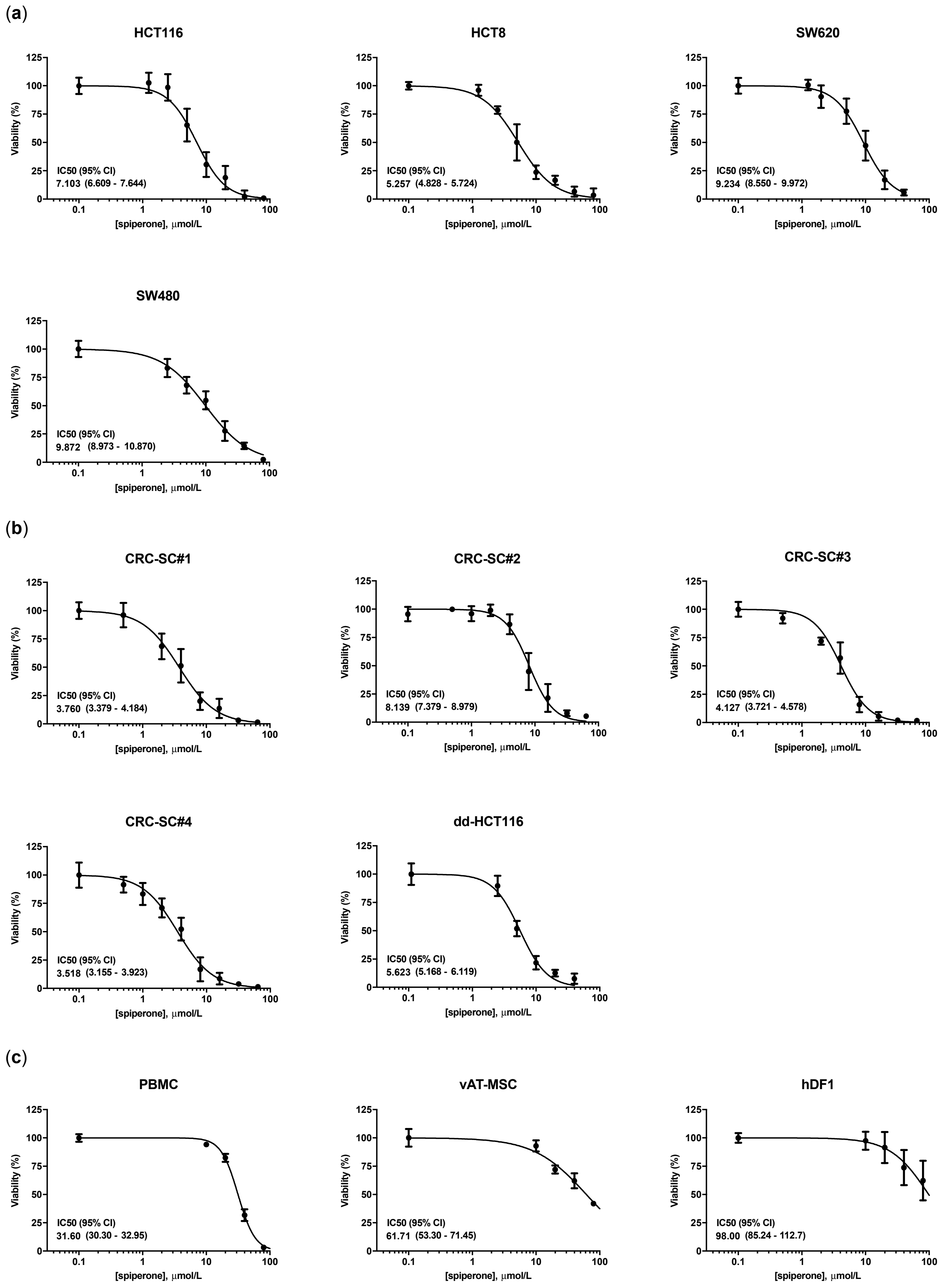

Figure S1. Spiperone reduces cell viability of CRC cell lines. Dose-response curves showing cytotoxic effect of spiperone on CRC cells (**a**), CRC-SCs (**b**) and non-neoplastic cells (**c**). Cells were treated with scalar concentrations of spiperone for

72 hours. The best fit values of IC50 values were calculated by using a variable slope model (GraphPad Prism 9). Each data point represents the mean of at least three independent experiments. IC50, drug concentration reducing by 50% viability compared to control; 95% CI, 95% confidence interval.

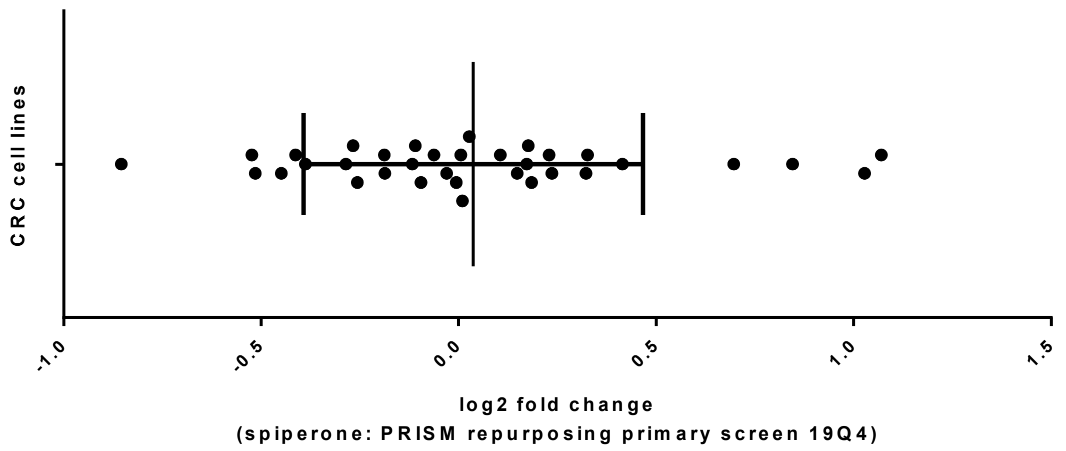

Figure S2. Colorectal cancer cells sensitivity to spiperone. Graph showing spiperone sensitivity of 34 different CRC cell lines expressed as log2 fold change relative to control. Data were obtained from PRISM repurposing primary screen 19Q4.

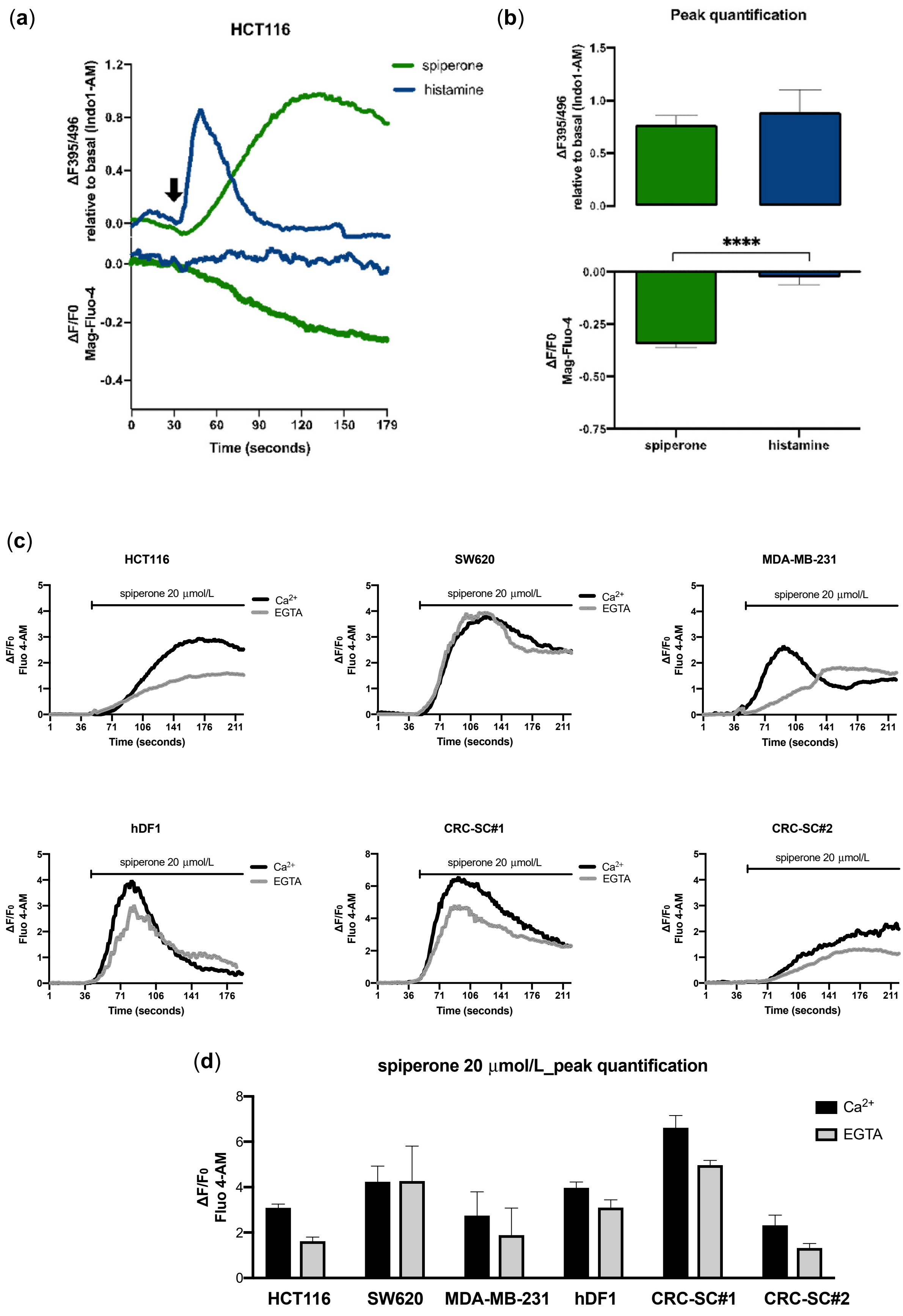

Figure S3. Spiperone modulates intracellular Ca^{2+} kinetics. Comparison of histamine and spiperone-induced Ca^{2+} release from the endoplasmic reticulum in the absence of extracellular Ca^{2+}. $[Ca^{2+}]_{cyt}$ (upper panel) and $[Ca^{2+}]_{ER}$ (lower panel) were

simultaneously evaluated before and after histamine and spiperone exposure (at the time indicated by the arrow), graph representing the mean of fluorescence kinetics over time (**a**). Histogram displaying fluorescence peaks quantification relative to basal signal for Indo-1 AM and Mag-fluo4-AM (**b**). Spiperone modulates intracellular Ca^{2+} kinetics in several cell lines. $[Ca^{2+}]_{cyt}$ was recorded before and after spiperone (20 µmol/L) exposure, in the presence and in the absence of extracellular Ca^{2+}, in CRC adherent cells (HCT116, SW620), breast cancer cell lines (MDA-MB-231), human dermal fibroblast (hDF1) and 2 CRC-SC lines (CRC-SC#1, CRC-SC#1). Graphs representing the mean of fluorescence kinetics over time in all the tested cell lines (**c**). Histograms displaying fluorescence peaks quantification relative to basal signal for Fluo-4 AM (**d**) Data are presented as mean ± SD from three independent experiments. Student's t-test ****p < 0.0001.

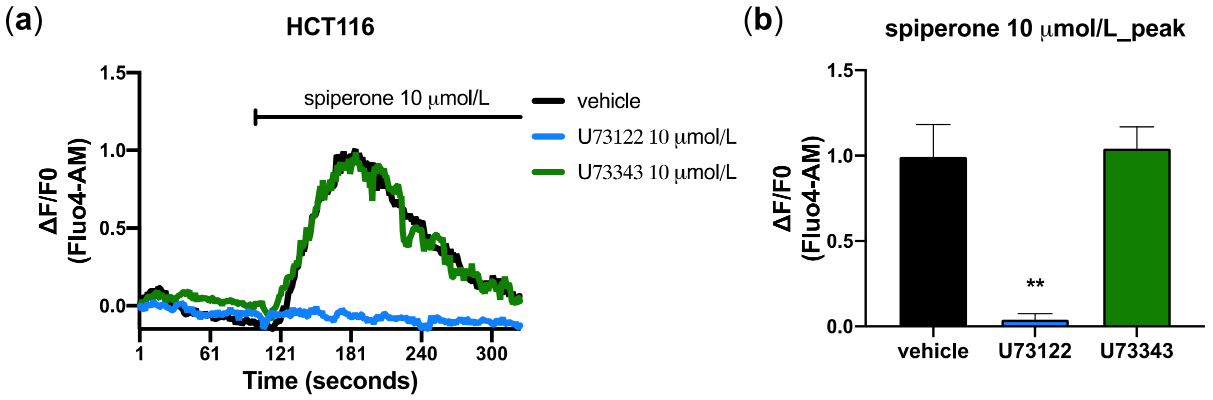

Figure S4. Characterization of spiperone-dependent PLC activation. $[Ca^{2+}]_{cyt}$ was recorded before and after spiperone (10 µmol/L) exposure, in the absence of extracellular Ca^{2+}, in cells pretreated with vehicle, 10 µmol/L U73122 or 10 µmol/L U73433. Graphs representing the mean of fluorescence kinetics over time (**a**). Histogram displaying fluorescence peaks quantification relative to basal signal for Fluo-4 AM (**b**). Data are presented as mean ± SD from at least three independent experiments, Student's t-test **p < 0.01.

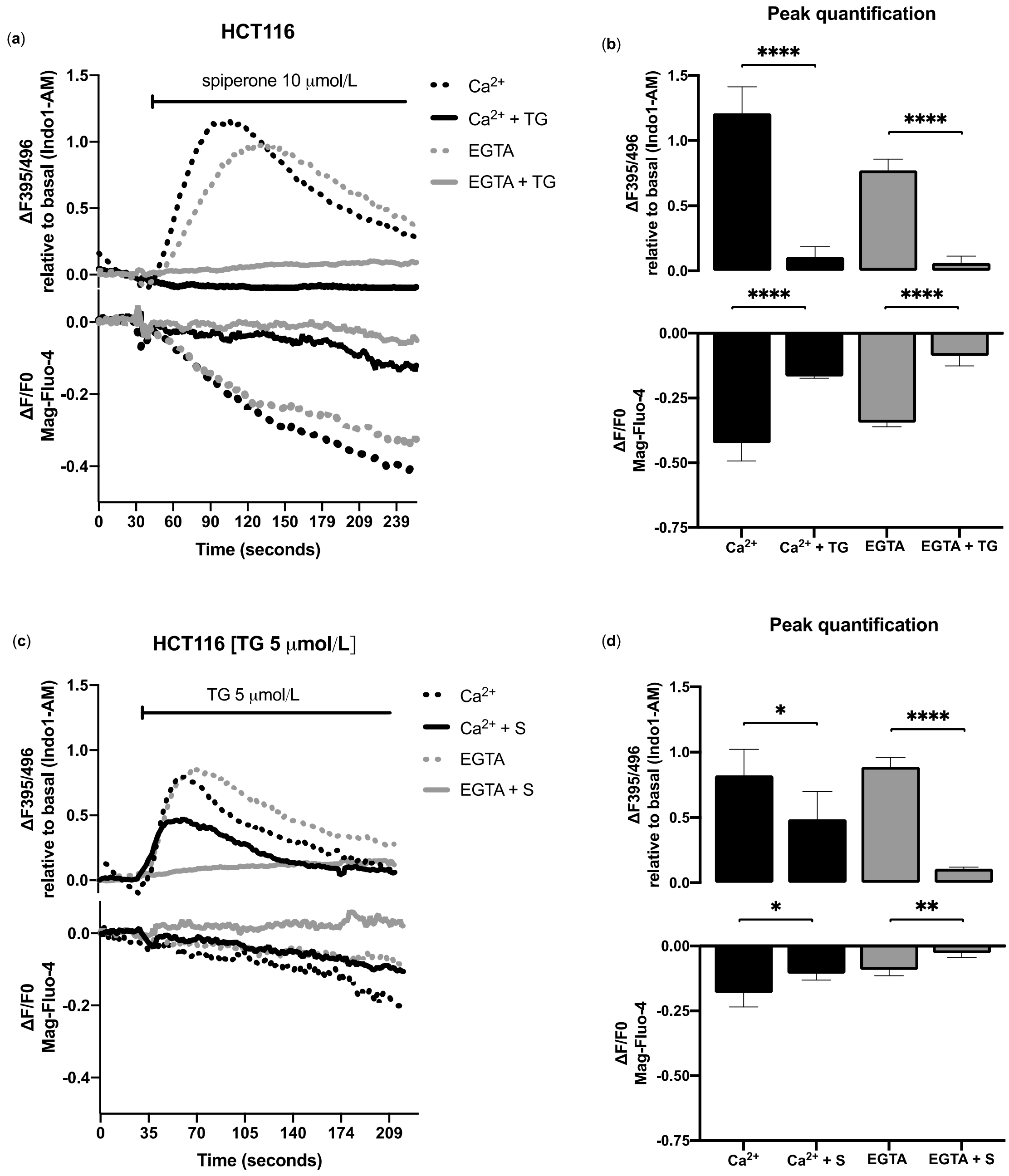

Figure S5. Spiperone-induced ER Ca^{2+} release is not mediated by SERCA inhibition. $[Ca^{2+}]_{cyt}$ (upper panel) and $[Ca^{2+}]_{ER}$ (lower panel) were simultaneously evaluated before and after spiperone exposure, graph representing the mean of fluorescence kinetics over time in presence of extracellular Ca^{2+} in cells pretreated for 1 hour with vehicle (dotted black line) or thapsigargin (TG) 5 μmol/L (solid black line) or without extracellular Ca^{2+} in cells pretreated with vehicle (dotted gray line) or TG 5 μmol/L (solid gray line) (**a**). Histogram displaying fluorescence peaks quantification relative to basal signal for Indo1-AM and Mag-fluo4-AM (**b**). $[Ca^{2+}]_{cyt}$ (upper panel) and $[Ca^{2+}]_{ER}$ (lower panel) were simultaneously evaluated before and after TG exposure, graph representing the mean of fluorescence kinetics over time in presence of extracellular Ca^{2+} in cells pretreated for 1 hour with vehicle (dotted black line) or spiperone (S) 10 μmol/L (solid black line) or without extracellular Ca^{2+} in cells pretreated with vehicle (dotted gray line) or spiperone 10 μmol/L (solid gray line) (**c**). Histogram displaying fluorescence peaks quantification relative to basal signal for Indo1-AM and Mag-fluo4-AM (**d**). Data are presented as mean ± SD from at least three independent experiments. Student's t-test *$p < 0.05$; Student's t-test **$p < 0.01$; Student's t-test ****$p < 0.0001$.

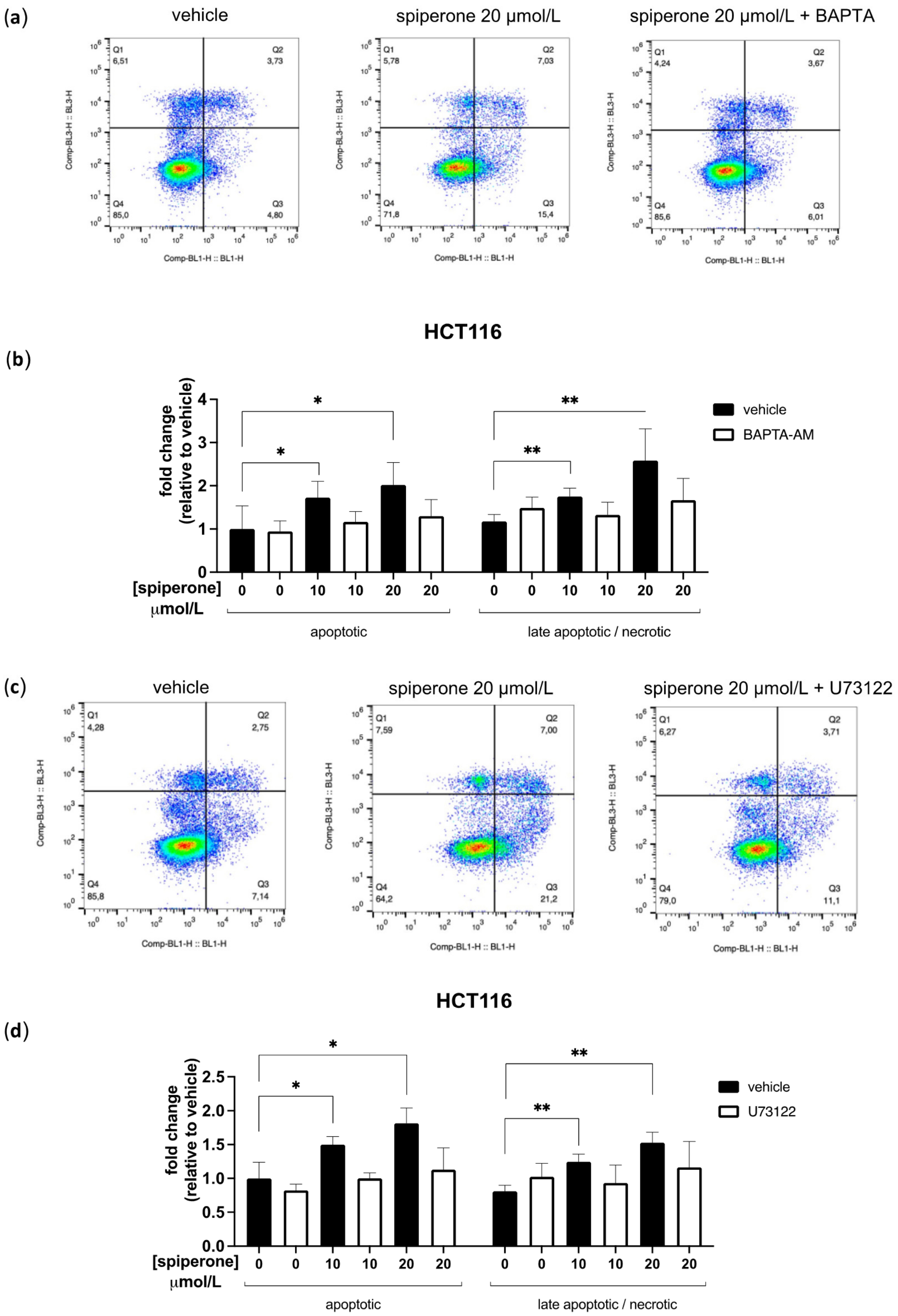

Figure S6. BAPTA-AM and U73122 rescue spiperone-induced apoptosis. Representative dot plots showing cell distribution of HCT116 treated for 24 hours with spiperone alone or in combination with BAPTA-AM after Annexin V/PI (Ax/PI) staining (**a**). Graph showing the analysis of HCT116 cells treated with different concentrations of spiperone alone or in combination with BAPTA-AM 10 µmol/L (**b**). Representative dot plots showing cell distribution of HCT116 treated for 24 hours with spiperone alone or in combination with U73122 after (Ax/PI) staining (**c**). Graph showing the analysis of HCT116 cells treated with different concentrations of spiperone alone or in combination with U73122 1 µmol/L (**d**). *, Student's T-test $p < 0.05$; **, Student's T-test $p < 0.01$.

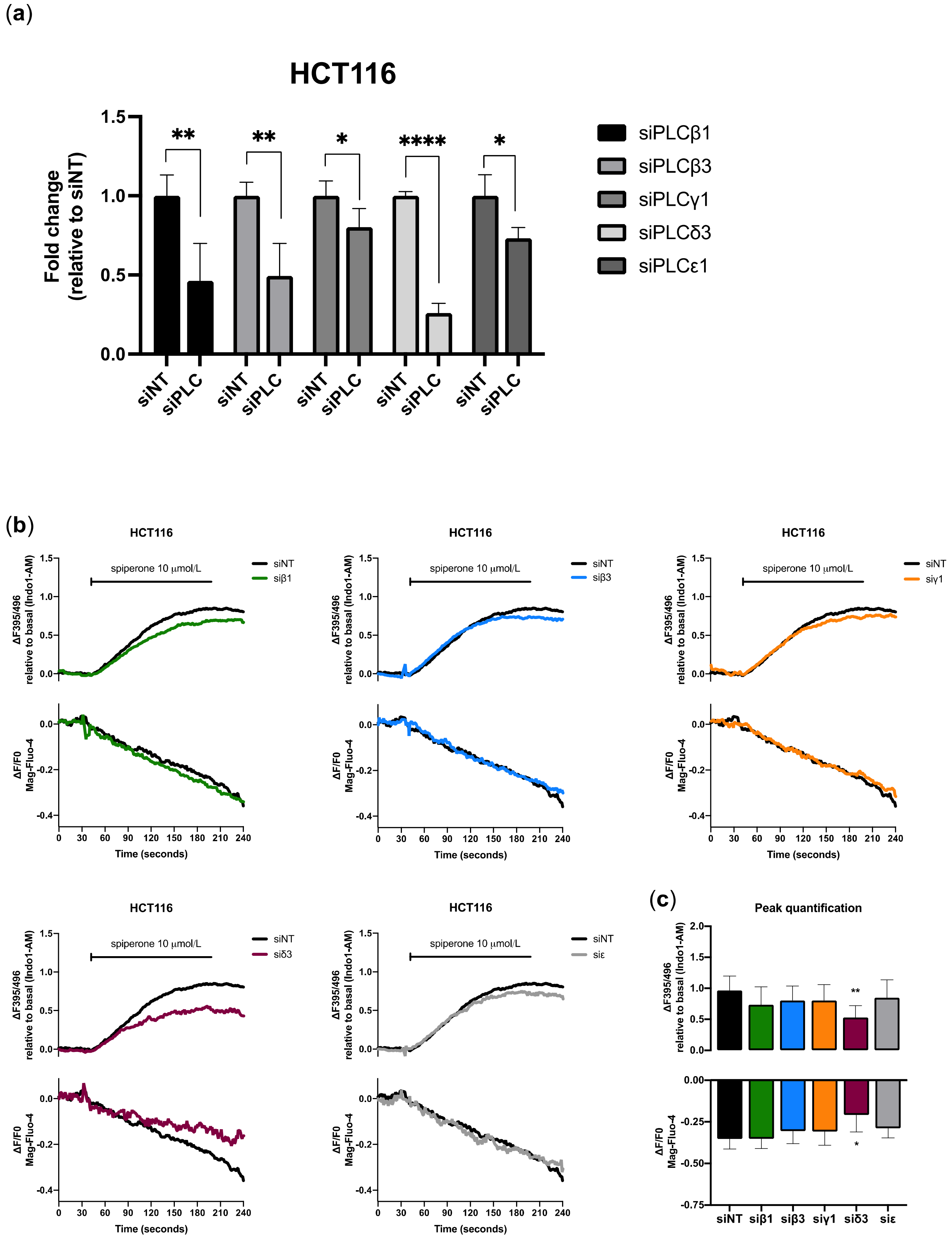

Figure S7. PLC δ3 is involved in spiperone-dependent acute modulation of intracellular Ca^{2+}. Gene expression analysis of PLC β1, β3, γ1, δ3 and ε1 by RT-qPCR 48 hours post-transfection. Relative expressions were determined by the ΔΔCt method and normalized with the control gene HPRT (**a**) [Ca^{2+}]$_{cyt}$ (upper panel) and [Ca^{2+}]$_{ER}$ (lower panel) were simultaneously evaluated before and after 10 µmol/L spiperone exposure in the absence of extracellular Ca^{2+}, graphs representing

the mean of fluorescence kinetics over time in cells silenced for PLC β1, β3, γ1; δ3 and ε1 (**b**). Histogram displaying fluorescence peaks quantification relative to basal signal for Indo-1 AM and Mag-fluo4-AM (**c**). Data are presented as mean ± SD from at least three independent experiments. Student's t-test *p < 0.05; Student's t-test **p < 0.01; Student's t-test ****p < 0.0001.

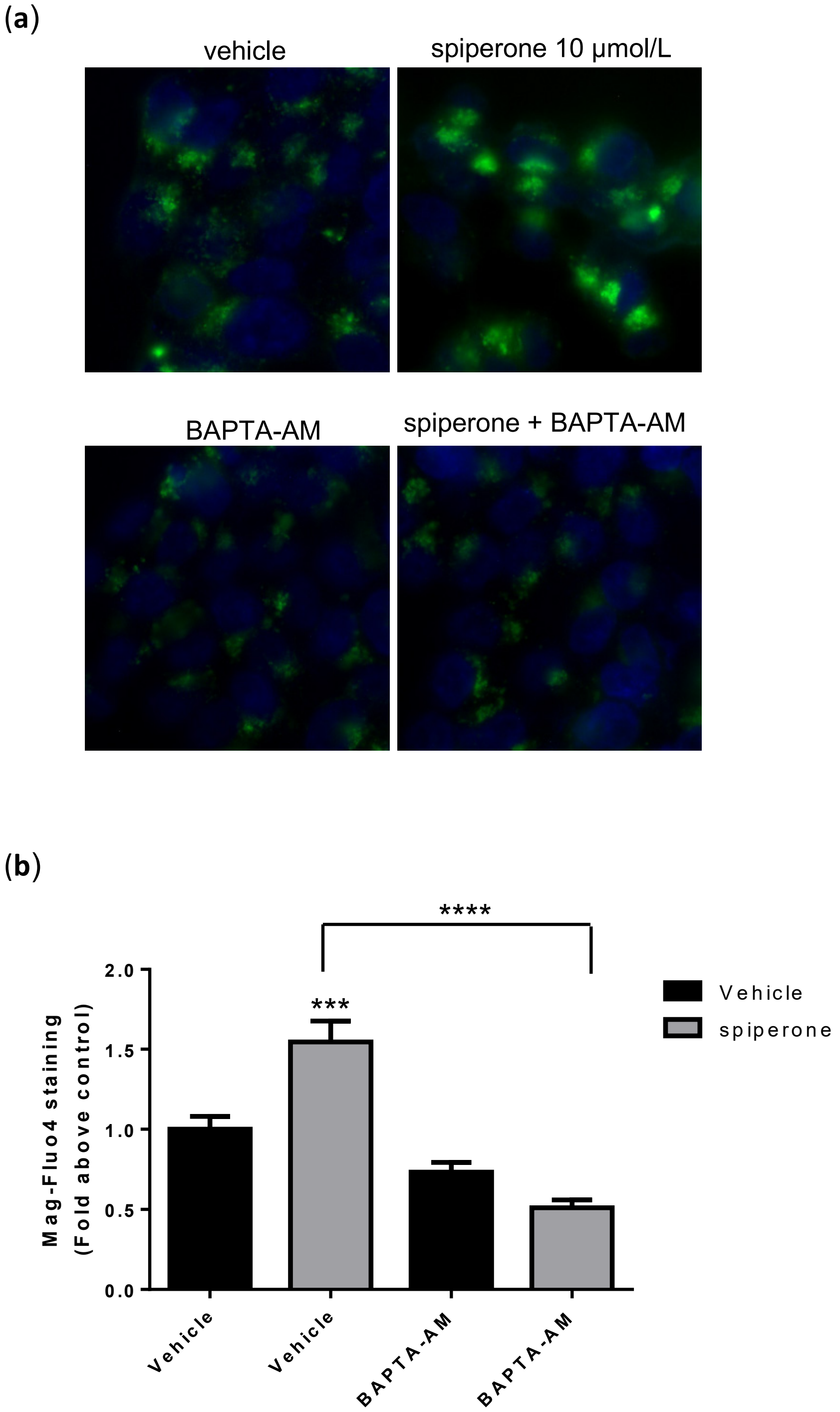

Figure S8. $[Ca^{2+}]_{ER}$ accumulation is reduced by BAPTA-AM. HCT116 cells were treated with vehicle or spiperone 10 µmol/L in combination with BAPTA-AM 10 µmol/L. $[Ca^{2+}]_{ER}$ was evaluated by immunofluorescence using the fluorescent probe Mag-Fluo-4 AM (Green). Nuclei were stained with Hoechts 33342 (blue) (**a**). Histogram Mag-Fluo-4 AM staining/ nuclei staining as fold change relative to control (**b**). Data are presented as mean ± SD of three independent experiments, each performed in triplicate. Student's T-test ***p < 0.001; Student's T-test ****p < 0.0001.

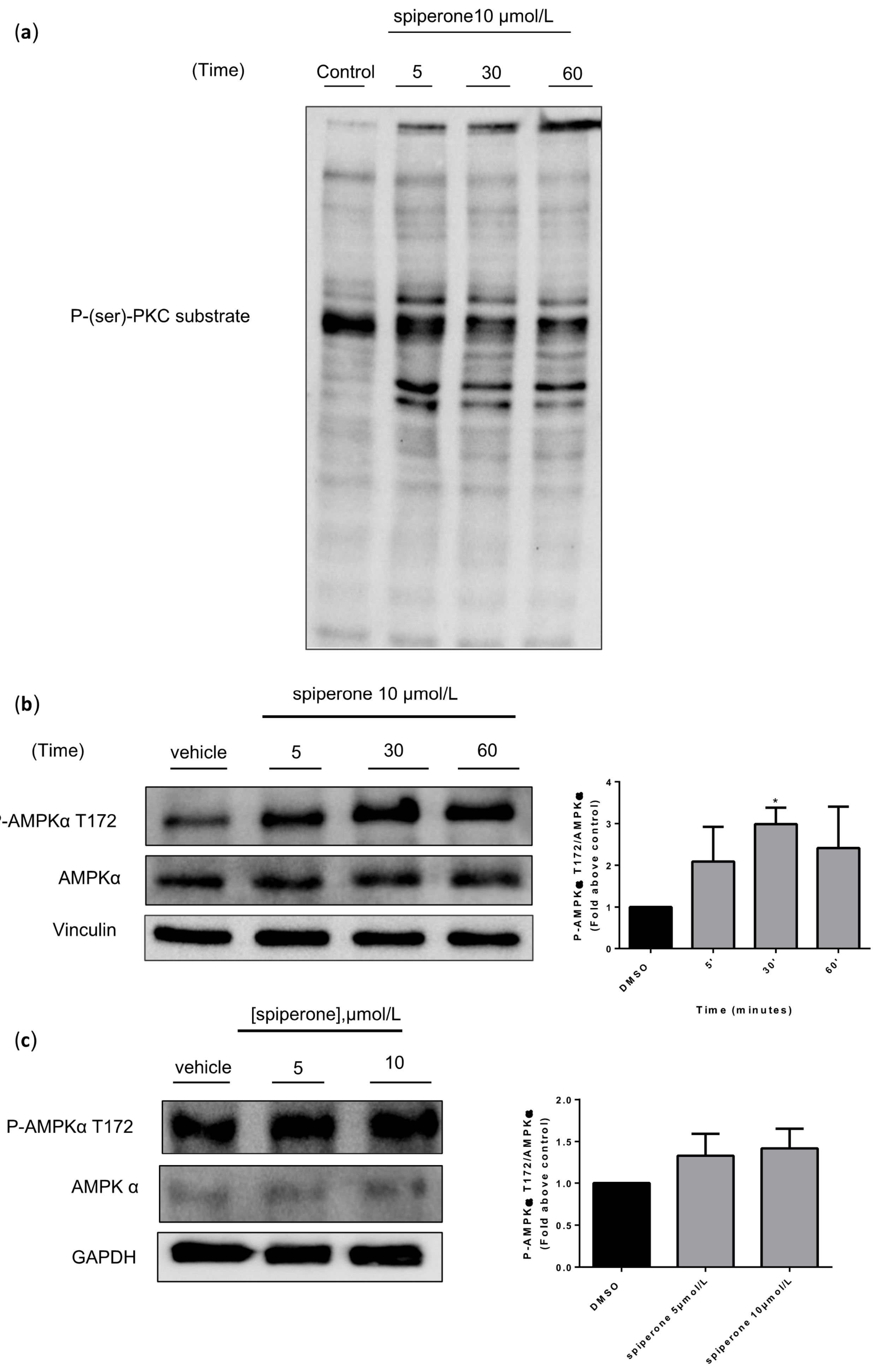
(a)
spiperone10 μmol/L
(Time)
Control
5
30
60
P-(ser)-PKC substrate
(b)
spiperone 10 μmol/L
(Time)
vehicle
5
30
60
P-AMPKα T172
AMPKα
Vinculin
(Fold above control)
0
1
2
3
4
*
DMSO
5'
30'
60'
Time (minutes)
(c)
[spiperone],μmol/L
vehicle
5
10
P-AMPKα T172
AMPK α
GAPDH
(Fold above control)
0.0
0.5
1.0
1.5
2.0
DMSO
spiperone 5μmol/L
spiperone 10μmol/L

Figure S9. Spiperone induces PKC and AMPK activation. Western blot analysis of P-(ser)-PKC in HCT116 cells treated with spiperone 10 µmol/L at several time points (**a**). Western blot analysis of HCT116 cells treated with spiperone 10 µmol/L at several time points (**b**) and after 16 hours treatment with spiperone 5 and 10µmol/L (**c**). Lysates were analyzed for p-AMPK T172, AMPK and GAPDH, and P-PKC substrate. Representative images of three independent experiments. Student's t-test *p < 0.05.

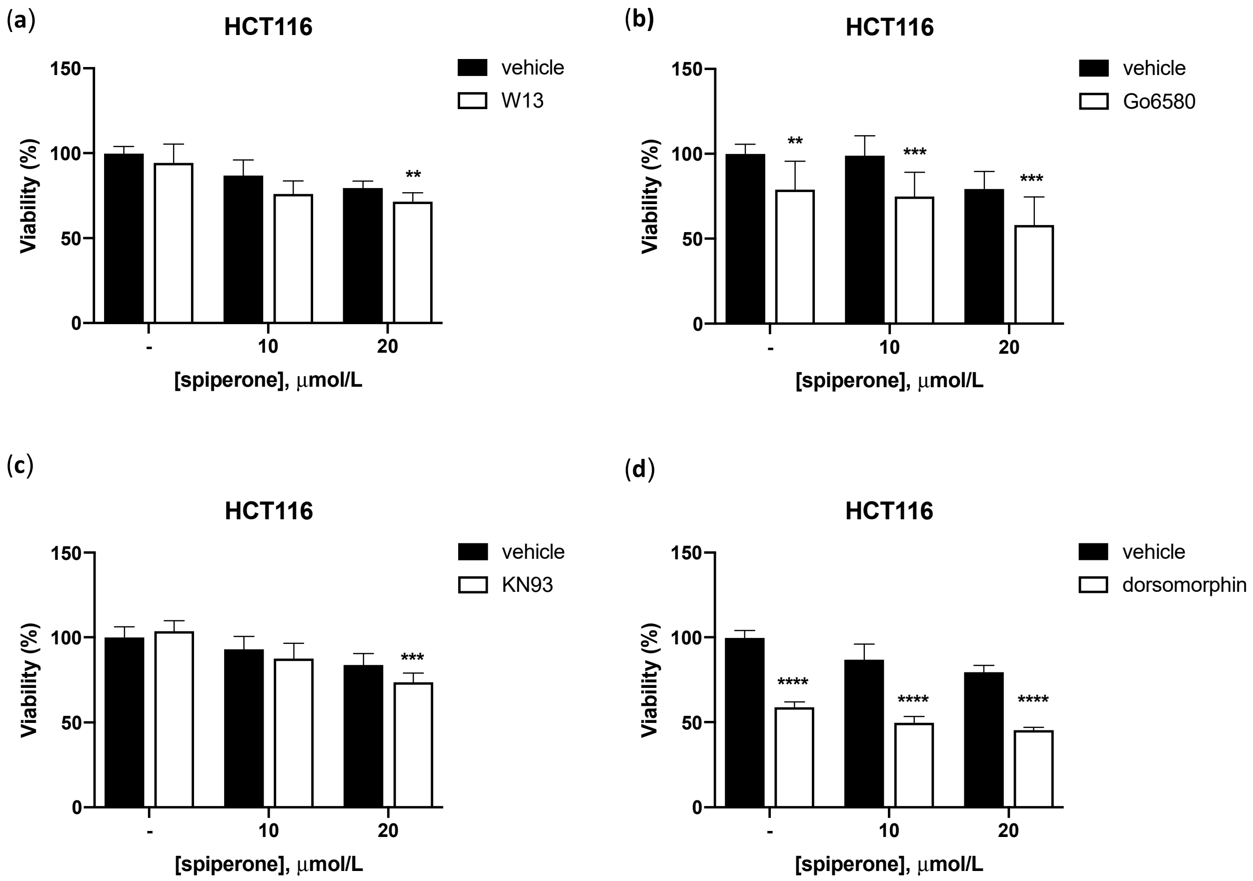

Figure S10. Involvement of Ca^{2+} interacting proteins under spiperone treatment. Effect of co-treatment of spiperone and calmodulin inhibitor W13 10 µmol/L (**a**), PKC inhibitor Go6850 1 µmol/L (**b**), CAMK inhibitor KN93 10 µmol/L (**c**) and AMPK inhibitor dorsomorphin 5 µmol/L (d). After 30 minutes of pretreatment, HCT116 cells were treated with vehicle or spiperone 10 or 20 µmol/L. Graphs displaying cell viability as the percentage of viable cells. Data show mean ± SD of at least three independent experiments performed in triplicate. Student's t-test **p < 0.01; Student's t-test ***p < 0.001; Student's t-test ****p < 0.0001.

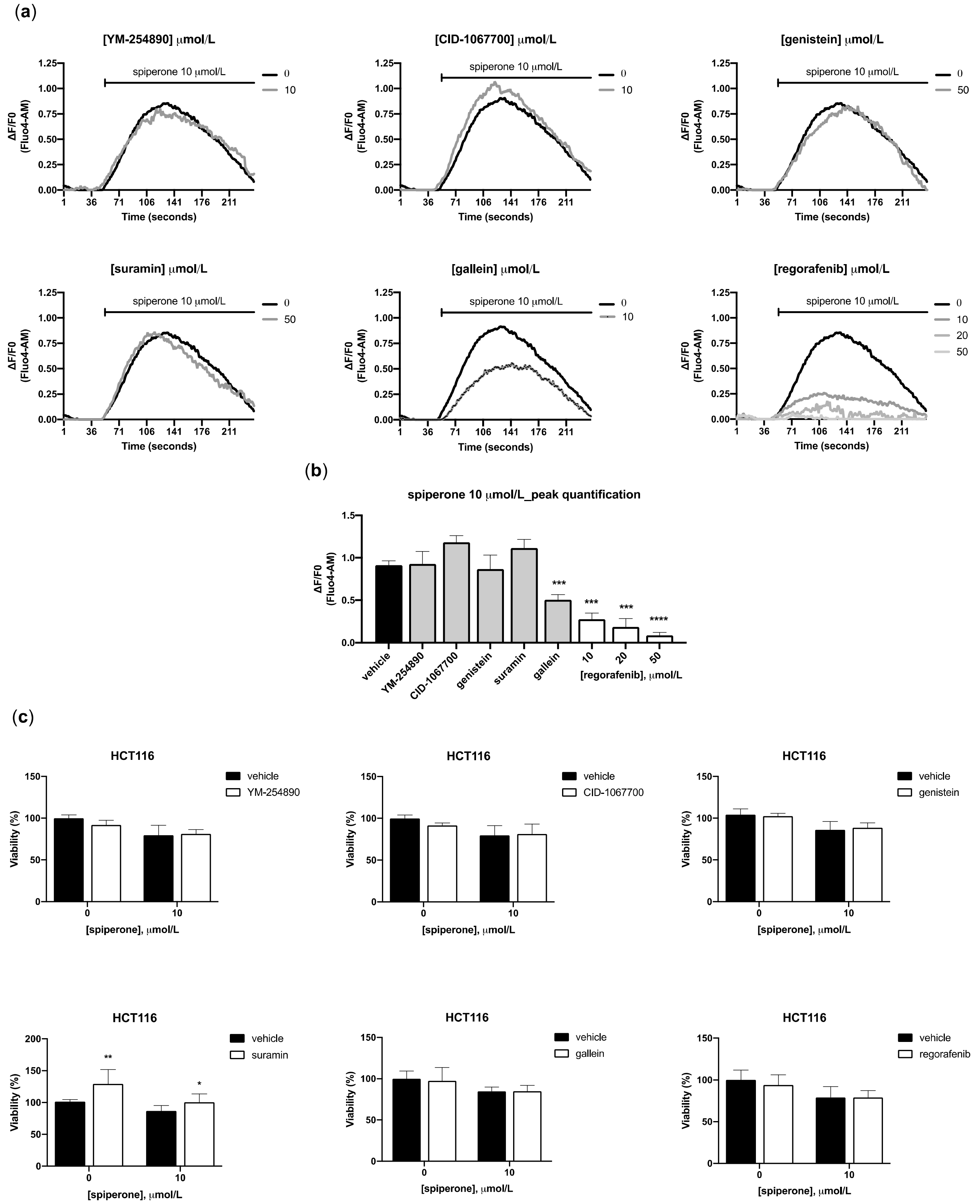

Figure S11. Effect of GPCR and RTK signaling inhibitors on spiperone-induced acute Ca^{2+} modulation. $[Ca^{2+}]_{cyt}$ was evaluated before and after spiperone exposure in cells pretreated with vehicle, tyrosine kinases inhibitors (genistein, 50 μmol/L, and regorafenib at several doses), small G proteins inhibitor (CID-1067700, 10 μmol/L), and G subunit inhibitors (Gβγ: gallein, 10 μmol/L; Gαq: YM-254890, 10 μmol/L; Gαs: suramin, 50 μmol/L). Graph representing the mean of fluorescence kinetics over time (**a**). Histogram displaying fluorescence peaks quantification relative to basal signal for Fluo-4 AM (**b**). Data are presented as mean ± standard deviation from three independent experiments. Student's t-test *** $p < 0.001$, Student's t-test ****$p < 0.0001$. Effect of 10 μmol/L spiperone cotreatment and aforementioned inhibitors in HCT116 cells,

(genistein 50 µmol/L, regorafenib 1 µmol/L, gallein 10 µmol/L, YM-254890 10 µmol/L, suramin 10 µmol/L). Graphs displaying cell viability as the percentage of viable cells. Data show mean ± SD of at least three independent experiments performed in triplicate, Student's t-test * $p < 0.05$.

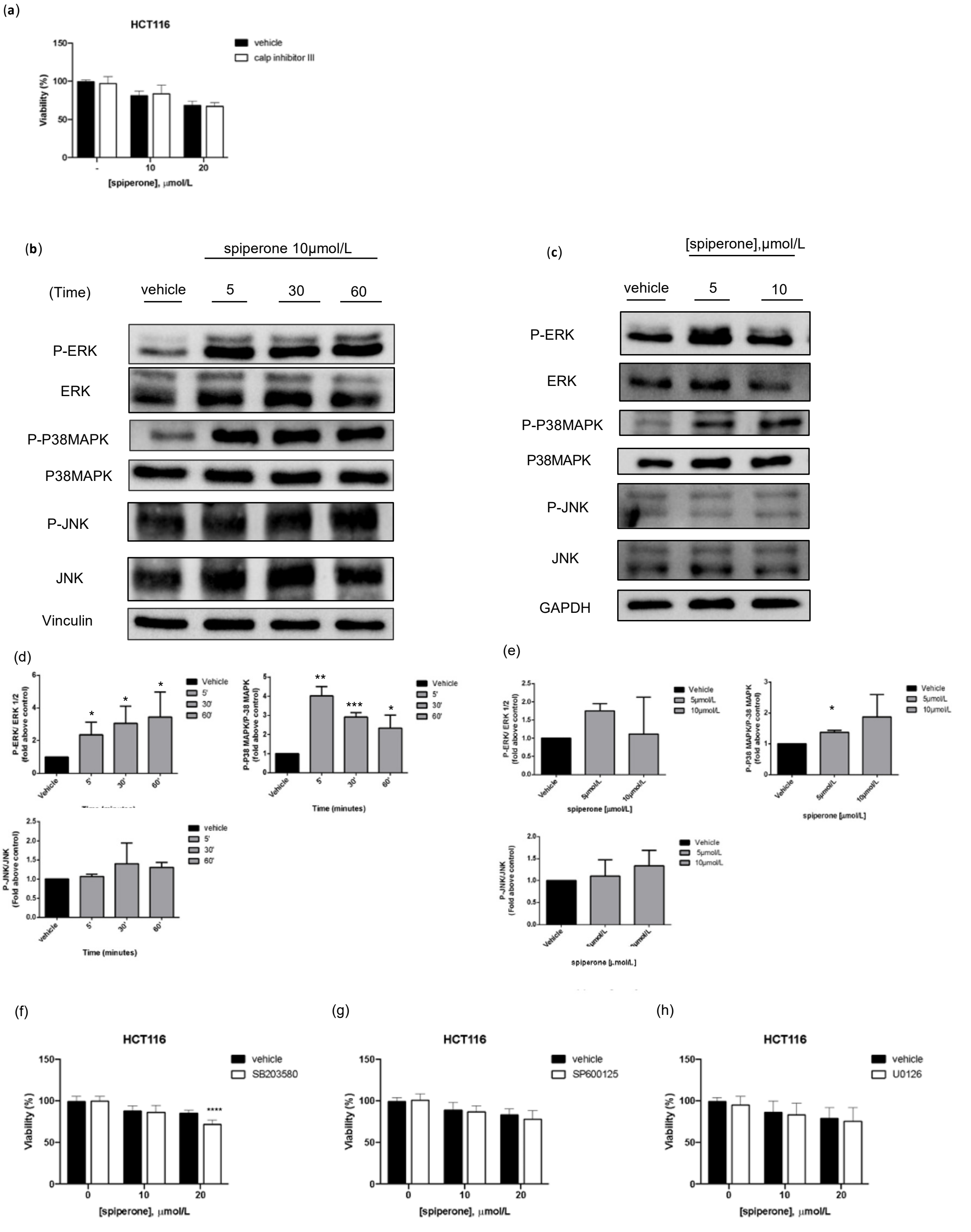

Figure S12. Spiperone activates the MAPK pathway. Effect of co-treatment of spiperone and calpain inhibitor III. After 30 minutes of pretreatment, HCT116 cells were treated with vehicle or spiperone at 10 or 20 µmol/L (**a**). Graphs displaying cell viability as the percentage of viable cells. Data show mean ± SD of at least three independent experiments performed in triplicate. Western blot analysis of MAPK signaling in HCT116 cells. Cells were analyzed at 5, 30, and 60 minutes after treatment with 10µmol/L spiperone (**b**) Western blot analysis of HCT116 cells after 16 hours treatment with 5 and 10 µmol/L spiperone (**c**) Histogram showing quantification of P-ERK1/2, P-P38MAPK and P-JNK, normalized on ERK1/2, P38MAPK and JNK respectively, at the different time points (**d**). Histogram showing quantification of P-ERK1/2, P-P38MAPK, and P-JNK, normalized on ERK1/2, P38MAPK, and JNK respectively, after 16 hours treatment with spiperone

(**e**). Effect on cell viability of co-treatment of spiperone and P38-MAPK inhibitor SB203580 10μmol/L (**f**), JNK inhibitor SP600125 10μmol/L (**g**) and MEK inhibitor U0126 10 μmol/L (**h**). After 30 minutes of pretreatment, HCT116 cells were treated with vehicle or spiperone at 10 or 20 μmol/L. Graphs displaying cell viability as the percentage of viable cells. All data are presented as mean ± SD from three independent experiments. Student's t-test * $p < 0.05$; Student's t-test ** $p < 0.01$; Student's t-test ***$p < 0.001$; Student's t-test ***$p < 0.0001$.

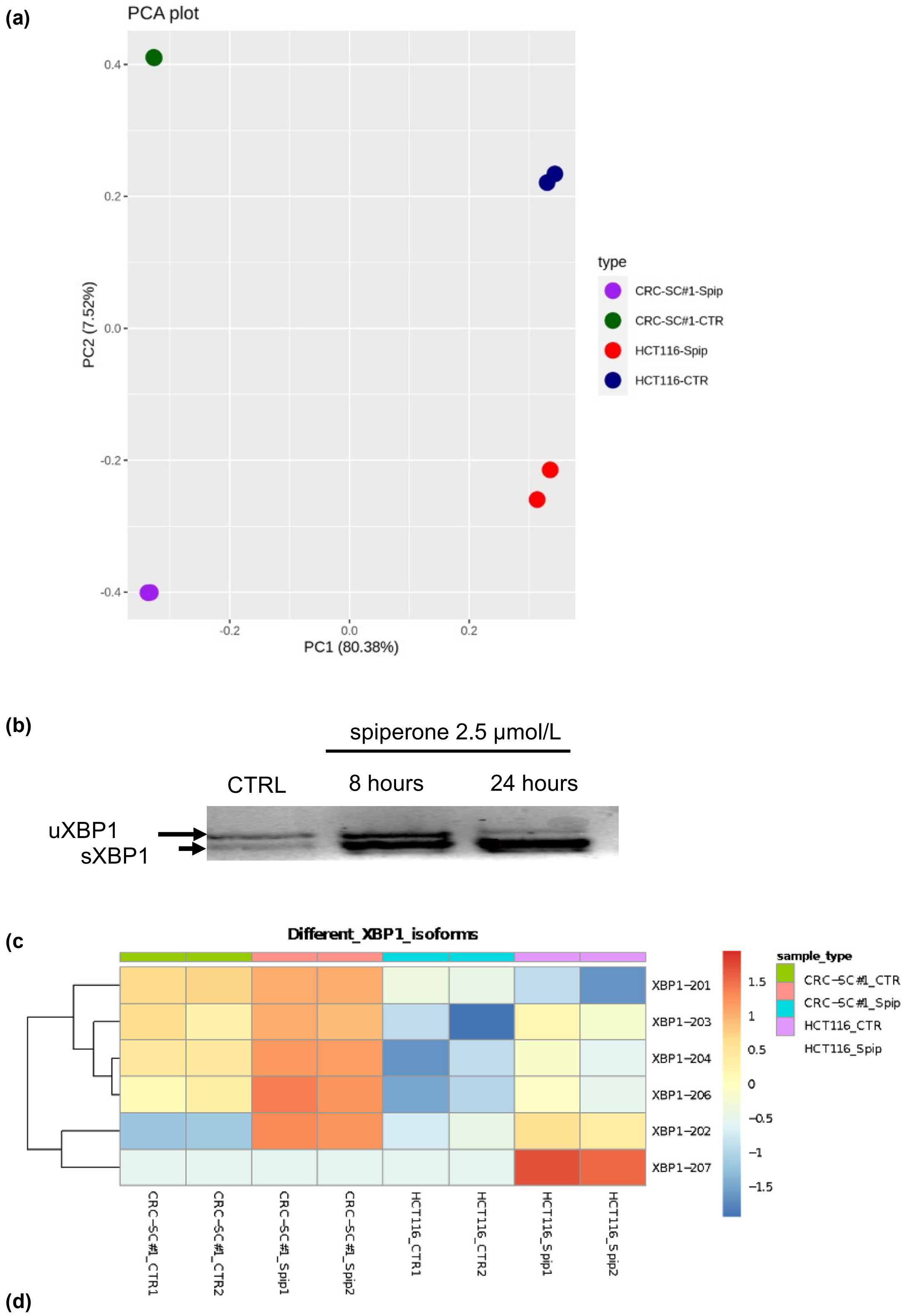

(d)

Query	Log2FC HCT116	Log2FC CRC-SC#1	Name	Subject	Protein	%id	alignment_length	query_start	query_end	subject_start	subject_end	E_value	bit_score
ENST00000216037.10	-1,89		XBP1-201	Unspliced	P17861-1	100	57	552	608	1	57	5,67E-27	104
ENST00000344347.5	2,73	5,95	XBP1-202	Spliced	P17861-2	100	31	479	509	1	31	4,49E-13	57,2
ENST00000403532.7		1,53	XBP1-203	Unspliced	B1AHH2	100	57	528	584	1	57	5,59E-27	104
ENST00000405219.7	1,76		XBP1-204	Unspliced	B1AHH1	100	57	426	482	1	57	5,17E-27	104
ENST00000611155.4	13,4		XBP1-207	Spliced	P17861-2	100	31	527	557	1	31	7,15E-13	57,2

Figure S13. RNA sequencing of spiperone treated CRC cells revealed XBP1 splicing. PCA Plot displaying the distance between the sample groups after the gene expression profiling. Only Principal Component 1 (X-axis) and Principal

Component 2 (Y-axis) are reported (**a**). PCR analysis of XBP1 splicing; uXBP1, unspliced; sXBP1, spliced form of XBP1 after different time points in HCT116 cells treated with spiperone 2.5 µmol/L (**b**). Heatmap showing unsupervised hierarchical clustering of XBP1 isoforms identified by RNA seq between treated (spip) and untreated (CTR) HCT and CRC-SC# cells (**c**). Table displaying Log2FC and alignment details associated with the identified isoforms of XBP1 (**d**).

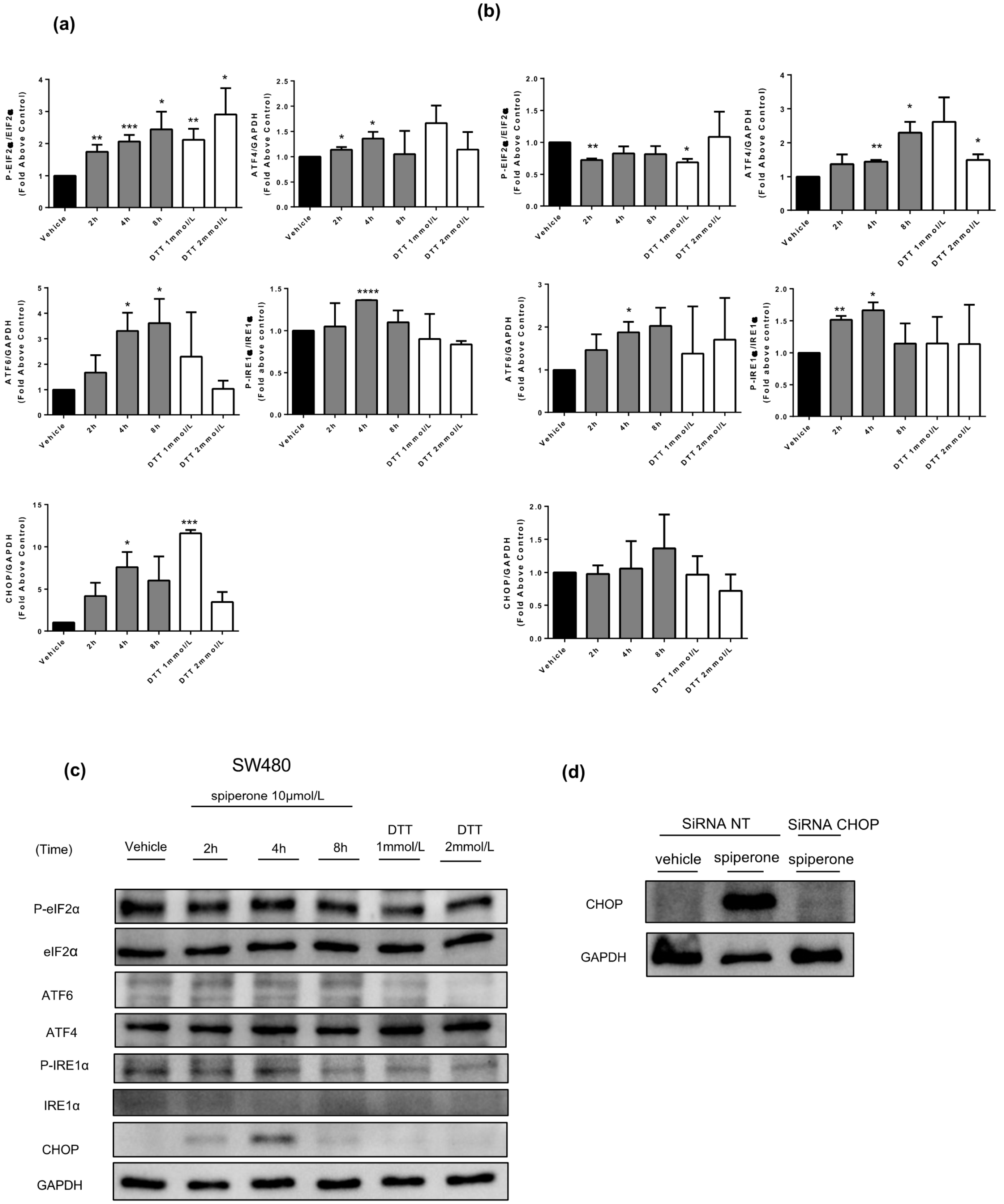

Figure S14. Spiperone induces ER stress in CRC cells. Histogram showing quantification of P-eIF2α, ATF4, ATF6, P-IRE1α, and CHOP on HCT116 (a) and CRC-SC#1 (b). Data are normalized on eIF2α, IRE1α and GAPDH respectively. Western blot analysis of SW480 (c) after 2-, 4- and 8-hours treatment with spiperone. Lysates were analyzed for P-eIF2α, eIF2α, ATF4 ATF6, P-IRE1α, IRE1α, CHOP, and GAPDH. Data are representative images of three independent experiments. Western blot analysis of CHOP protein in HCT116 after CHOP gene silencing and 24 hours treatment with spiperone 10 μmol/L. Lysates were analyzed for CHOP and GAPDH (d). * $p < 0.05$; Student's t-test ** $p < 0.01$; Student's t-test ***$p < 0.001$.

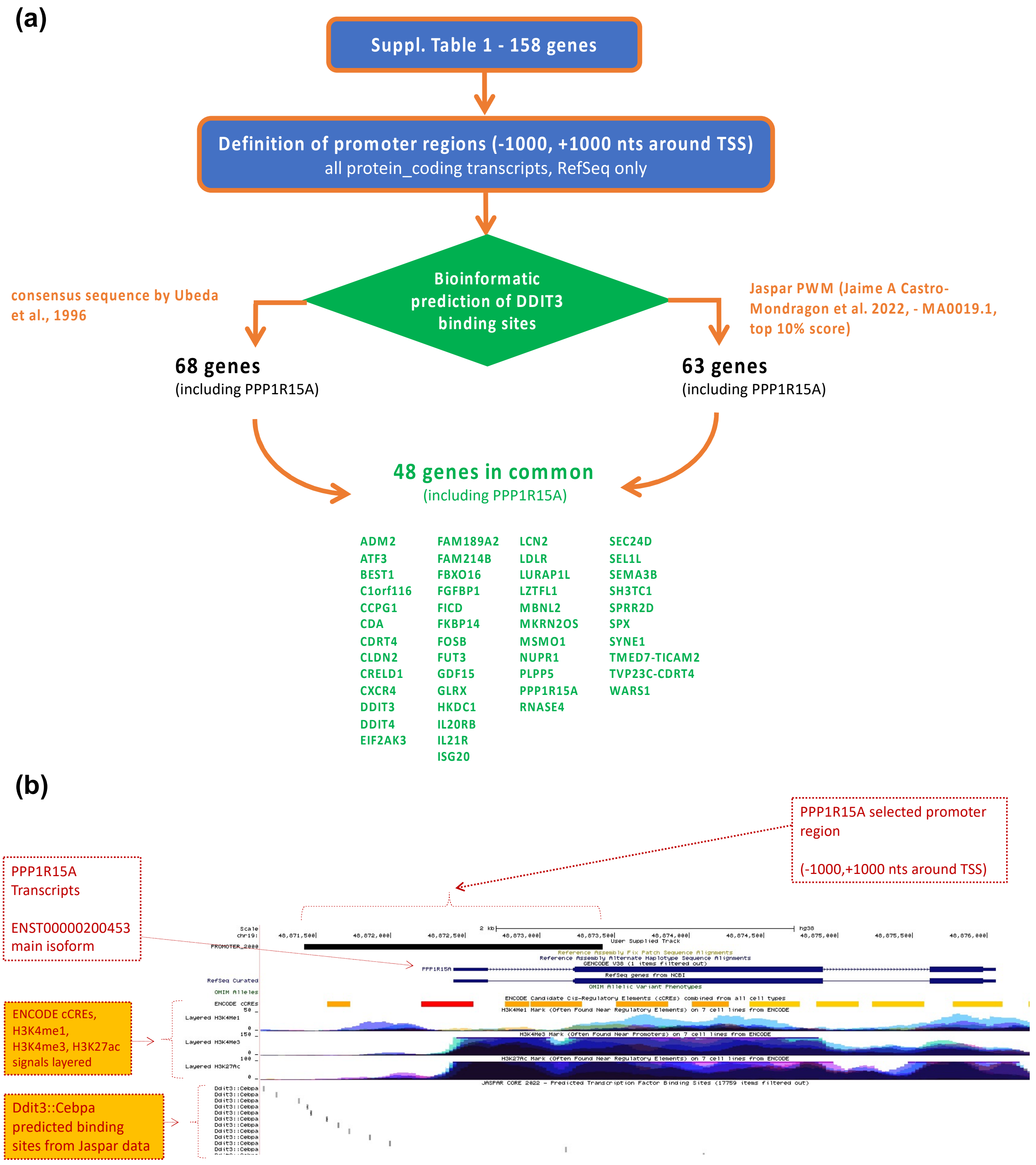

Figure S15. Identification of CHOP/DDIT3 targets. Bioinformatic pipeline for DDIT3 binding site identification and identified targets of CHOP/DDIT3 (**a**). DDIT3 binding site on PPP1R15A (**b**).

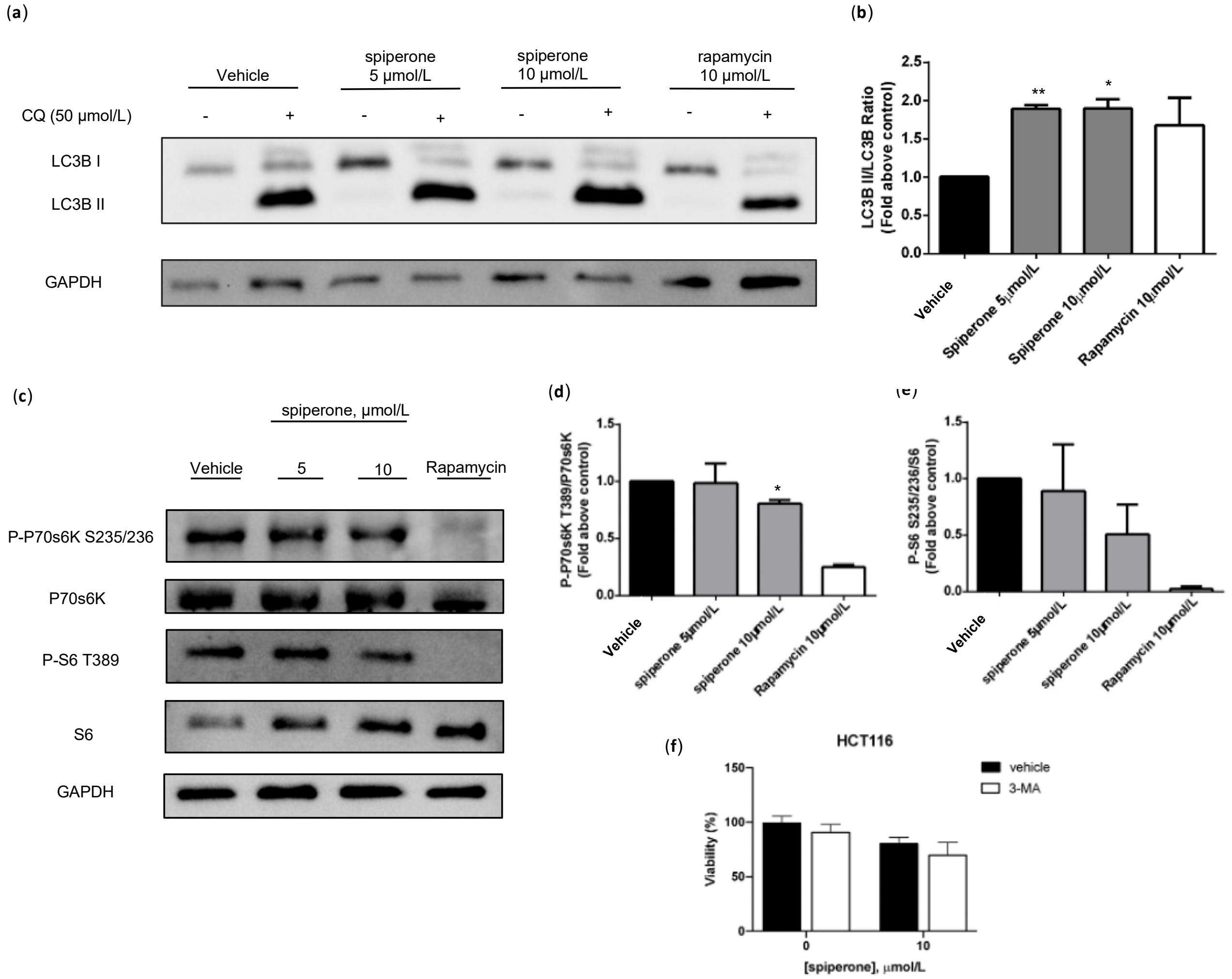

Figure S16. Spiperone induces autophagy and mTOR pathway inhibition. Western blot analysis of HCT116 cells after 16 hours treatment with spiperone. Lysates were analyzed for p-P70S6K T389, P70S6K, P-S6 S235/236, S6, LC3B, and GAPDH. Western blot (**a**) and histogram (**b**) showing the relative expression of LC3B II/I upon chloroquine (CQ) treatment. Western blot (**c**) and histogram showing quantification of P70S6K (**d**) and S6 (**e**) phosphorylation normalized on total protein P70S6K and S6, respectively. Densitometric analyses are expressed as the mean ± SD of three independent experiments. * Student's T-test $p < 0.05$ **, Student's T-test $p < 0.01$. Effect of co-treatment of spiperone and autophagy inhibitor 3-MA 1 mmol/L (**f**). After 30 minutes of pretreatment, HCT116 cells were treated with vehicle or spiperone at 10 µmol/L. Graphs displaying cell viability as the percentage of viable cells. Data show mean ± SD of at least three independent experiments performed in triplicate.

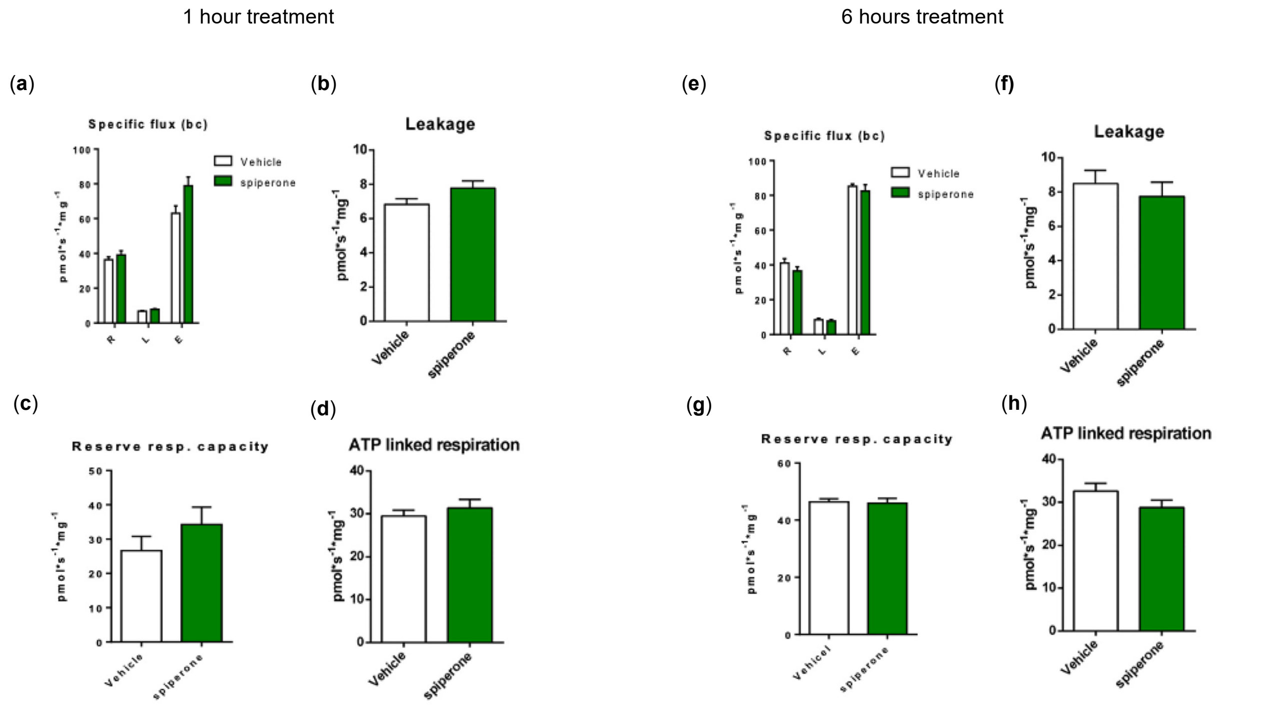

Figure S17. Spiperone treatment alters mitochondrial respiration. HCT116 cells were treated with spiperone 10 µmol/L for 1 and 6 hours before OCR evaluation. Graph displaying oxygen flux in the routine state (R), in the leakage state (L) after addition of the ATP synthetase inhibitor, oligomycin, and after the addition of the uncoupler of oxidative phosphorylation, FCCP, to quantify maximum respiratory capacity (E) (**a, e**). All data are expressed as specific flux, i.e., oxygen consumption normalized to the sample protein content and after non-mitochondrial oxygen flux subtraction (ROX). Histogram showing leakage state of the respiratory chain (**b, f**). Histogram showing oxygen consumption linked to ATP production, i.e., oligomycin-sensitive respiration obtained by the subtraction of L from R (**c, g**). Reserve respiratory capacity obtained by the subtraction of R+P from E (**d, h**). All data are expressed as mean ± SEM from 4 independent experiments.

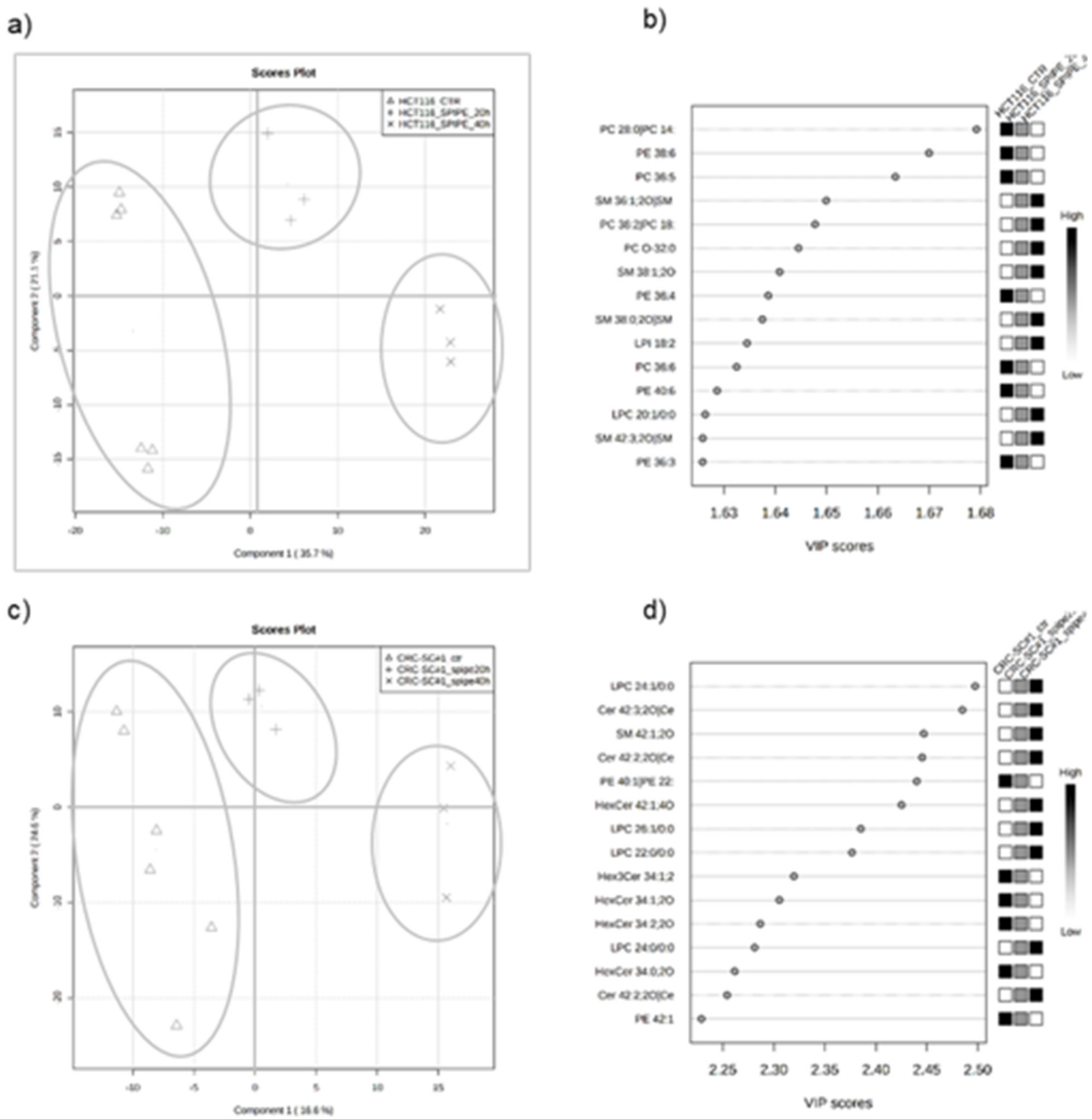

Figure S18. Lipidomic results in CRC cells treated with spiperone for 20 and 40 hours. PLS-DA score plot of HCT116 and (**a**) CRC-CS#01 (**c**) cells and relative VIP analysis of component 1 reporting the first 15 ranked lipid species that are significantly different in treated vs control HCT116 (**b**) and CRC-CS#01 (**d**) cells.

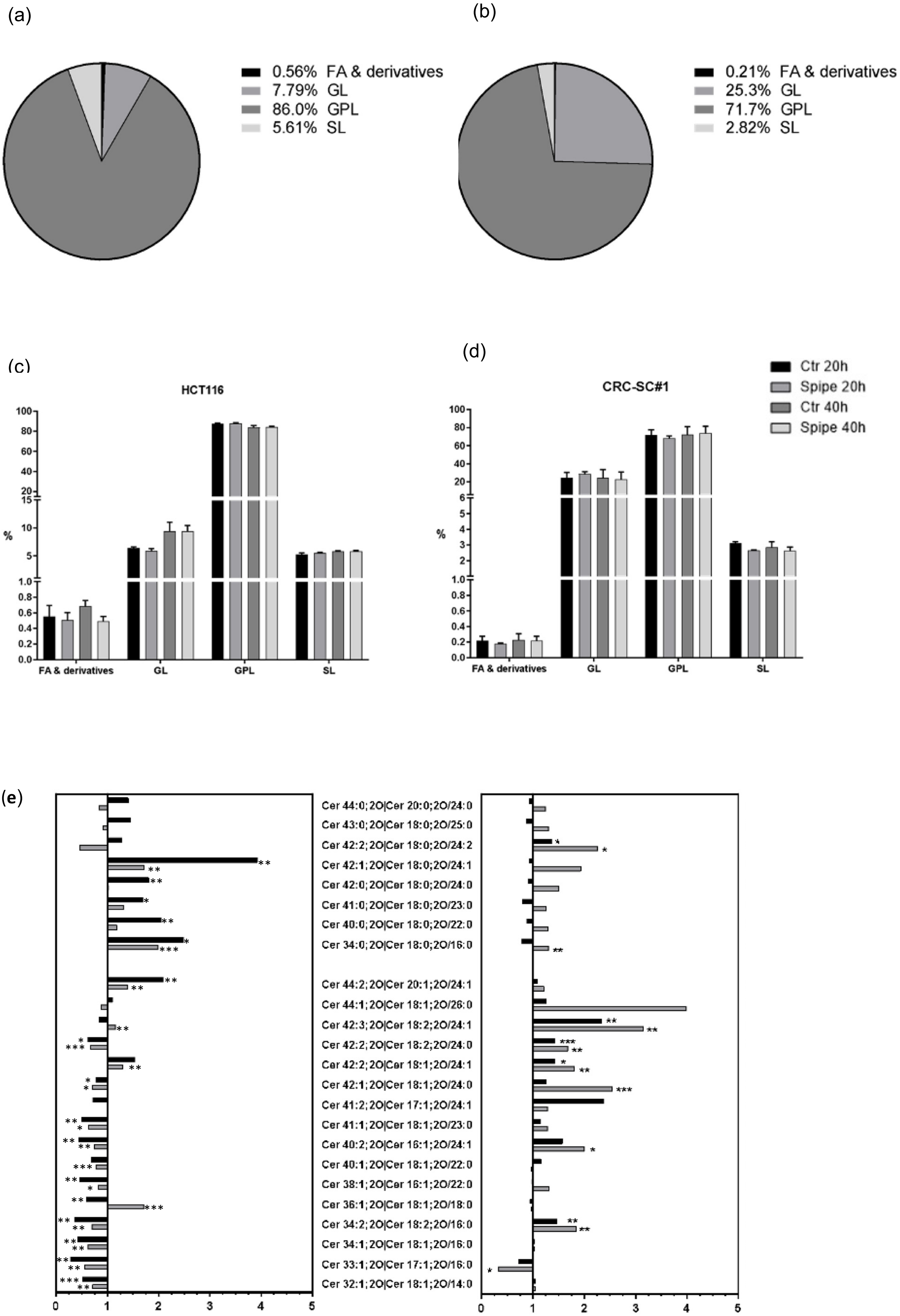
(a)
0.56% FA & derivatives
7.79% GL
86.0% GPL
5.61% SL
(b)
0.21% FA & derivatives
25.3% GL
71.7% GPL
2.82% SL
(c)
HCT116
%
FA & derivatives
GL
GPL
SL
(d)
CRC-SC#1
Ctr 20h
Spipe 20h
Ctr 40h
Spipe 40h
%
FA & derivatives
GL
GPL
SL
(e)
Cer 44:0;2O|Cer 20:0;2O/24:0
Cer 43:0;2O|Cer 18:0;2O/25:0
Cer 42:2;2O|Cer 18:0;2O/24:2
Cer 42:1;2O|Cer 18:0;2O/24:1
Cer 42:0;2O|Cer 18:0;2O/24:0
Cer 41:0;2O|Cer 18:0;2O/23:0
Cer 40:0;2O|Cer 18:0;2O/22:0
Cer 34:0;2O|Cer 18:0;2O/16:0
Cer 44:2;2O|Cer 20:1;2O/24:1
Cer 44:1;2O|Cer 18:1;2O/26:0
Cer 42:3;2O|Cer 18:2;2O/24:1
Cer 42:2;2O|Cer 18:2;2O/24:0
Cer 42:2;2O|Cer 18:1;2O/24:1
Cer 42:1;2O|Cer 18:1;2O/24:0
Cer 41:2;2O|Cer 17:1;2O/24:1
Cer 41:1;2O|Cer 18:1;2O/23:0
Cer 40:2;2O|Cer 16:1;2O/24:1
Cer 40:1;2O|Cer 18:1;2O/22:0
Cer 38:1;2O|Cer 16:1;2O/22:0
Cer 36:1;2O|Cer 18:1;2O/18:0
Cer 34:2;2O|Cer 18:2;2O/16:0
Cer 34:1;2O|Cer 18:1;2O/16:0
Cer 33:1;2O|Cer 17:1;2O/16:0
Cer 32:1;2O|Cer 18:1;2O/14:0

Figure S19. Representation of the relative percentage (%) of the major lipid classes identified by HPLC-MS/MS. Pie diagram of lipid classes representation (%) in HCT116 (a) and CRC-SC#1 (b) cells. Representation of the relative percentage (%) of the major the lipid classes after 20- and 40-hours treatment with spiperone 10 μmol/L, in HCT116 cells (c) and in CRC-SC#1 cells (d); FA, fatty acids, GL, glycerolipids, GLP, glycerophospholipids, SL sphingolipids. Cer species characterization in CRC cells treated for 20 hours and 40 hours (e). Student's t-test * $p < 0.05$; Student's t-test ** $p < 0.01$; Student's t-test ***$p < 0.001$.

Figure 8 a

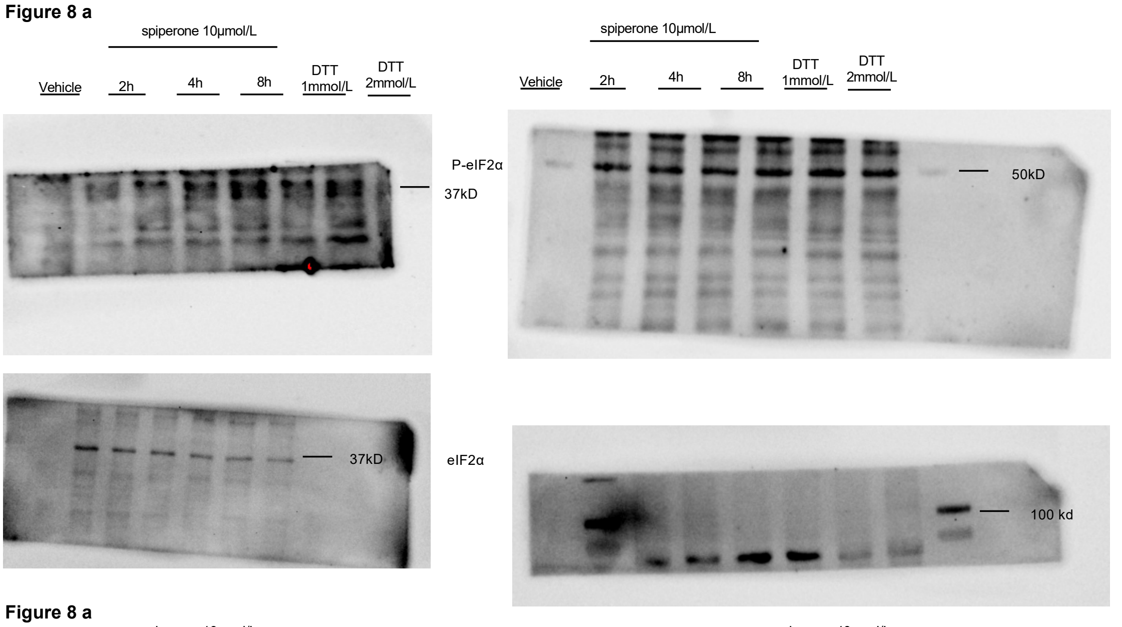

Figure 8 a

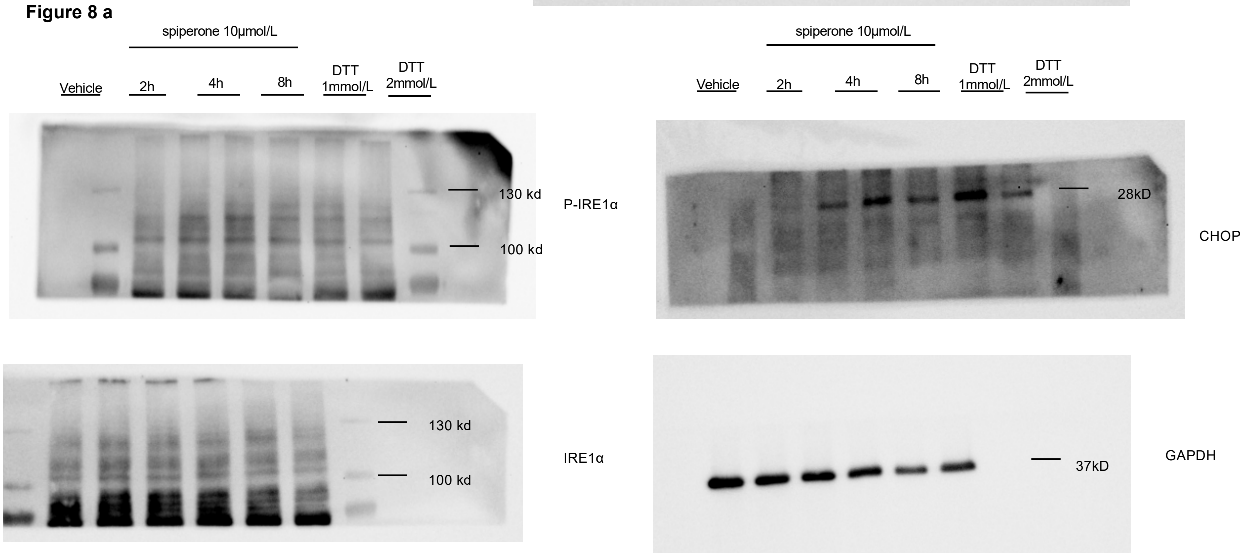

Figure 8b

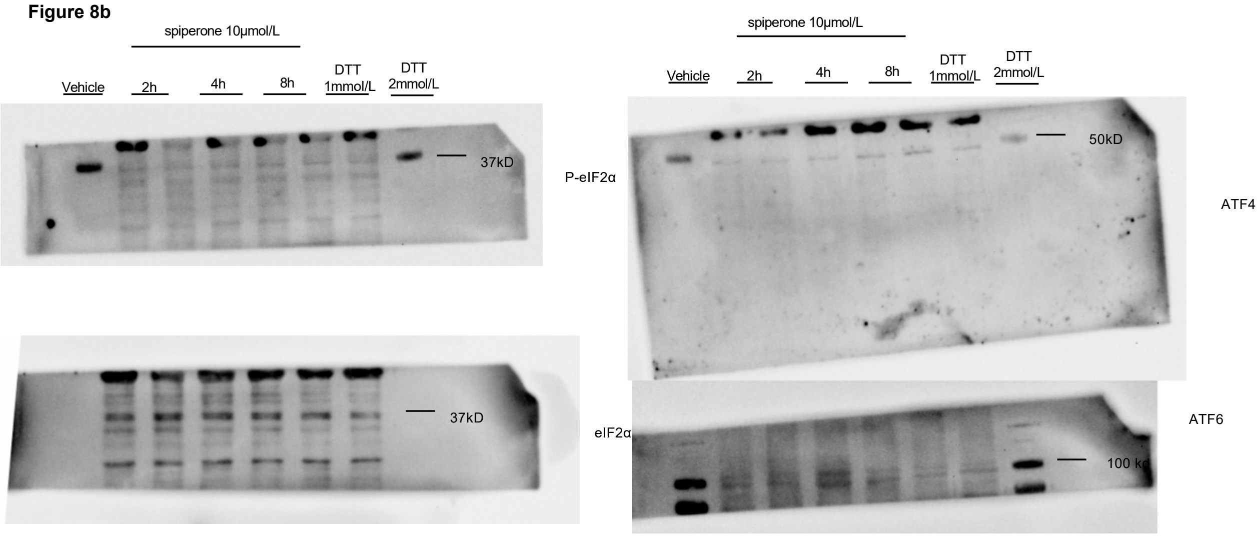

Figure 8b

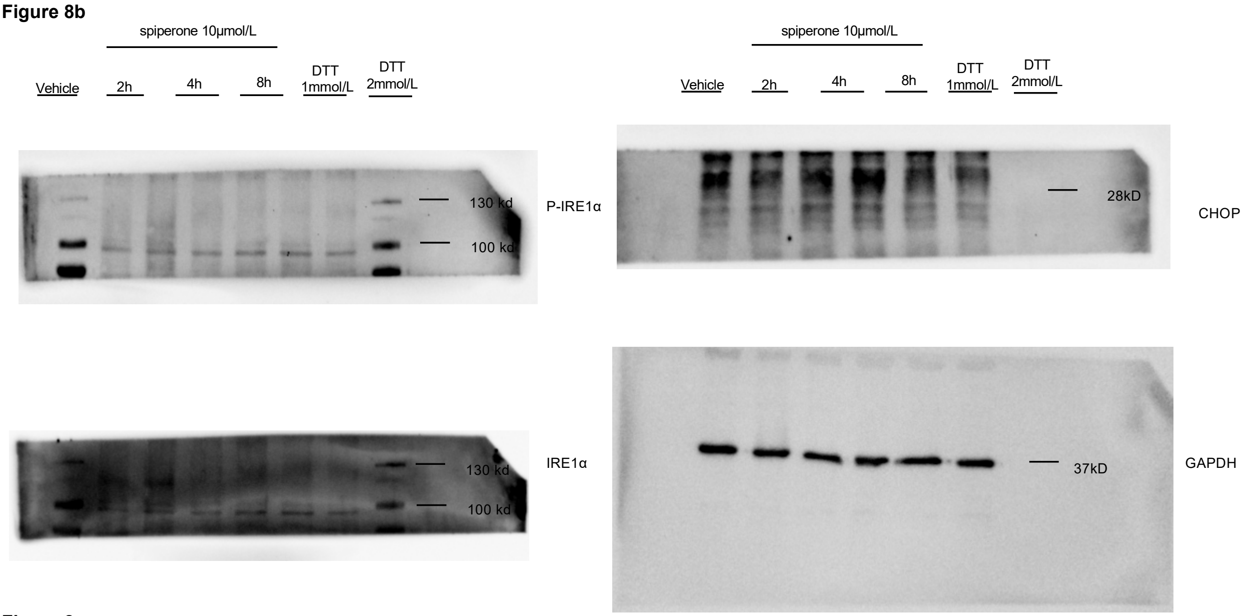

Figure 8c

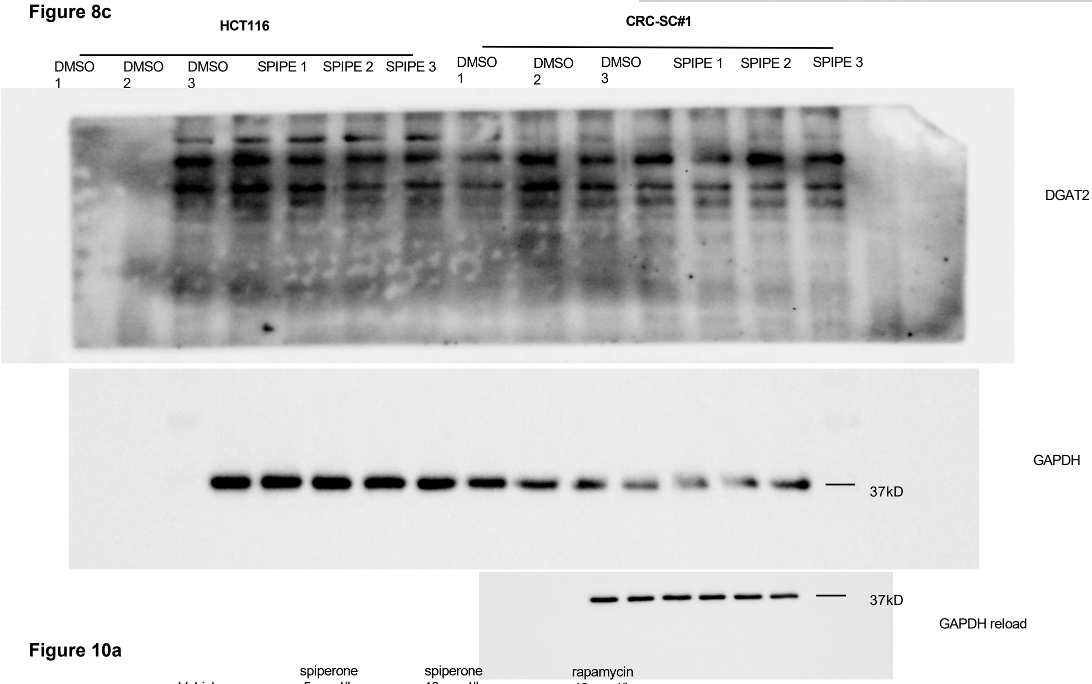

Figure 10a

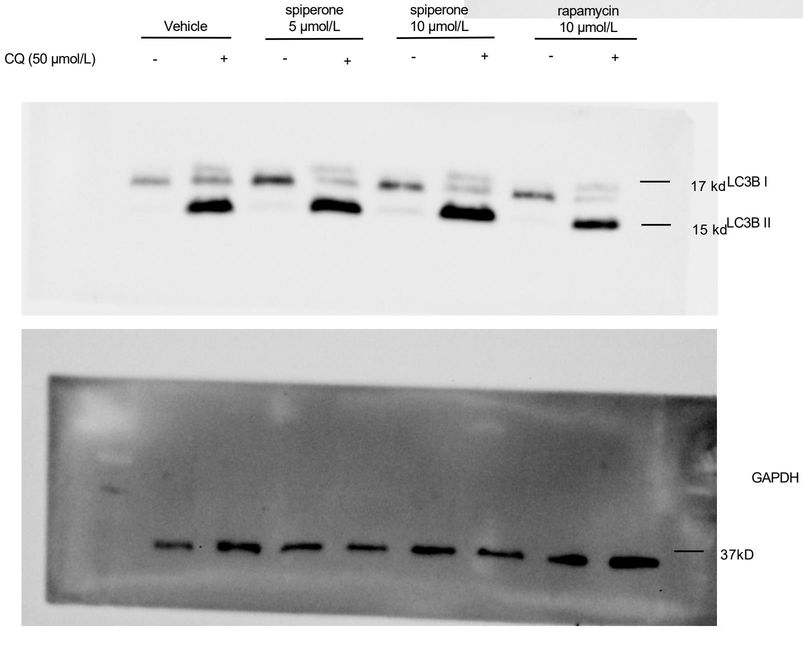

Figure 10c

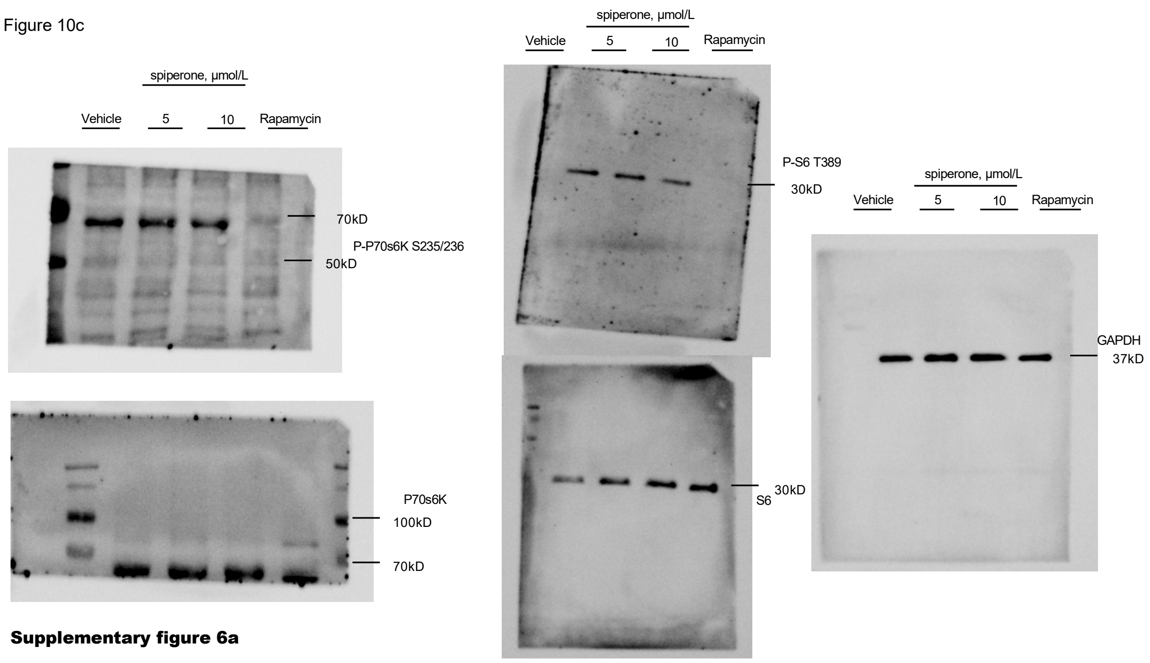

Supplementary figure 6a

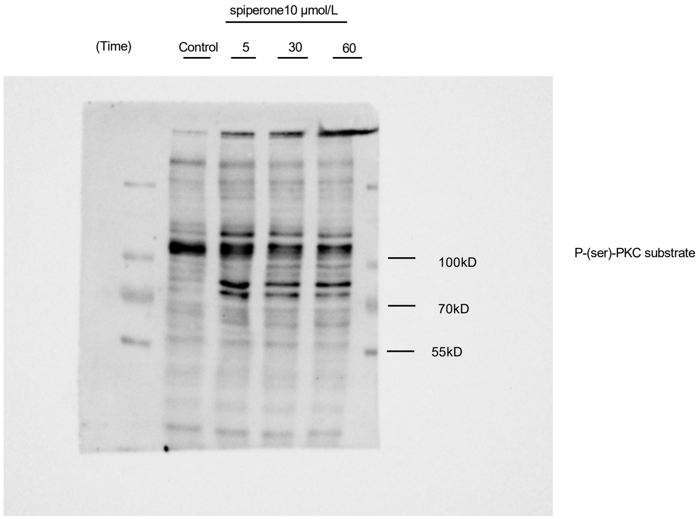

Supplementary figure 6b

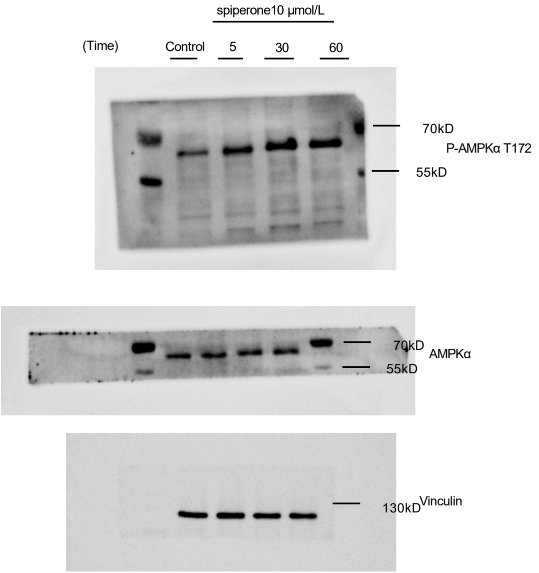

Supplementary figure 6c

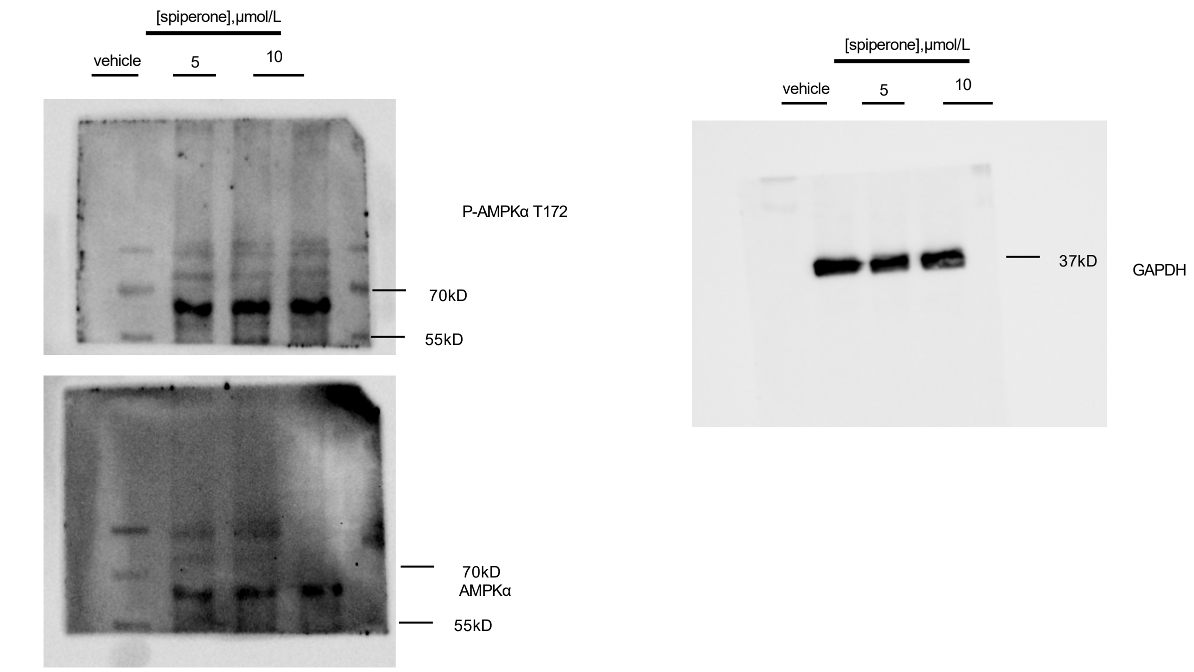

Supplementary figure 8b

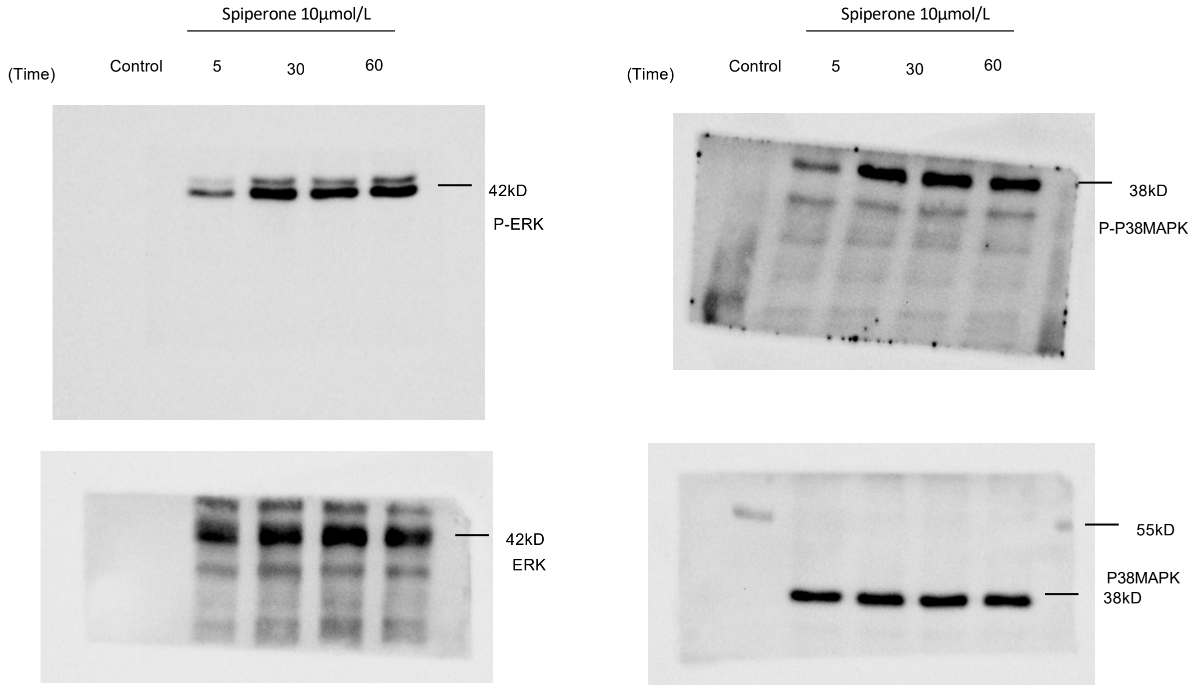

Supplementary figure 8b

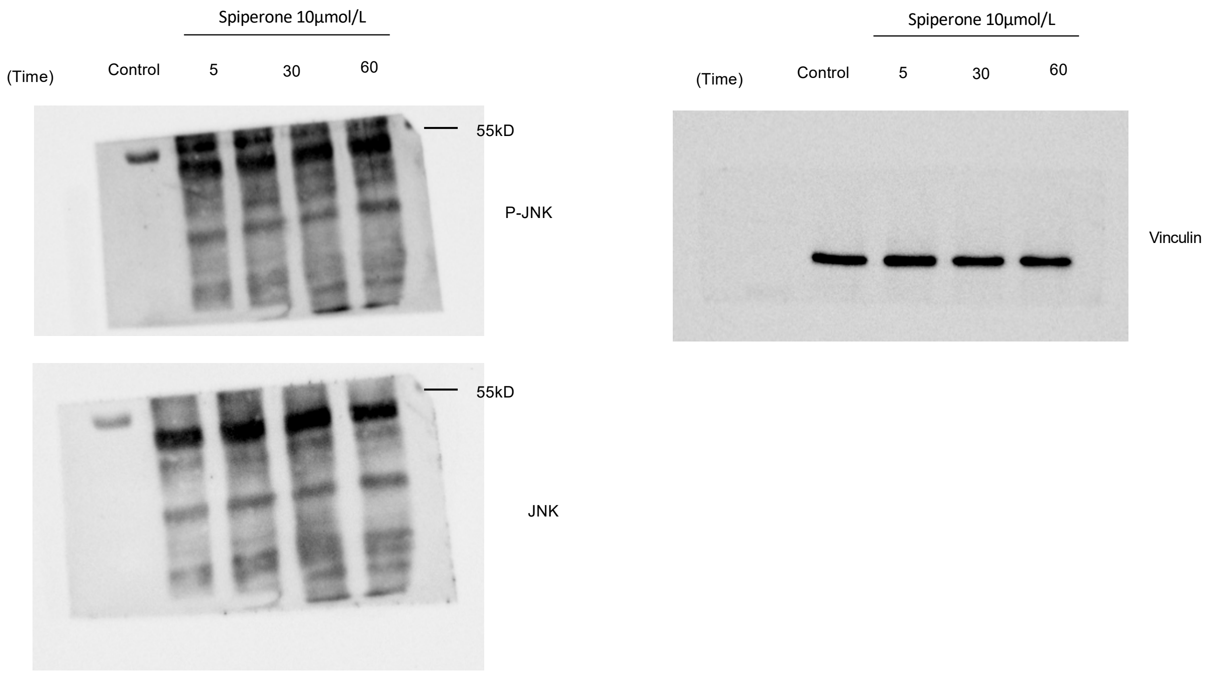

Supplementary figure 8c

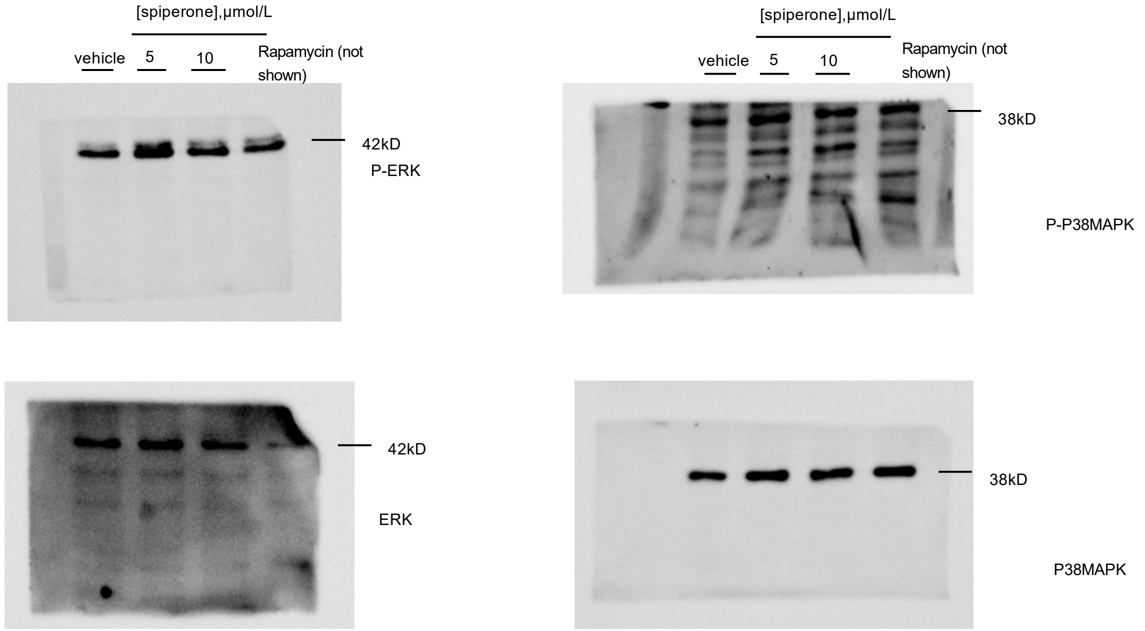

Supplementary figure 8c

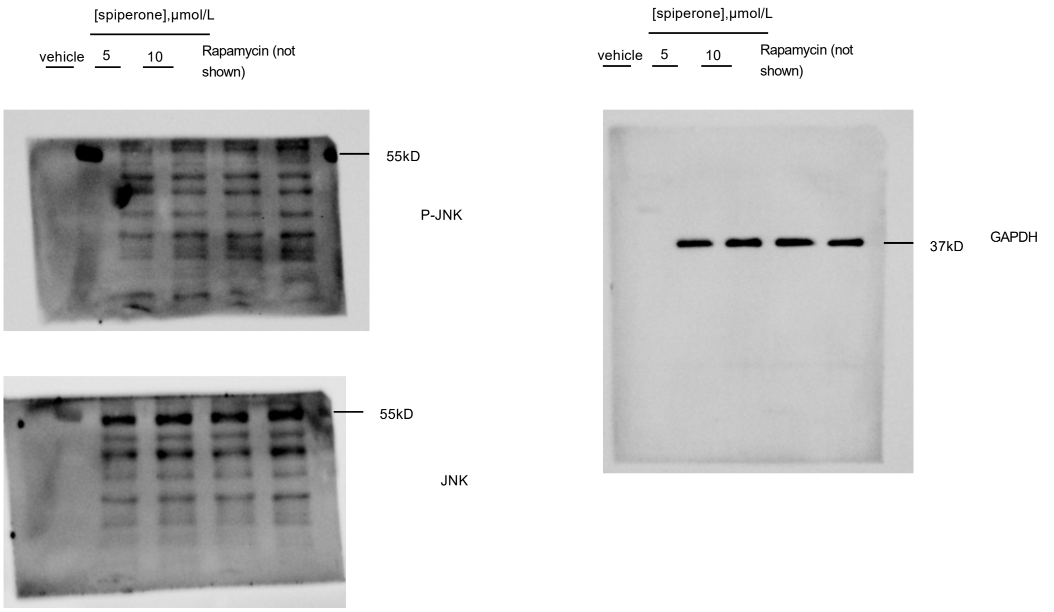

Supplementary figure 12

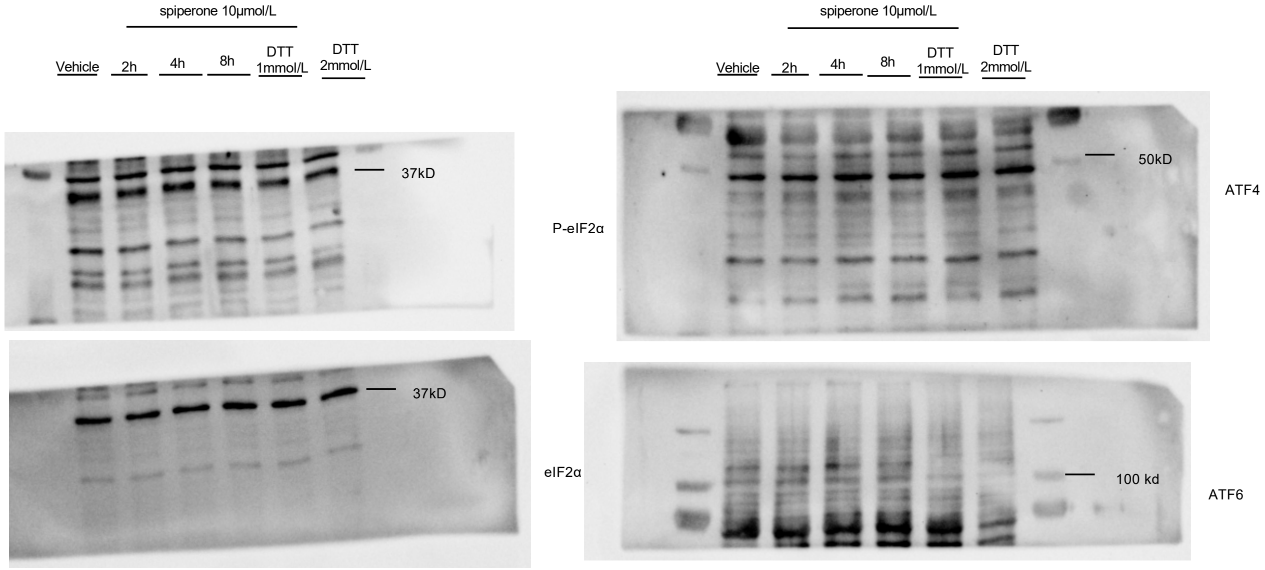

Supplementary figure 12

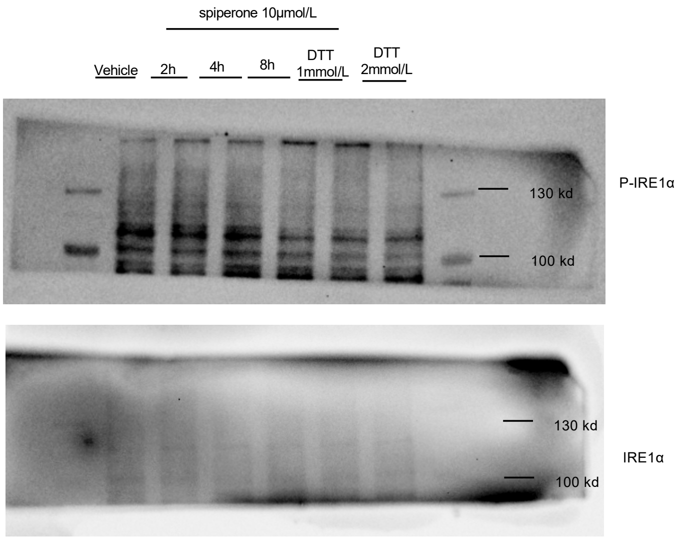

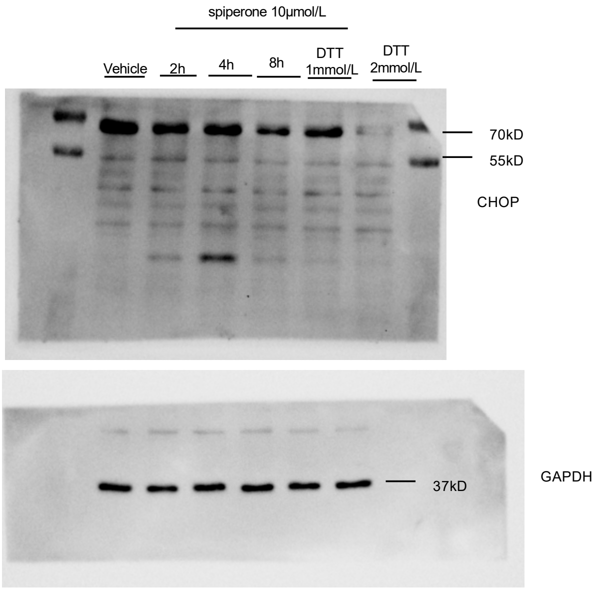

Supplementary figure 12c

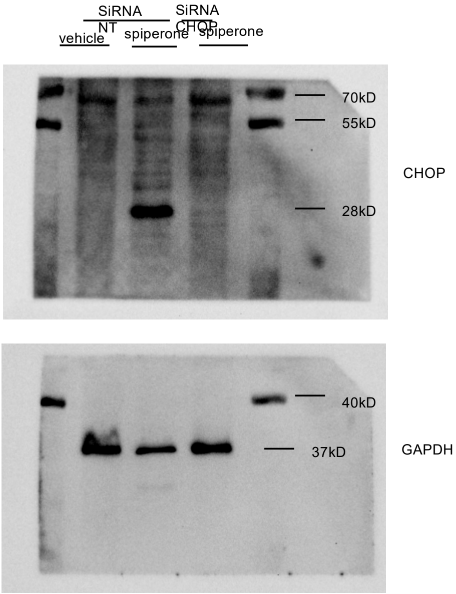

Figure S20. Western blot membranes.
